# A Water-Stable Boronate Ester Cage

**DOI:** 10.1021/jacs.3c12002

**Published:** 2024-02-07

**Authors:** Philipp H. Kirchner, Louis Schramm, Svetlana Ivanova, Kazutaka Shoyama, Frank Würthner, Florian Beuerle

**Affiliations:** † Institut für Organische Chemie, Julius-Maximilians-Universität Würzburg, Am Hubland, Würzburg 97074, Germany; ‡ Center for Nanosystems Chemistry (CNC), Julius-Maximilians-Universität Würzburg, Theodor-Boveri-Weg, Würzburg 97074, Germany; § Institut für Organische Chemie, Eberhard Karls Universität Tübingen, Auf der Morgenstelle 18, Tübingen 72076, Germany

## Abstract

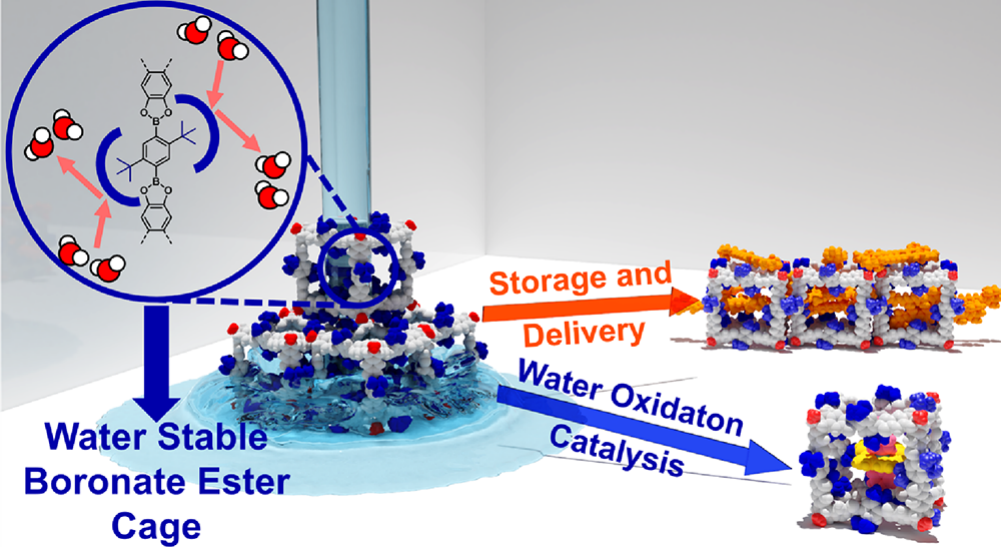

The reversible condensation of catechols and boronic acids to boronate esters is a paradigm reaction in dynamic covalent chemistry. However, facile backward hydrolysis is detrimental for stability and has so far prevented applications for boronate-based materials. Here, we introduce cubic boronate ester cages **6** derived from hexahydroxy tribenzotriquinacenes and phenylene diboronic acids with *ortho*-*t*-butyl substituents. Due to steric shielding, dynamic exchange at the Lewis acidic boron sites is feasible only under acid or base catalysis but fully prevented at neutral conditions. For the first time, boronate ester cages **6** tolerate substantial amounts of water or alcohols both in solution and solid state. The unprecedented applicability of these materials under ambient and aqueous conditions is showcased by efficient encapsulation and on-demand release of β-carotene dyes and heterogeneous water oxidation catalysis after the encapsulation of ruthenium catalysts.

## Introduction

**Figure 1:**
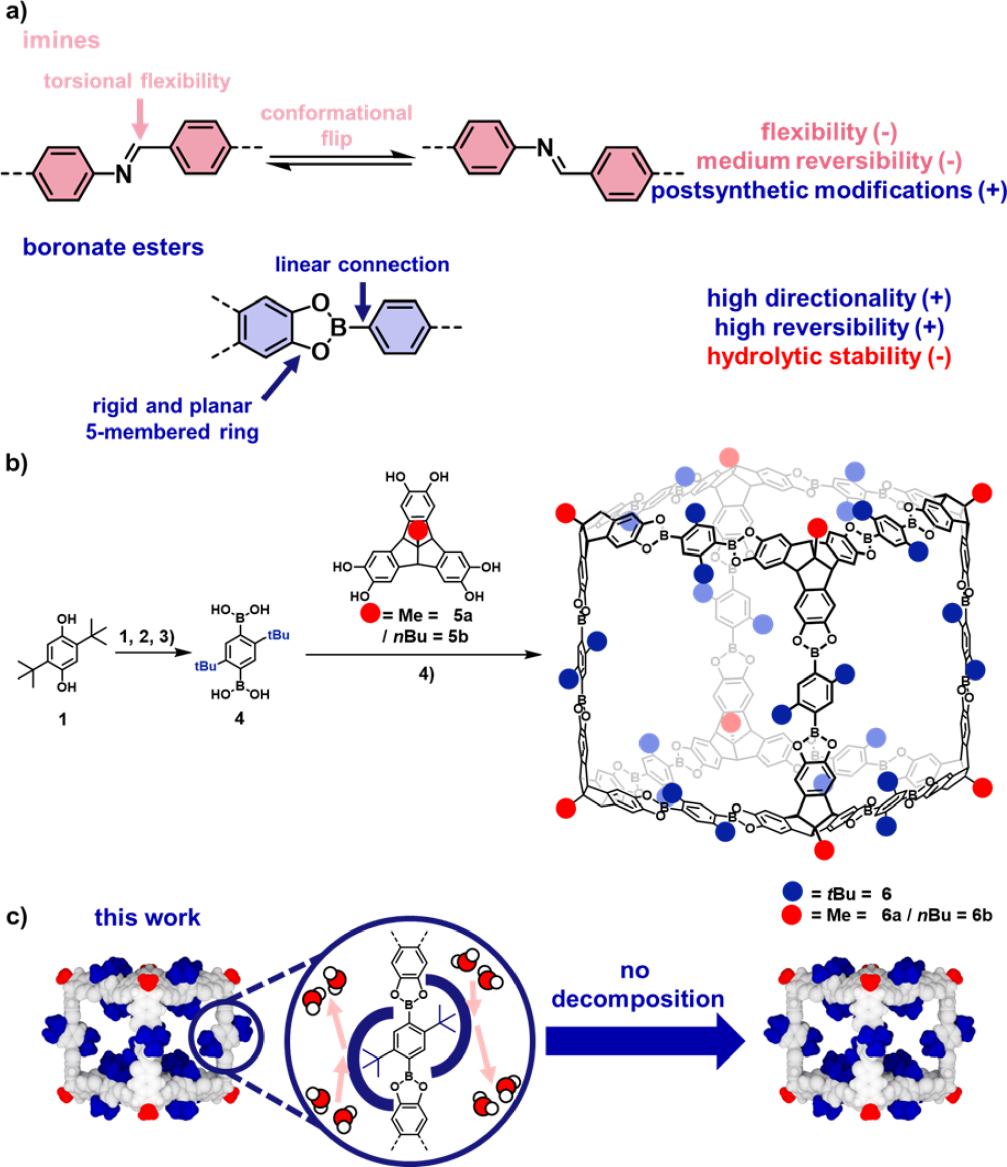
**|** (a) Comparison of imines and boronate esters as privileged linkages in dynamic covalent chemistry with their desirable (+) and problematic (−) properties. (b) Synthesis of cubic cages **6** from TBTQs **5** and BDBA-*t*-Bu **4**: (1) Tf_2_O, pyridine, CH_2_Cl_2_, 0 °C → RT, 2 h, 94%, (2) Pd(dppf)Cl_2_, NMe_3_, HBpin, molecular sieves 4 Å, 1,4-dioxane, N_2_, 120 °C, 5 h, 92%, (3) BBr_3_, CH_2_Cl_2_, 0 °C → RT, 18 h, 84%, (4) AcOH, molecular sieves 4 Å, THF, RT, 7–10 d, 30%. (c) Concept of steric overcrowding in *t*-Bu-functionalized boronate esters for highly stable dynamic covalent materials.

The synthesis of artificial molecular containers with nanometer-sized pores remains a huge challenge. Traditionally, multistep procedures involving irreversible reactions suffer from low yield and selectivity or kinetic trapping of *off*-pathway side products. Only by the advent of dynamic covalent chemistry (DCC)^1,2^ did the instantaneous formation of highly complex molecular architectures from many small-molecule precursors become synthetically feasible.^3^ In reminiscence of the folding funnel proposed for protein biosynthesis,^4,5^ shape-persistent cages emerge under thermodynamic control and with exceptional selectivity via dynamic and transient *off*- and *on*-pathway intermediates. Covalent yet dynamic bonding motifs that have so far been used for the synthesis of porous cages and/or polymeric covalent organic frameworks (COFs) include imines,^6−9^ boronate esters,^10,11^ boroxines,^12,13^ disulfides,^14,15^ acetals,^16−18^ oximes,^19^ alkynes,^2^ or carboxylic esters.^18,20^ The major drawback of dynamic covalent structures is, however, the poor stability under ambient conditions, which has so far impeded practical applications. Since DCC is predominantly based on reversible condensation reactions, even traces of humidity or protic solvents, e.g., H_2_O and alcohols, might induce back reactions and thus decomposition of the materials. For imine-based systems, intramolecular hydrogen bonding^21,22^ or postsynthetic conversion into more stable linkages, e.g., amines,^23^ amides,^24,25^ and carbamates,^26^ significantly improve stability. In combination with the higher inherent stability, imine chemistry has evolved as the dominant reaction for cages and COFs to date. In contrast, no postassembly transformations are available to stabilize boronate esters, which has severely limited the use of this highly dynamic but labile motif in dynamic covalent self-assembly. Still, this rigid linkage offers some distinct structural advantages that cannot be fully compensated for by less stiff imines. The lateral shift, torsional motion, and conformational switchability of C=N double bonds induces significant flexibility, which limits reliable structure prediction and hampers formation of very large but still shape-persistent pores (Figure 1a, top). By contrast, the connecting five-membered ring in boronate esters derived from catechols and boronic acids constitutes a highly linear and planar conjunction predestined for directional self-assembly (Figure 1a, bottom). In recent years, the unique boronate ester motif has been implemented in a growing number of highly versatile structures and complex 3D architectures.^27^ Selected examples include fluorinated cages^28^ or a giant [8 + 12] cage with an additional exoskeleton constructed via postsynthetic alkene metathesis^29^ from the Mastalerz group. Martín and co-workers demonstrated the importance of adaptable precursors for efficient cage synthesis^30^ or the structural switch between boroxine and boronate ester cages.^31^ The transition between trigonal boronate and tetragonal borate structures was used for stimuli-responsive guest release in supramolecular capsules from the Iwasawa group.^32^ We recently utilized rigid boronate esters for the shape-selective synthesis of trigonal−bipyramidal, tetrahedral, or cubic cages from orthogonal hexahydroxy tribenzotriquinacenes (TBTQs) and diboronic acids with varying bite angles. Furthermore, we extended this approach toward an isoreticular series of highly porous cubic cage crystals, in which isostructural packing is maintained by π–π interactions between the linear boronate ester edges.^33^ Together with the triptycene-based cuboctahedral cages from the Mastalerz group,^10^ these crystalline materials set the benchmark for the most porous cages reported so far with BET surface areas exceeding 3000 m^2^ g^–1^. Based on this literature precedent, it becomes obvious that stabilizing boronate esters while retaining structural robustness is an eagerly desired yet still not realized task in DCC. Here, we present cubic boronate ester cages **6** with bulky *t*-Bu groups in the *ortho*-position to the boron centers featuring unprecedented stability even in pure water. The pronounced steric shielding effectively blocks the exchange of oxygen substituents via tetrahedral extension at the Lewis acidic boron sites under neutral conditions, which is only activated under acid or base catalysis. For the first time, boronate ester cages **6** tolerate substantial amounts of water or alcohols both in solution and solid state. To showcase the enormous potential of these materials for applications under ambient conditions or in aqueous media, porous crystals of cage **6a** have been efficiently loaded with β-carotene molecules. While encapsulation is facilitated by stabilizing aggregation of the aromatic dyes in the pores, on-demand delivery of the molecular cargo can be triggered via a strong acid stimulus. Furthermore, cages **6a** have been investigated as heterogeneous water oxidation catalysts after encapsulation of ruthenium complexes.

## Results and Discussion

### Synthesis and Characterization of Boronate Ester Cages

Recently, we reported the synthesis of organic nanocubes via the cross-condensation of hexahydroxy TBTQs and linear diboronic acids via a dynamic covalent approach.^34^ Depending on the apical substituent at the TBTQs, either soluble (R^1^ = *n*-Bu) or crystalline (R^1^ = Me) cages were obtained. By modifying the 2,5-positions of (1,4-phenylene)diboronic acids (BDBAs) with linear alkyl chains (R^2^ = Me, Et, *n*-Bu), an isoreticular series of cage crystals were isolated with exceptionally high porosity up to 3426 m^2^ g^–1^.^33^ Building on these exciting findings, we planned to modulate crystal packing and/or fine-tune the pore structure by implementing bulky *t*-Bu groups in BDBA-*t*-Bu **4** as a constitutional isomer of the well-established linker BDBA-*n*-Bu. Initially, we targeted **4** via the established sequence of dibromination on 2,5-di-*t*-butylbenzene followed by 2-fold Miyaura borylation and deprotection with BBr_3_.^34,35^ While the bromination was successful,^36^ at most, traces of the envisioned dioxaborolane **3** (Figure S1) were obtained even after extensive screening of borylation procedures. We attribute the poor reactivity to the high steric demand of the bulky *t*-Bu groups, which already suggests slow kinetics for manipulations in *ortho*-position. Nevertheless, **4** was accessible in excellent yields through smooth borylation and subsequent deprotection starting from the more reactive 1,4-ditriflate **2** (Figure S1), which was synthesized from diol **1** according to a literature procedure (Figure 1b).^37^ Having **4** at hand, we approached the formation of cubic cages via dynamic covalent reactions with corner units **5a**(38) or **5b**(38) (for synthesis, see Figure S1). Following our established protocol,^33^ precursors **5** and **4** were dissolved in dry THF and activated molecular sieves 4 Å were added. Typically, instantaneous formation of boronate esters takes place in THF and full conversion into cages is observed after several days, on condition that intermediate assemblies show sufficient solubility.^33^ To our surprise, however, reaction monitoring by ^1^H NMR in deuterated THF indicated complete inhibition of boronate ester formation since the signals for both starting materials did not vanish even after 10 days at room temperature (Figure S17).^11^ Furthermore, only very little condensation but no cage formation was observed at elevated temperatures up to 130 °C and Dean–Stark conditions or for reactions in different solvents such as MeCN, toluene, or EtOAc (Figure S2).

Whereas this lack of reactivity for **4** seems detrimental to cage formation, it might, on the other hand, provide unprecedented stability once the final assembly would be formed under more forcing conditions. According to the literature,^39^ exchange of oxygen substituents at trigonal boronic acids is accelerated by acid or base catalysis. Indeed, test reactions in the presence of either acid, e.g., trifluoroacetic acid (TFA), AcOH, or base, e.g., KOH, finally indicated transformation of the precursors into boronate esters by a characteristic downfield shift of the signals for the TBTQ bridgehead protons (Figure S18). Further optimization (Table S1) identified catalytic amounts (0.15 equiv) of AcOH as the most suitable catalyst for the isolation of crystalline cage **6a** after 7–10 days (see the synthesis section in the SI for more details). The synthesis of **6b** is analogous but requires longer reaction times of up to 14 days. Stronger TFA also induces cage formation; however, the conversion is too fast due to the higher acidity and yields only amorphous material.

**Figure 2:**
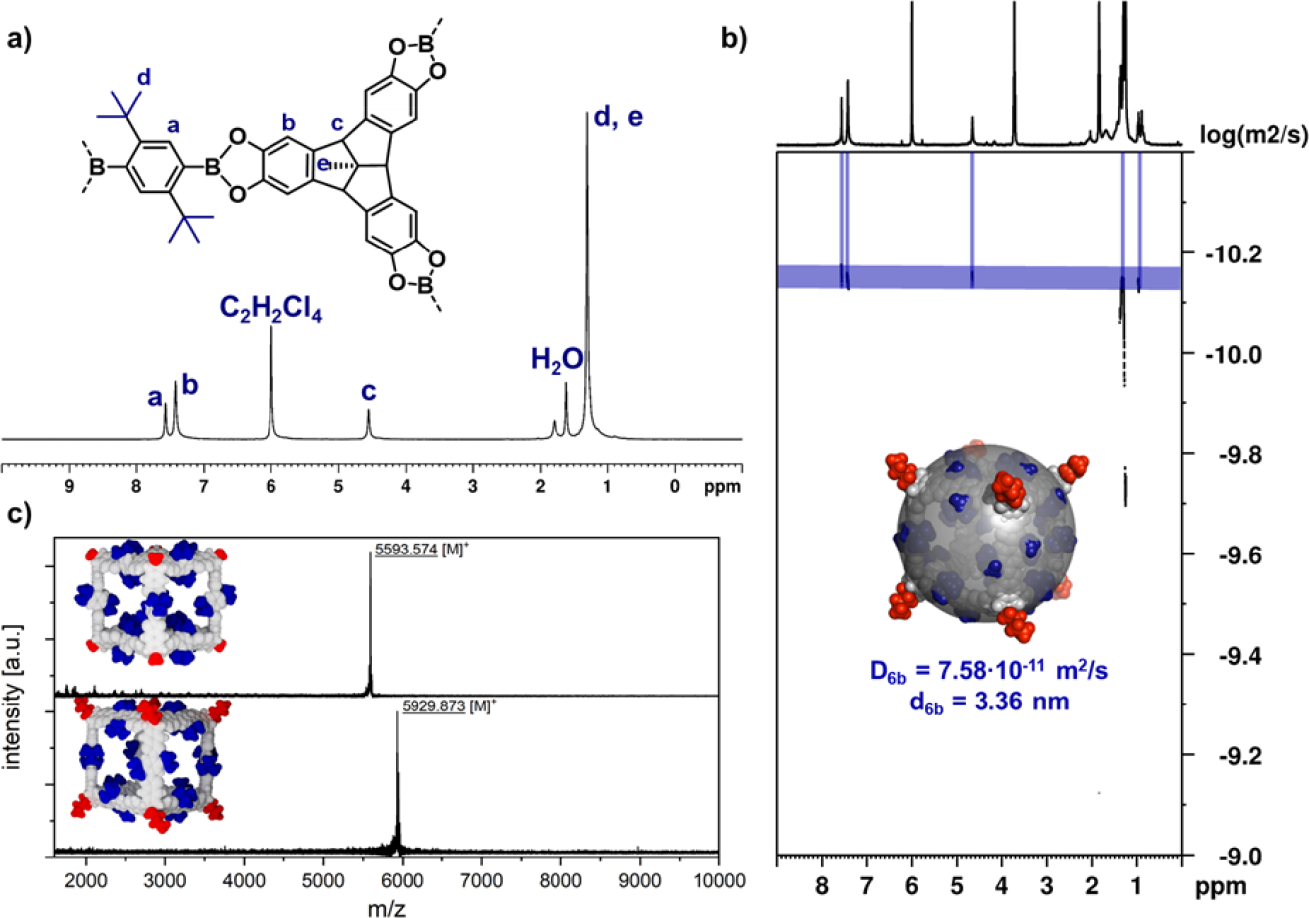
**|** Solution characterization by (a) ^1^H NMR (400 MHz, RT, C_2_D_2_Cl_4_) for **6a**, (b) DOSY-NMR (400 MHz, DSTE, RT, C_2_D_2_Cl_4_) for **6b**, and (c) MALDI-TOF MS for **6a** and **6b** (CHCl_3_/THF, DCTB, positive mode).

Purification of the crude crystalline product is easily achieved by washing with copious amounts of CHCl_3_, THF, and MeOH to remove traces of unreacted precursors, oligomeric fragments, and acid residues, which would otherwise lead to decomposition of the isolated cage in a concentrated solution or suspension. Remarkably, **6a** is completely inert against the protic solvent MeOH, which instantaneously dissolves regular boronate ester cages into monomeric building blocks (Figure 1c). Despite the considerably lower solubility compared to the isomeric *n*-Bu cage **7b** (R^1^ = R^2^ = *n*-Bu),^34^^1^H NMR spectroscopy in C_2_D_2_Cl_4_ (Figure 2a and Figure S10) in combination with MALDI-TOF mass spectrometry (MS) (Figure 2c) unequivocally confirmed the exclusive formation of highly symmetrical [8 + 12] cubic cages **6**. The solubilizing *n*-Bu groups at the TBTQ units also allowed DOSY-NMR in C_2_D_2_Cl_4_ for **6b** (Figure 2b), which revealed a diffusion coefficient of 8.10 × 10^–11^ m^2^ s^–1^ and a solvodynamic diameter of 3.36 nm according to the Stokes–Einstein equation, being in good accordance with other alkylated cubic cages.^33^

### Solid-State Characterization

**Figure 3:**
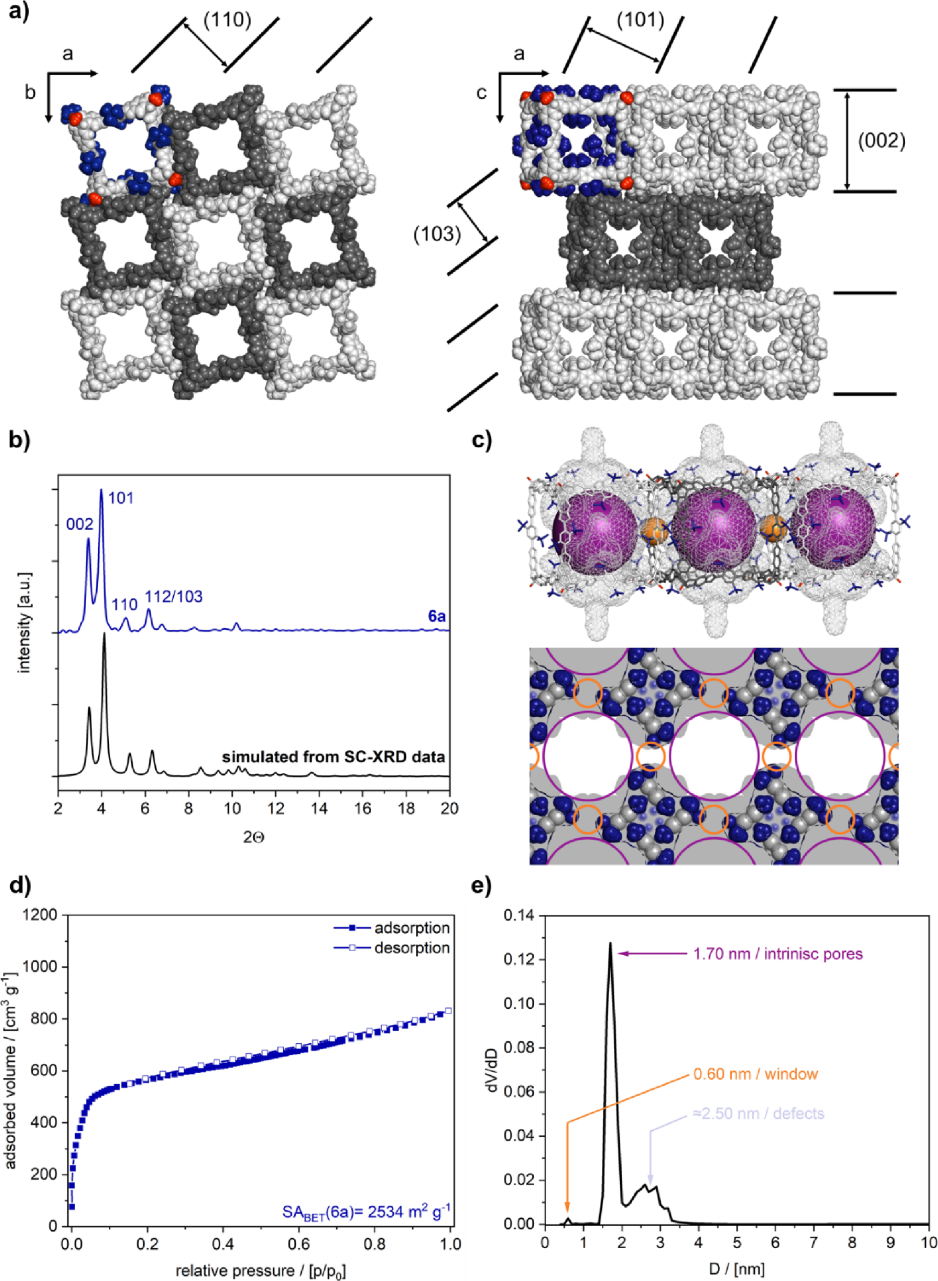
**|** (a) SC-XRD structure of **6a** with views of one layer along the *c*-axis (left) and stacked layers along the *b*-axis (right). (b) PXRD data for crystalline **6a** (blue) and simulation from SC-XRD structure (black). (c) Thin section cut of the pore system indicating the cage windows (orange) and intrinsic pores (purple), the occupied area after 360° rotation along the channel axis is indicated in gray. (d) N_2_ sorption isotherm for **6a** at 77 K. (e) Pore size distribution for **6a** calculated with a MDFT carbon kernel for cylindrical pores.

For **6a**, square-shaped plate crystals were isolated directly from the reaction mixture. Despite the very weak diffraction, single-crystal X-ray diffraction (SC-XRD) analysis could be performed after measurements at PETRA III beamline, DESY (Hamburg)^40^ (see the SI for crystallographic details). Cage **6a** crystallizes in the tetragonal space group *I*4/m. Within the *ab* plane, slightly rotated cubic cages are arranged in densely packed square arrays, which are stacked in an AB fashion along the *c-*axis (Figure 3a). Due to steric crowding, π–π interactions between the cube edges are prohibited, but crystal packing is maintained via multiple dispersion interactions between *t*-Bu groups and aromatic rings of adjacent cages (Figure S50). Thereby, the BDBA edges are twisted and rotated out of plane of the catechol units at the TBTQ vertices (Figure S50a,b). The excellent agreement between experimental powder X-ray diffraction (PXRD) for bulk samples of **6a** and a diffractogram calculated from SC-XRD data further demonstrates the robustness and integrity of the 3D crystal packing (Figure 3b). N_2_ sorption experiments at 77 K revealed a type I(b) isotherm, which is typical for materials in the intermediate regime between micro- and mesopores.^41^ Application of the Brunauer–Emmett–Teller (BET) theory resulted in a surface area (SA_BET_) of 2534 m^2^ g^–1^ (Figure 3c and Figure S21),^41^ which ranks **6a** among the most porous cages and the very rare literature reports with SA_BET_ > 2500 m^2^ g^–1^. The pore size distribution (PSD) was calculated from the absorption branch of the N_2_ isotherm with a MDFT carbon kernel for cylindrical pores (Figure 3d). The two narrow pores at 1.70 and 0.6 nm nicely correlate with the intrinsic cage pores and the connecting windows within the *ab* plane of the SC-XRD structure (Supplementary Video S1). The additional broader feature in the mesopore region between 2.1 and 3.0 nm is most presumably related to specific defects with one cage vacant in the lattice (Figure S51 and Supplementary Video S2).

### Stability Measurements

**Figure 4:**
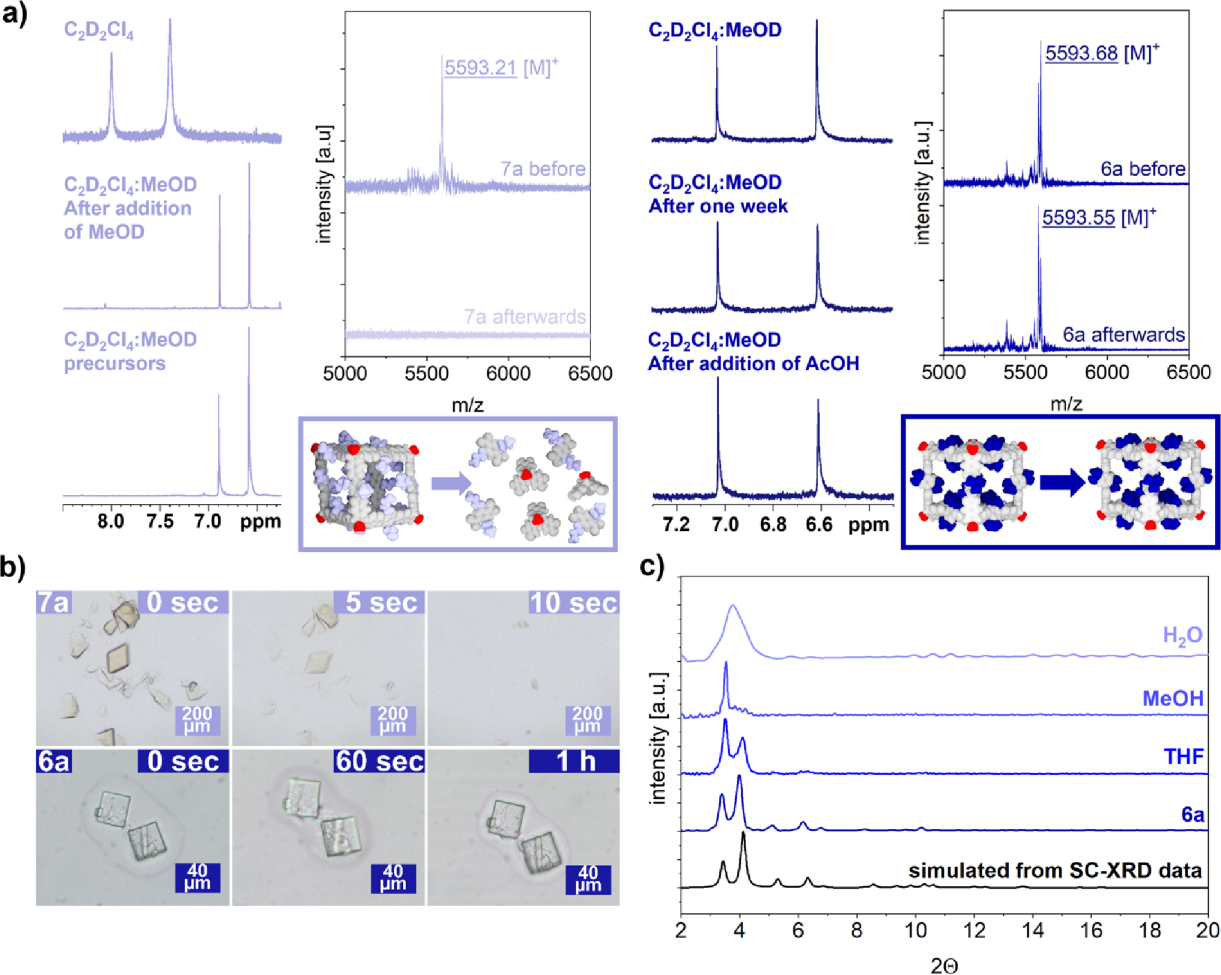
**|** a) Stability measurements in solution: ^1^H NMR and MALDI-TOF MS for **7a** (left) and **6a** (right) in mixtures containing protic MeOD. (b) Microscopic images of single crystals of **7a** (top) and **6a** (bottom) after suspension in MeOH. (c) PXRD data for crystalline samples of **6a** after consecutive washing steps with solvents with increasing polarity.

Already during synthesis and workup, we noted that the exchange of oxygen substituents at *t*-Bu-containing boronic acid **4** is kinetically suppressed under neutral conditions and even in nucleophilic solvents, e.g., MeOH. This remarkable observation contrasts with the notorious lability of nonhindered regular boronate ester cages. For instance, addition of D_3_COD to a C_2_D_2_Cl_4_ solution of reference cage **7a** initiates instantaneous and complete decomposition into precursors **5b** and **BDBA-***n***Bu** as evidenced by ^1^H NMR spectroscopy (Figure 4a). Furthermore, treatment of crystalline samples of **7a** (R^1^ = Me, R^2^ = *n*-Bu) with D_3_COD resulted in facile dissolution by decomposition and pure precursors are observed in ^1^H NMR spectra of the clear solutions (Figures S22 and S24). In stark contrast, **6a** remained intact in a 1:1 C_2_D_2_Cl_4_/D_3_COD mixture without any signs of decomposition even after 1 week (Figure 4a). Moreover, the exclusive detection of **6a** by MALDI-TOF MS unequivocally proved the structural integrity of the molecular cages in MeOH solution (Figure 4a and Figure S28). Even more impressively, these boronate ester cages are also resistant against moderate acidic conditions as no decomposition was observed by both ^1^H NMR and MALDI-TOF MS (Figure 4a,c) even 1 week after the addition of AcOH (2.91 mmol L^–1^ in 0.60 mL of cage solution). Ultimately, disassembly into molecular precursors was only initiated after consecutive addition of stronger TFA (2.18 mmol L^–1^) (Figures S27 and S28). For crystalline samples, the extraordinary stability of **6a** was demonstrated by gradual activation with solvents of increasing polarity (Figure S22). Suspension of the crystals for 24 h each in CHCl_3_, THF, MeOH, and even H_2_O did not result in any visible deterioration, and not even traces of the starting materials were identified in the filtrates after washing with deuterated solvents (Figure S23). PXRD after each washing step revealed that the underlying packing of the porous materials was maintained (Figure 4b),^42^ and subsequent MALDI-TOF MS confirmed the molecular ion peak at *m*/*z* = 5593.32. Impressively, no decomposition or cage fragments whatsoever were observed even after prolonged storage of the boronate ester cages in pure water.

For a direct visualization of the striking difference in cage stability, oily suspensions of single crystals for both **7a** and **6a** were subjected to a drop of MeOH and monitored over time with an optical microscope. Whereas labile cages **7a** were fully disassembled within 10 s, crystals of **6a** remained intact for several hours without any visible signs of degradation (Figure 4a). Finally, decomposition of these highly durable cage assemblies was only achieved after addition of a 5:1 mixture of MeOH/AcOH, which fully fragmented **6a** within 20 s (Figure S25).

To rationalize this surprisingly strong stabilizing effect of the *ortho*-*t*-Bu groups, we performed semiempiral PM6 calculations for two model reactions of catechol with either an *n*-Bu- or *t*-Bu-substituted boronic acid, respectively (see the SI for details). Based on these calculations, there are only minor differences in energy between the two isomers for all intermediates with trigonal coordination at boron. In case of borate intermediates, however, steric repulsion between the tetragonally coordinated boron center and the demanding *t*-Bu group resulted in a significant increase in energy (Figure S52). We therefore propose that any exchange of oxygen substituents in DBA **4** under neutral conditions is kinetically hindered through strong steric repulsion in the tetragonally coordinated intermediates. In summary, the introduction of *t*-Bu groups in the *ortho*-positions of the aromatic boronate ester linkages in cubic cages induced unprecedented hydrolytic stability under neutral and slightly acidic conditions for this notoriously sensitive dynamic coupling motif. Importantly, the disintegration of the cages under strongly acidic conditions suggests that these containers might be utilized for the stimuli-responsive release of encapsulated cargo or on-demand disassembly of porous structures in both solution and the solid state.

### Adsorption and on-Demand Release of β-Carotene

**Figure 5:**
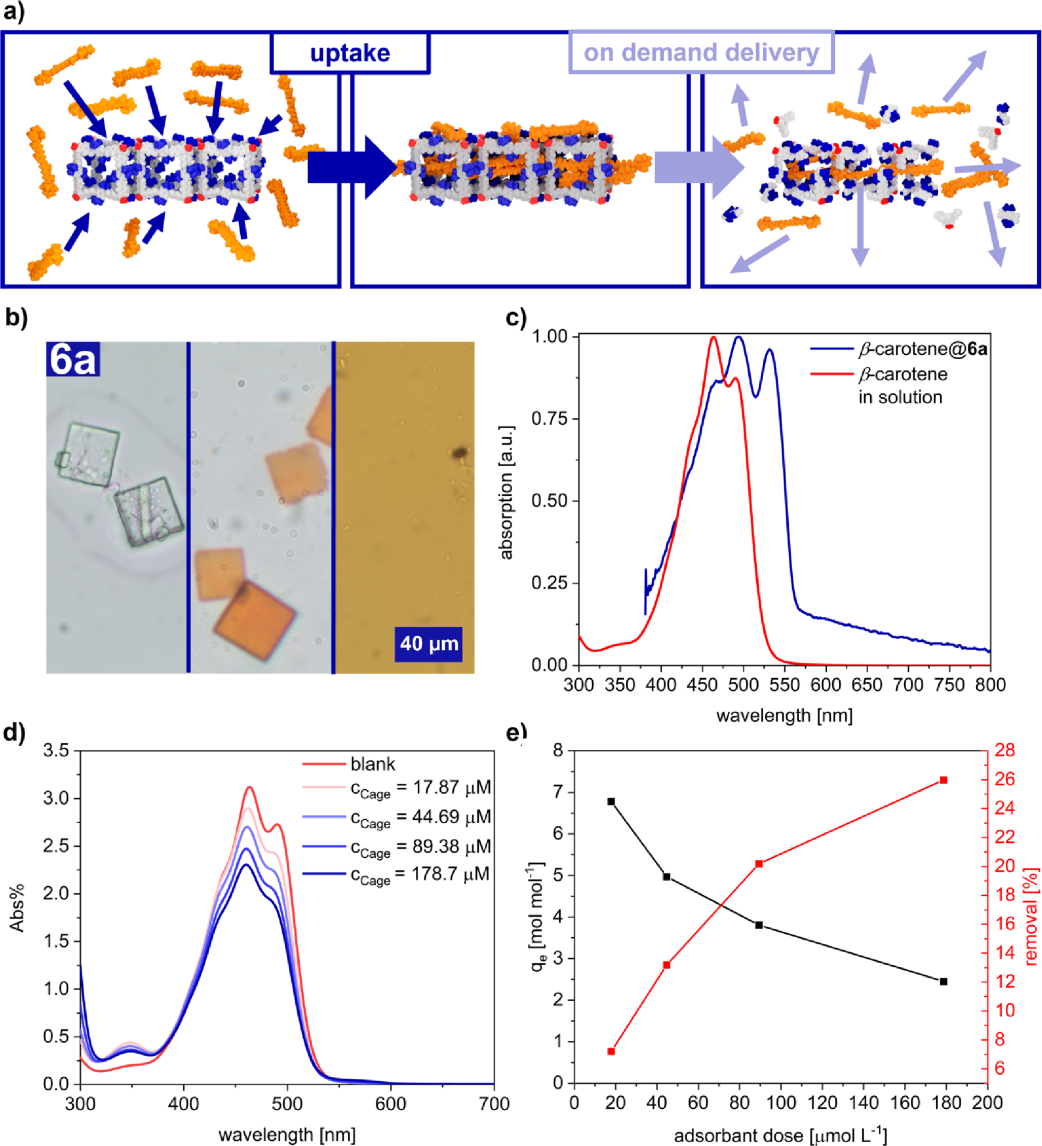
**|** (a) Uptake and stimuli-responsive release of β-carotene in porous **6a**. (b) Microscopic images of single crystals of **6a** before (left) and after (middle) loading with β-carotene and after acid-triggered release (right) of the encapsulated dyes. (c) UV/vis absorption of β-carotene in CH_2_Cl_2_ (red) and β-carotene⊂**6a** (blue). (d) UV/vis absorption of supernatant solutions after immersing varying amounts of **6a** (*c*(**6a**) = 17.9–179 μmol L^–1^) in a β-carotene stock solution. (e) Absorption capacity (black) and relative uptake of β-carotene by crystalline **6a**.

To date, crystalline cages have been predominantly investigated as microporous gas absorbents or hosts for rather small molecules.^43−45^ To demonstrate the unprecedented stability and to fully utilize the well-defined and spacious pore system in crystalline **6a**, we examined the absorption capacity and on-demand release of β-carotene as a model for large aromatic dye molecules.^46−48^ After immersing **6a** in a β-carotene solution in CH_2_Cl_2_, dye adsorption was obvious for the naked eye by a distinct tinting of the formerly colorless crystals (Figure 5b). Localized UV/vis absorption spectroscopy at the bulk crystals with a confocal microscope revealed the typical signature for β-carotene with a red-shifted aggregate band at 550 nm compared to monomeric β-carotene in CH_2_Cl_2_. According to literature, this bathochromic shift can be attributed to *J*-type aggregation of the elongated dyes within the mesopores (Figure 5c).^49,50^ To quantify the uptake, increasing amounts of solid **6a** (*c* = 17.9–179 μmol L^–1^) were added to a stock solution of β-carotene in CH_2_Cl_2_ (*c*_0_ = 196 μmol L^–1^). Via a freshly prepared calibration curve for the change in absorbance at λ_max_ (β-carotene) = 462 nm (Figure S33), the absorbed quantity was calculated from the remaining concentration of the supernatant solution (Figure 5d). Repeated experiments showed the reproducibility of this approach (Figure S35a). Whereas application of the Langmuir model, which is related to monolayer adsorption at homogeneous sites, was not feasible in this case, fitting of the experimental data (Figure 5e) with the empirical Freundlich model, which also account for multilayer adsorption at heterogeneous sites,^51^ revealed a maximum uptake of up to seven β-carotene molecules per cubic cage **6a** and an unfavorable adsorption process with an *n* value of 0.22 (Figure S32). We therefore assume that the initial absorption of β-carotene monomers in the pores is not favored due to the lack of extended π–π interactions with the cage backbone. Once a certain threshold is exceeded, however, pronounced aggregation of the dyes within the pores induces cooperative uptake at higher concentration. After loading, β-carotene⊂**6a** can be easily isolated by simple filtration and redispersion in fresh CH_2_Cl_2_ did not reveal any leakage of the encapsulated dye. However, the on-demand release of the encapsulated cargo was induced by the addition of a 2:1 CH_2_Cl_2_/AcOH mixture, as the instantaneous disappearance of the crystals and liberation of β-carotene into the solution was observed under the microscope (Figure 5b and Figure S30). This example impressively demonstrates the versatility of the sterically hindered *t*-Bu cages **6** for a variety of applications, which are impossible to realize with state-of-the-art boronate ester materials.

### Heterogeneous Water Oxidation Catalysis

**Figure 6:**
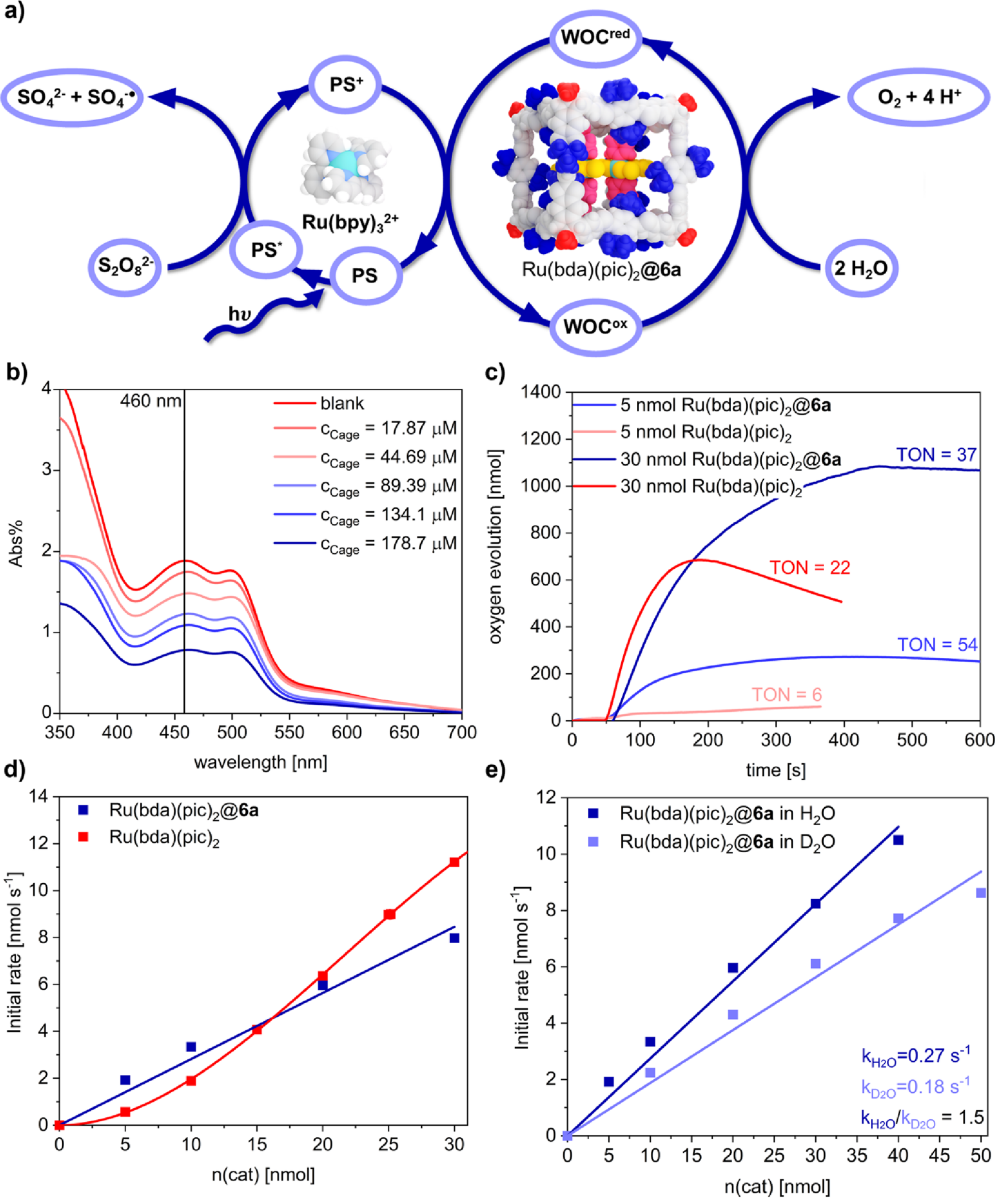
**|** (a) Photocatalytic water oxidation with cage-embedded Ru(bda) complexes. (b) UV/vis Absorption spectra for supernatant solutions after suspending varying amounts of crystalline **6a** (*c* = 17.87–178.8 μmol L^–1^) in a Ru(bda)(pic)_2_ stock solution in CD_3_CN (*c* = 1.00 mmol L^–1^). (c) Oxygen evolution after photocatalytic water oxidation with Ru(bda)(pic)_2_ and Ru(bda)⊂**6a** in 40:60 MeCN:H_2_O (pH 7.2 phosphate buffer), (d) initial rates of oxygen evolution for pristine Ru(bda)(pic)_2_ (red) and Ru(bda)⊂**6a** (blue), and (e) kinetic isotope effect studies for Ru(bda)⊂**6a**.

As a second example to demonstrate the durability of cage **6** and to highlight the unprecedented application of porous boronate ester materials in aqueous media, **6a** was used as a well-defined porous matrix for heterogeneous water oxidation catalysis. Ru(bda) complexes (bda = 2,2′-bipyridine-6,6′-dicarboxylate) have emerged as one of the most successful synthetic platforms to mimic the oxygen evolving complex in photosystem II (OEC-PSII) by performing the demanding four-electron oxidation half-reaction for water splitting. In recent years, we have presented a variety of highly efficient supramolecular Ru(bda)-based water oxidation catalysts (WOCs) by facilitating water networks within macrocycles^52,53^ or heterogeneous COF nanoparticles.^54^ Ru(bda)(pic)_2_ (pic = 4-picoline)^55^ is one of the simplest and best-studied mononuclear WOCs within the Ru(bda) family.^56^ However, dissociation of the axial ligands is assumed as the main degradation pathway under operating conditions, thus significantly limiting the performance and stability of this prototype WOC. As a benchmark, we measured light-driven water oxidation with Ru(bda)(pic)_2_ in phosphate-buffered 4:6 MeCN/H_2_O mixture at pH 7.2 following established procedures in our laboratories.^52,53^ These photocatalytic experiments were performed in a three-component system with varying amounts of homogeneous WOC, [Ru(bpy)_3_Cl_2_] as photosensitizer and Na_2_S_2_O_8_ as sacrificial oxidant (Figure 6a). Oxygen evolution was measured with a Clark electrode. In accordance with literature reports,^55^ the quadratic dependence of the initial rate on the WOC concentration indicated a bimolecular I2M (interaction of two metal-oxo species) mechanism and modest activity and stability was observed with turnover frequencies (TOFs) in the range of 0.11–0.37 s^–1^ and a maximum turnover number (TON) of 22. To quantify the absorption of Ru(bda)(pic)_2_ in crystalline **6a**, varying cage amounts (*c* = 17.9–179 μmol L^–1^) were suspended in a CH_3_CN solution of (Ru(bda)pic_2_ (*c* = 1.00 mmol L^–1^). The catalyst loading was determined by UV/vis absorption spectroscopy of the supernatant solution utilizing a calibration curve for the change in absorbance at λ_max_(Ru(bda)(pic)_2_) = 460 nm (Figure 6b, Figures S34 and S35, and Table S3). Fitting of the experimental data to the Freundlich model (Figure S38) revealed a maximum absorption of up to six monomeric complexes per cage, which was confirmed by repeated UV/vis measurements (Figure S39a) and NMR integration after complete decomposition of the cages under acidic conditions (Figure S40). For heterogeneous water oxidation, freshly prepared crystals of Ru(bda)⊂**6a** with varying WOC loading were filtrated, washed with MeCN, and suspended in a phosphate-buffered 4:6 CH_3_CN/H_2_O mixture. Photocatalytic oxygen evolution was measured under conditions similar to those for pristine Ru(bda)(pic)_2_. Intriguingly, the encapsulated WOC showed first-order kinetics with regard to the catalyst amount with a TOF of 0.27 s^–1^ while basically retaining the catalytic performance of the monomer (Figure 6d). Even more impressively, the immobilization of the molecular WOCs within the porous cages significantly increased the maximum TON to 54 (Figure 6c and Figures S41–S47). As a control experiment, we also determined the catalytic activity of Ru(bda)(pic)_2_ in the presence of cage precursors **4** and **5**, which basically replicated the results of the free catalyst (Figure S48) and therefore clearly demonstrated the cage effect of the heterogeneous cocrystals of cage and WOC. We attribute the higher turnover of this hybrid system to a reduced decomposition of the encapsulated WOCs during catalysis. Furthermore, the forced proximity of the mononuclear complexes within the confined pores induced a change in kinetics and significantly increased catalytic activity at very low concentrations. This change in reaction order is caused by either a mechanistic switch from I2M to WNA (water nucleophilic attack) or the confinement-induced formation of active dimers, which eliminates diffusion as the rate-limiting factor in the bimolecular I2M mechanism.^54,57,58^ Kinetic isotope effect (KIE) experiments in D_2_O and H_2_O revealed a ratio *k*_H2O_/*k*_D2O_ of 1.5 for Ru(bda)⊂**6a**, which is considerably higher as for pristine Ru(bda)(pic)_2_ (KIE = 0.94) but still at the border between primary and secondary KIE (Figure 6e). We therefore assume that most of the oxidations with encapsulated WOC still follow the I2M mechanism. However, a partial switch to WNA and slower proton-coupled electron transfer processes might explain the higher KIE.

## Conclusions

In a dynamic covalent approach, we synthesized cubic boronate ester cages **6** that exhibited unprecedented robustness and stability under ambient conditions. After installing sterically demanding *t*-Bu groups in *ortho*-positions of diboronic acid precursor **4**, exchange of oxygen substituents via tetragonal intermediates at the boron sites is kinetically suppressed under neutral conditions but only catalyzed in acidic media. For the first time, boronate ester cages **6** can be dissolved in protic solvents, e.g., MeOH, and tolerate slightly acidic conditions without any signs of degradation. As evidenced by PXRD and MS, crystalline samples of **6a** remain structurally intact and retain a well-defined microporous solid-state arrangement (SA_BET_ = 2534 m^2^ g^–1^) even after prolonged storage in MeOH or H_2_O. The unrivaled durability of these highly porous boronate ester materials was exemplified by adsorption of β-carotene and on-demand release from β-carotene⊂**6a** under strong acidic stimulus. Encapsulation of a molecular Ru catalyst in heterogeneous Ru(bda)pic_2_⊂**6a** was utilized for photocatalytic water oxidation with enhanced kinetics and stability compared to those of the homogeneous reference. Any such applications under ambient or even aqueous conditions have so far been unconceivable for notoriously labile state-of-the-art boronate ester materials. With prototypical organic nanocubes **6**, we established a novel design paradigm for the dynamic covalent construction of highly rigid and directional nanoarchitectures based on boronate esters. These bonds reversibly form under catalytic conditions but become very stable once the final structure is assembled. We envision that this concept of stabilization by steric shielding can be easily transferred to other systems and will strongly revive the field of dynamic covalent boronate ester materials for numerous applications.

## Supporting Information Available

Materials and chemicals; technical equipment and general procedures; synthesis and optimization; NMR spectroscopy; mass spectrometry; reaction control; powder X-ray diffraction; BET sorption measurements; stability experiments; dye adsorption and water oxidation catalysis; single-crystal X-ray diffraction; and semiempirical calculations (PDF)
Thin section rotation around the cage pore for the X-ray structure of **6a** (MP4)
Thin section rotation around the defect site for the X-ray structure of **6a** (MP4)
