# Supplementary material for: A Water-Stable Boronate Ester Cage
Source: J Am Chem Soc. 2024 Feb 7;146(8):5305–15. doi: 10.1021/jacs.3c12002 (PMC10910528; doi:10.1021/jacs.3c12002)
Supplement: Supplementary file 1 — ja3c12002_si_001.pdf [file ja3c12002_si_001.pdf]

Supporting Information

A Water Stable Boronate Ester Cage

Philipp H. Kirchner,[a,b] Louis Schramm,[a,b] Svetlana Ivanova,[a,b] Kazutaka Shoyama,[a,b] Frank Würthner[a,b] and Florian Beuerle*[,a,b,c]

[a] Julius-Maximilians-Universität Würzburg, Institut für Organische Chemie, Am Hubland, 97074 Würzburg (Germany)

[b] Julius-Maximilians-Universität Würzburg, Center for Nanosystems Chemistry (CNC), Theodor-Boveri-Weg, 97074 Würzburg (Germany)

[c] Institut für Organische Chemie, Universität Tübingen, Auf der Morgenstelle 18, 72076 Tübingen (Germany)

*E-mail: florian.beuerle@uni-tuebingen.de

Content

1	**Materials and Chemicals**	S2
2	**Technical Equipment and General Procedures**	S2
3	**Synthetic Scheme, Synthesis and Optimization**	S4
4	**NMR Spectroscopy**	S13
5	**Mass Spectrometry**	S19
6	**Reaction Control**	S21
7	**Powder X-ray Diffraction**	S22
8	**BET Sorption Measurements**	S23
9	**Stability Experiments**	S24
10	**Dye Adsorption and Water Oxidation Catalysis**	S29
11	**Single-Crystal X-ray Diffraction**	S42
12	**Semiempirical Calculations**	S47
13	**References**	S49

1 Materials and Chemicals

Chemicals: Commercially available chemicals were purchased from the following suppliers without further purification: ABCR, ALFA AESAR, ACROS ORGANICS, FISHER CHEMICALS, HONEYWELL, MERCK and SIGMA ALDRICH. Solvents were destilled before use. Dry solvents were dried with the solvent purification system "PureSolv MD 5" from INNOVATIVE TECHNOLOGY.

Column chromatography: Glass columns were individually packed with Silica gel (grain-size 463 µm, MERCK).

Flash chromatography: PuriFlash XS 420 from INTERCHIM, columns: PF30-SIHP-F0012, PF30-SIHP-F0025 and PF30-SIHP-F0040.

TLC-sheets: Silica gel 60 F_{254} TLC-aluminium foils (MERCK).

2 Technical Equipment and General Procedures

NMR spectroscopy: BRUKER AVANCE 400. Chemical shifts are given in ppm in relation to the particular internal standard (^{1}H-NMR: 7.26 ppm for $CDCl_3$, 6.00 ppm for $C_2D_2Cl_4$, 3.58 ppm for THF-d_8 and 3.31 ppm for MeOD; ^{13}C-NMR: 77.16 ppm for $CDCl_3$, 73.78 ppm for $C_2D_2Cl_4$, 67.21 ppm for THF-d_8 and 49.00 ppm for MeOD). Signal multiplicities are denoted as s (singlet), d (dublet), t (triplet) and m (multiplet). Processing of the raw data was performed with the program Topspin 4.1.4.

MALDI-TOF mass spectrometry: ultrafleXtreme BRUKER DALTONIC, matrix: DCTB (*trans*-2-(3-(4-*t*-Butylphenyl)-2-methyl-2-propenylidene)malononitrile) Solvent mixtures, mode and preparation technique are given individually at respective data sets.

ESI mass spectrometry: micOTOF focus BRUKER DALTONIC.

Melting points: OptiMelt automated melting point system MPA100 STANFORD RESEARCH SYSTEMS.

Elemental analyses: Elementar CHNS 932 analyzer LECO INSTRUMENTS.

Infrared spectroscopy: JASCO FT/IR-430. Samples were measured as solid state samples with ATR head.

Powder X-ray diffraction: BRUKER D8 DISCOVER. The measurements were carried out in reflection mode with Cu-K_{α}-radiation (λ = 1.5418 Å) and a position-sensitive detector (LynxEye) on a silicon wafer.

Single crystal X-ray diffraction: Single crystals suitable for X-ray diffraction were obtained from the reaction mixture after three to six days. Single crystals were picked from the mother liquor, quickly mounted onto nylon loops and immediately flash cooled in liquid nitrogen. Crystals were stored at cryogenic temperature in dry shippers, in which they were transported to macromolecular beamline P11,[1] PETRA III, DESY, Hamburg (Germany). Samples were mounted using the StäubliTX60L robotic arm.

BET-sorption and isotherms: Micro200 Surface area and pore size Analyzer from 3P instruments at 77K for nitrogen adsorption. Activation of porous material was performed at the preparation station of the Mirco200 Surface area and pore size analyzer at 20 °C and respective evacuation of the sample for 24 hours.

UV-Vis spectroscopy: Jasco V-670 UV-VIS-NIR Spectrometer

Photocatalytic water oxidation: A Oxygraph Plus Clark-electrode (HANSATECH INSTRUMENTS LTD.) was used for water oxidation catalysis. Irradiation of the samples was carried out with a Xenon lamp (NEWPORT, 150 W, calibrated to 100 mW cm^{-1}) and a UV-cutoff filter (400nm). Calibration of irradiation was performed with a PM 200 optical power meter with S121C sensor (THORLABS) and a CCs 200/M wide range spectrometer (THORLABS). Measurements were carried out with a stock solution (freshly prepared in the dark) of $[Ru(bpy)_3]Cl_2$ (c($[Ru(bpy)_3]Cl_2$) = 1.5 mM) as photosensitizer (PS) and the sacrificial electron acceptor $Na_2S_2O_8$ (c($Na_2S_2O_8$) = 37 mM) in an aqueous mixture (pH 7, phosphate buffered solution) with acetonitrile as co-solvent. A portion of this solution was mixed with catalyst solution in a transparent reaction chamber (water-cooled to 20 °C) while kept in the dark. Irradiation was started at 45 seconds to allow for sufficient equilibration. Calculations of TON were based on the maximum of evolved oxygen during the experiment divided by the amount of catalyst used. TOF was calculated by dividing the initial rate against catalyst amount. Initial rates were derived from the linear regression of the first 10 seconds of oxygen evolution after light irradiation.

3 Synthetic Scheme, Synthesis and Optimization

Synthetic Scheme

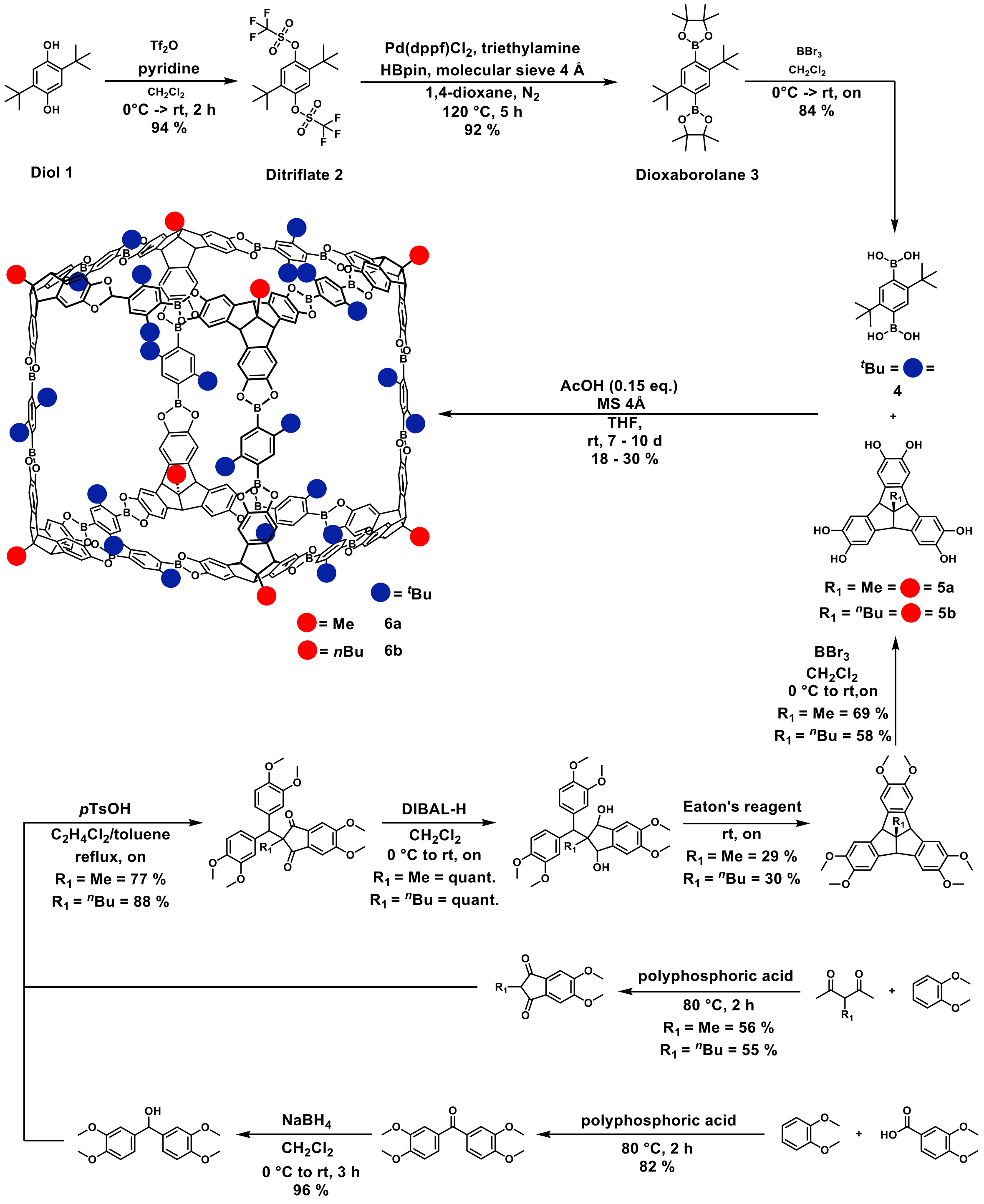

Figure S1. Overview for the synthesis of **5a**, **5b**, **4** and Cages **6a** and **6b**.

Synthesis of 5a and **5b**

5a and **5b** were synthesized according to literature.[2]

Synthesis of BDBA-nBu

BDBA-nBu was synthesized according to literature.[3]

Synthesis of Ru(bda)(pic)$_2$

Ru(bda)(pic)$_2$ was synthesized according to literature.[4]

Synthesis of 4

2,5-di-tert-butyl-1,4-phenylene bis(trifluoromethanesulfonate) 2

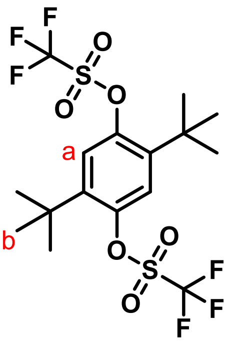

2

$C_{16}H_{20}F_6O_6S_2$

486.44 g/mol

2,5-Di-tert-butylhydroquinone **1** (730 mg, 3.28 mmol, 1.0 eq), pyridine (1.06 mL, 1.04 g, 13.12 mmol, 4.0 eq) and CH_2Cl_2 (4 mL) were put into a round bottom flask. The reaction mixture was cooled to 0 °C. Trifluoromethanesulfonic acid anhydride (1.32 mL, 2.20 g, 7.88 mmol, 2.4 eq) was added slowly. Afterwards the reaction mixture was stirred for two hours at 0 °C. Hydrochloric acid (1.5 M, 15 mL) was added. The mixture was extracted with CH_2Cl_2 (3 x 20 mL). The combined organic phase was washed with saturated $NaHCO_3$-solution (1 x 15 mL) and saturated NaCl-solution (1 x 15 mL). The organic phase was dried over Na_2SO_4 and the solvent was removed under reduced pressure. The crude product was purified by column filtration (*n*-hexane).

Yield: 1.51 g (3.08 mmol, 94%, Lit.[5]: 90%) as a colorless solid.

^{1}H-NMR (400 MHz, $CDCl_3$): δ = 7.43 (s, 3H, H_a), 1.41 (s, 18H, H_b) ppm.

^{13}C-NMR (101 MHz, $CDCl_3$): δ = 147.0, 141.8, 121.6 (q, $^4J_{CF}$ = 1.8 Hz), 118.5 (q, $^1J_{CF}$ = 320.1 Hz), 34.9, 30.0 ppm.

MS (MALDI-TOF, DCTB 1:3 in $CDCl_3$): *m/z* = 490.33 [M – F + Na]$^+$.

Melting point: 129.8 °C.

IR (ATR, RT): 2967.91, 2843.52, 1491.67, 1413.57, 1371.14, 1276.65, 1242.90, 1209.15, 1158.04, 1135.87, 1087.66, 1031.73, 892.88, 881.31, 838.88, 774.28, 739.57, 686.53, 607.47, 570.83, 523.58 cm^{-1}.

2,2'-(2,5-di-tert-butyl-1,4-phenylene)bis(4,4,5,5-tetramethyl-1,3,2-dioxaborolane) 3

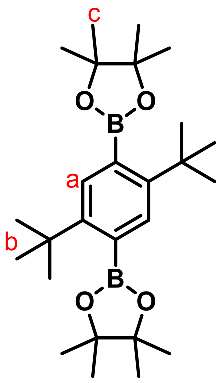

3

$C_{26}H_{44}B_2O_4$
442.25 g/mol

Under N_2-atmossphere ditriflate **2** (500 mg, 1.03 mmol, 1.0 eq), Pd(dppf)$_2$Cl$_2$ (56.41 mg, 77.09 µmol, 0.075 eq) and activated molecular sieves 4 Å (two balls) were put into a pressure tube and were put through three vacuum and N_2 flushing cycles. Dry dioxan (5 mL), triethylamine (1.08 mL, 780 mg, 7.71 mmol, 7.50eq) and HBpin (1.12 mL, 986.59 mg, 7.71 mmol, 7.5 eq) were added. The reaction mixture was heated to 120 °C and stirred overnight. After cooling to room temperature, the mixture was added to water (50 mL). After an extraction with CH_2Cl_2 (3 x 50 mL) the combined organic layers were dried over Na_2SO_4 and the solvent was removed under reduced pressure. The crude product was purified by flash chromatography (ethyl acetate : *n*-hexane 2 : 98).

Yield: 420 mg (949.7 µmol, 92%, Lit.[5]: 86%) as a colorless solid.

^{1}H-NMR (400 MHz, $CDCl_3$): δ = 7.43 (s, 3H, H_a), 1.37 (s, 24H, H_c), 1.37 (s, 18H, H_b) ppm.

^{13}C-NMR (101 MHz, $CDCl_3$): δ = 150.1, 130.4, 83.8, 35.7, 32.0, 24.9 ppm.

HRMS (ESI, positiv): *m/z* calc. for $C_{26}H_{44}B_2NaO_4$ $[M+Na]^+$ 465.33; exp. 465.33.

Melting point: 281.4 °C.

IR (ATR, RT): 2965.98, 2922.59, 2866.67, 2843.52, 1506.13, 1471.42, 1456.96, 1363.43, 1333.53, 1294.00, 1266.04, 1228.43, 1213.97, 1140.69, 1107.90, 1078.01, 1055.84, 1032.69, 1012.45, 962.31, 894.81, 856.24, 830.21, 806.10, 691.36, 673.04, 608.43, 578.54, 528.40 cm^{-1}.

Elemental analysis: calculated: $C_{26}H_{44}B_2O_4$: C, 70.61; H, 10.03. experimental: C, 70.65; H, 10.14.

(2,5-di-tert-butyl-1,4-phenylene)diboronic acid 4

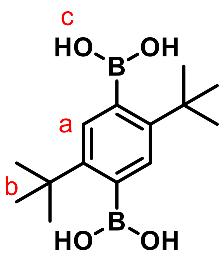

4

$C_{14}H_{24}B_2O_4$

277.96 g/mol

Under N_2-atmosphere dipinakolatoboronic ester **3** (588 mg, 904.4 µmol, 1.0 eq) was dissolved in dry CH_2Cl_2 and cooled to 0 °C. BBr_3 (257.19 µL, 679 mg, 2.71 mmol, 3.0 eq) was added dropwise over a period of 30 min. The reaction mixture was stirred at room temperature overnight. Water (60 mL) was added slowly, and the mixture was stirred roughly for 6 hours. Under reduced pressure CH_2Cl_2 was removed. The precipitated white solid was collected with a Whatman-filter and washed with *n*-hexane and CH_2Cl_2. The solid was dried over night before further use.

Yield: 312 mg (1.12 mmol, 84%) as a colorless solid.

^{1}H-NMR (400 MHz, THF-d_8): δ = 7.43 (s, 3H, H_a), 7.08 (s, 4H, H_c), 1.37 (s, 18H, H_b) ppm.

^{13}C-NMR (101 MHz, THF-d_8): δ = 148.0, 136.3, 129.1, 36.1, 32.3 ppm.

HRMS (ESI, positive): *m/z* calc. for $C_{14}H_{24}B_2NaO_4$ $[M+Na]^+$ 301.18; exp. 301.18.

Melting point: 291.3−346.3 °C (decomposition).

IR (ATR, RT): 3563.81, 3349.75, 3197.40, 2965.02, 2921.63, 2868.59, 2843.52, 1504.20, 1482.03, 1455.99, 1386.57, 1355.71, 1327.75, 1310.39, 1266.04, 1230.36, 1199.51, 1181.19, 1133.94, 1107.90, 1046.19, 1033.66, 991.23, 895.77, 829.24, 792.60, 702.93, 650.86, 595.90, 573.92, 520.67 cm^{-1}.

Elemental analysis: calculated: $C_{14}H_{24}B_2O_4$: C, 60.50; H, 8.70. experimental: C, 59.84; H, 8.80.

Synthesis of 6a

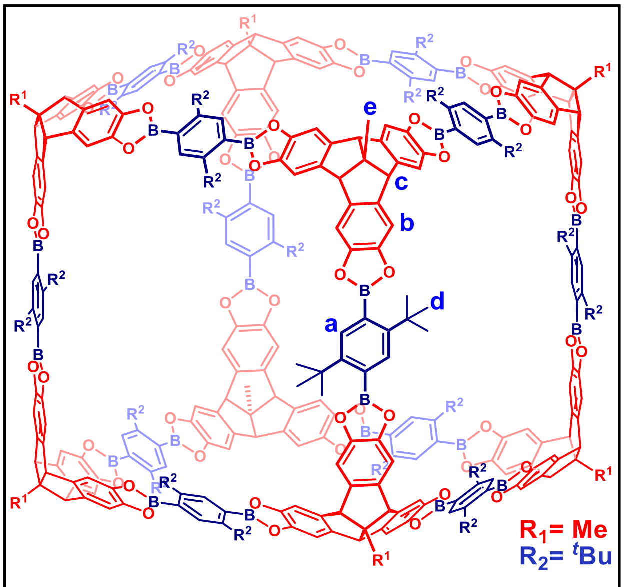

5a (10.00 mg, 25.62 µmol, 1.0 eq.) and **4** (10.68 mg, 38.42 µmol, 1.5 eq) were weighed at a ultra-fine scale to ensure optimal precision on the used equivalents. The two solids were combined in a 5 mL glass vial. Dry THF (1.0 mL) was added via a syringe. The resulting solution was subject to sonication to ensure complet dissolution. AcOH (0.220 µL, 230 µg, 3.85 µmol, 0.15 eq.) was added via Hamilton syringe directly into the solution. The reaction mixture was gently mixed. Freshly dried Molecular sieves 4Å (2 x balls) were added and the vial was closed with a plastic lid and parafilm. The reaction mixture was introduced to a vibration-free and temperature-controlled oven for 7-14 days (depending on the relative humidity on the setup day). The resulting crystals were filtered over Hirsch funnel equipped with a Whatman filter and washed with $CHCl_3$ (10 mL). The resulting crystals were washed with $CHCl_3$, THF and MeOH to ensure complete removal of fragments and acid traces from the product. The product was activated using *n*-pentane as activation medium. For stability studies, the crystals were further washed with copious amounts of $CHCl_3$, THF, MeOH and water (e.g. > 20 mL).

Yield: 25 mg (4.47 µmol, 30 %) as colorless crystals.

^{1}H-NMR (400 MHz, $C_2D_2Cl_4$, rt): δ = 7.56 (s, 24H, H_a), 7.41 (s, 48H, H_b), 4.55 (s, 24H, H_c), 1.29 (s, 216H, H_d), 1.25 (s, 24H, H_e) ppm.

MS (MALDI-TOF,positive, DCTB 1:1 in THF/$CHCl_3$) *m/z* = 5593.57 $[M]^+$ (theoretical: 5594.61$[M^+]$).

IR (ATR) = $\tilde{\nu}$ = 515.865, 622.895, 648.929, 697.141, 787.779, 817.67, 849.49, 891.916, 921.807, 1062.59, 1115.62, 1148.4, 1223.61, 1254.47, 1292.07, 1340.28, 1365.35, 1466.6, 2869.56, 2955.38 cm^{-1}.

Melting point: > 300 °C

Elemental analysis: $C_{352}H_{336}B_{24}O_{48} \cdot 57\ H_2O$ calculated: C: 63.86, H: 6.85, B: 3.92, O: 25.37 found: C: 63.87, H: 6.43.

Synthesis of 6b

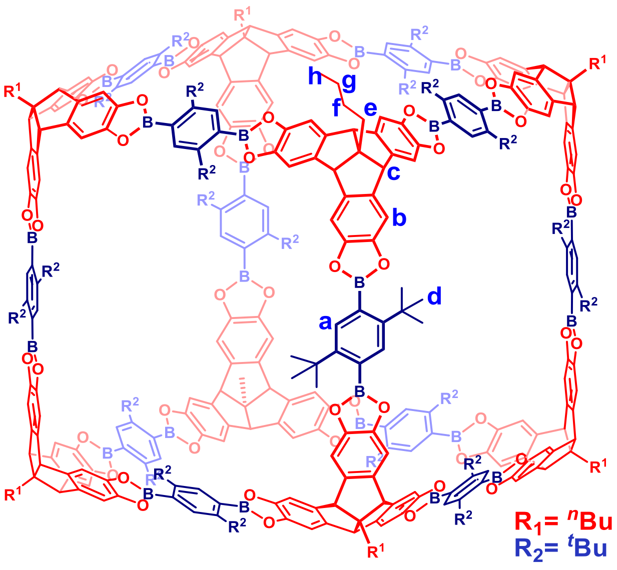

6b
$C_{376}H_{384}B_{24}O_{48}$
5930.60 g/mol

Cage **6b** was synthesized according to the general procedure described for the synthesis of **6a**. **5b** (10.00 mg, 25.62 µmol, 1.0 eq.) and **4** (10.68 mg, 38.42 µmol, 1.5 eq) were dissolved in dry THF (1.0 mL). AcOH (0.220 µL, 230 µg, 3.85 µmol, 0.15 eq.) was added and the reaction mixture gently mixed. Molecular sieves 4Å (2 x balls) were added and the reaction mixture was let stand for 7-14 days. The solvent was evaporated and the resulting solid was resolved in tetrachloroethane-d^2 at 80 °C for NMR-measurements.

Yield: 35 mg (5.90 µmol, 23 %) as a purple powder.

^{1}H-NMR (400 MHz, $C_2D_2Cl_4$, rt): δ = 7.56 (s, 24H, H_a), 7.41 (s, 48H, H_b), 4.65 (s, 24H, H_c), 2.04 (m, 16H, H_e), 1.39 (m, 32H, $H_{f,g}$), 1.30 (s, 216H, H_d), 0.96 (m, 24H, H_h) ppm.

DOSY-NMR (400 MHz, $C_2D_2Cl_4$, rt) d = 3.36 nm.

MS (MALDI-TOF, DCTB 1:1 in THF/$CHCl_3$) *m/z* = *m/z* = 5929.87 $[M]^+$.
(theoretical: 5930.61$[M]^+$])

Elemental analysis: $C_{376}H_{384}B_{24}O_{48}$ · 22 H_2O calculated: C: 71.38, H: 6.82, B: 4.10, O: 17.70 found: C: 71.40, H: 6.15.

Table S1. Optimization of reaction conditions for the synthesis of **6a** and **6b**.

sample	desired cage	conditions	result	Yield
**1**	**6b**	THF, MS 4 Å, TFA (3.00 eq)	precipitation of cage	NMR exp.
**2**	**6b**	THF, MS 4 Å, TFA (1.00 eq)	precipitation of cage	NMR exp.
**3**	**6a**	THF, MS 4 Å, TFA (1.00 eq)	small crystals	not isolated Crystallization exp.
**4**	**6a**	THF, MS 4 Å, TFA (3 x 1.00 eq)	small crystals	not isolated Crystallization exp.
**5**	**6a**	THF, MS 4 Å, KOH (3.00 eq)	precipitation of fragments	not isolated NMR exp.
**6**	**6a**	THF, MS 4 Å, KOH (0.2 eq)	precipitation of fragments	not isolated NMR exp.
**7**	**6a**	THF, MS 4 Å, TFA (10 eq)	crystals, purification issues	Not isolated due to TFA
**8**	**6a**	THF, MS 4 Å, TFA (20 eq)	crystals, purification issues	Not isolated due to TFA
**9**	**6b**	THF, MS 4 Å, TFA (1.00 eq)	cage formation after 7 d	not isolated NMR exp.
**10**	**6b**	THF, MS 4 Å, TFA (4.50 eq)	cage formation after 10 d	not isolated NMR exp.
**11**	**6a**	acetone, MS 4 Å, TFA (1.00 eq)	precipitation	fragment mixture
**12**	**6a**	DMSO, MS 4 Å, TFA (1.00 eq)	no reaction after 10 d	0%
**13**	**6a**	THF, MS 4 Å, TFA (0.50 eq)	small crystals	Crystallization exp

**14**	**6a**	THF, MS 4 Å, AcOH (1.00 eq)	slow cage formation	NMR
**15**	**6a**	acetone, MS 4 Å, TFA (0.50 eq)	fast reaction, precipitation	fragment mixture
**16**	**6b**	THF, MS 4 Å, TFA (0.50 eq) different preparations	different techniques show no difference in cage formation	1% - 3%
**17**	**6b**	THF, MS 4 Å, TFA (1.00 eq) slow evaporation of solvent	Crystals, purification issues	0%
**18**	**6b**	THF, MS 4 Å, AcOH (0.70 eq)	cage formation	10%
**19**	**6b**	THF, MS 4 Å, TFA (0.05 eq)	cage formation	5%
**20**	**6a**	THF, MS 4 Å, AcOH (0.15 eq)	crystals after 21 d	18%
**21**	**6a**	THF, MS 4 Å, AcOH (0.15 eq)	crystals after 21 d	30%
**22**	**6b**	THF/$CDCl_3$, MS 4 Å, AcOH (0.15 eq)	precipitation of cage amorphous	12%

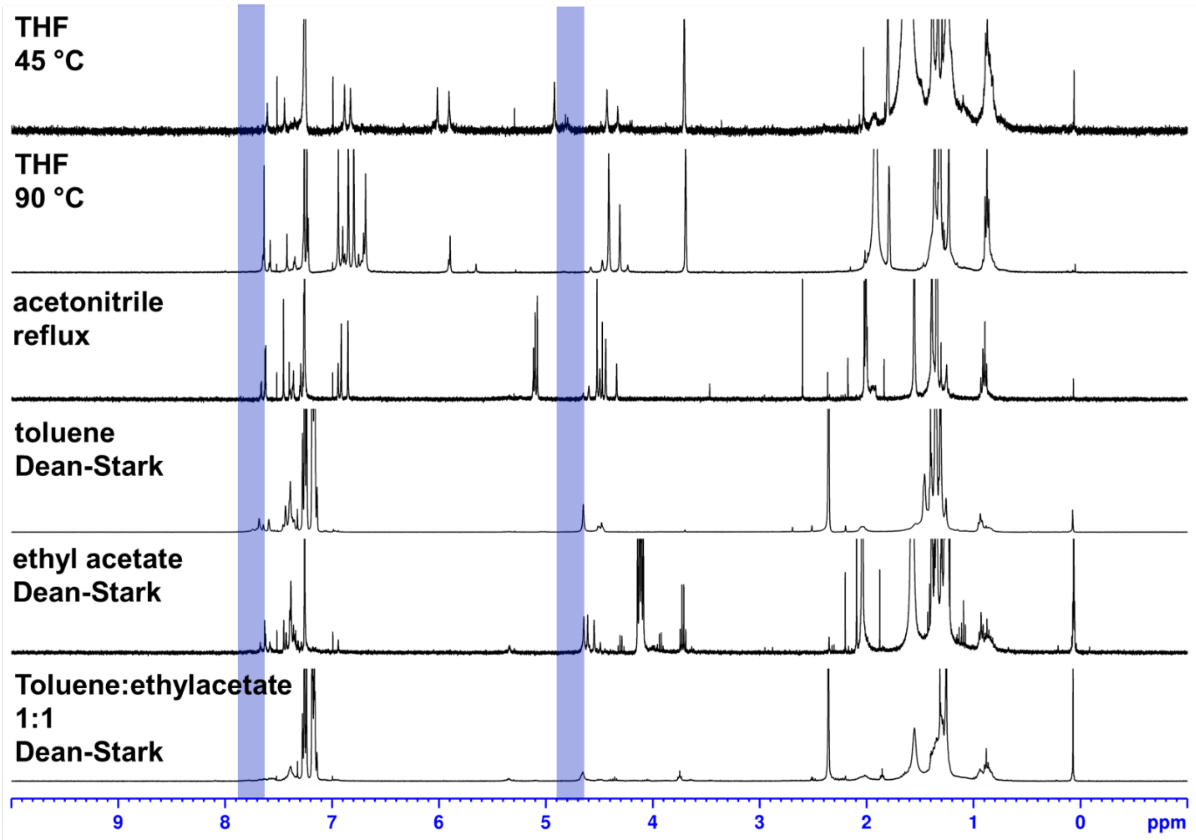

Figure S2. ^{1}H-NMR spectra for the screening of various reaction conditions for the synthesis of Cage **6b** (the respective areas were signals for **6b** should occur are marked in blue).

4 NMR spectroscopy

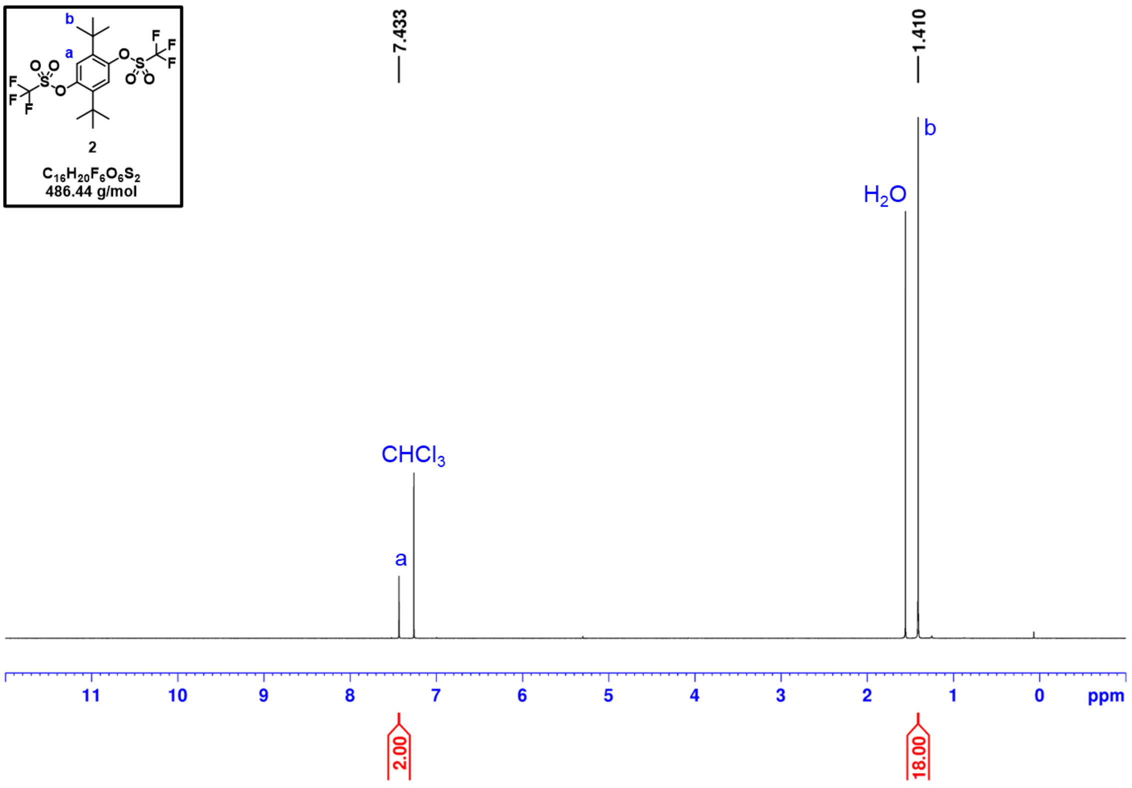

Figure S3. ^{1}H-NMR spectrum (400 MHz, $CDCl_3$, rt) of compound **2**.

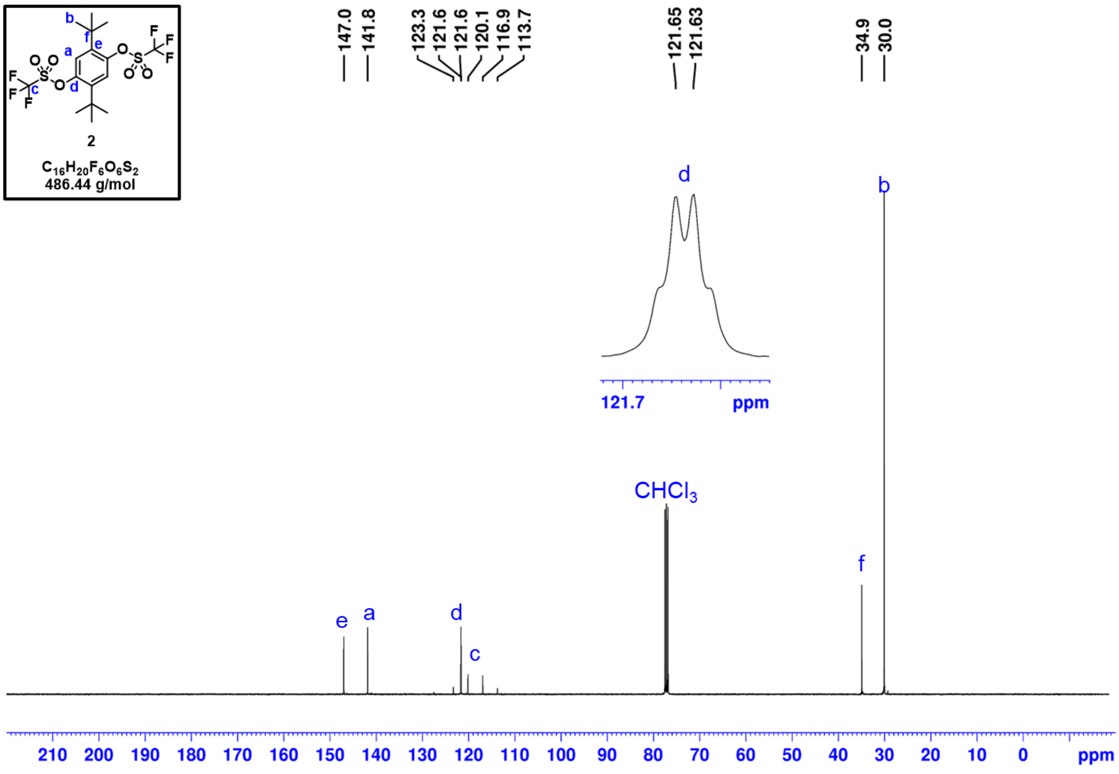

Figure S4. {^{1}H}^{13}C-NMR-spectrum (101 MHz, $CDCl_3$, rt) of compound **2**.

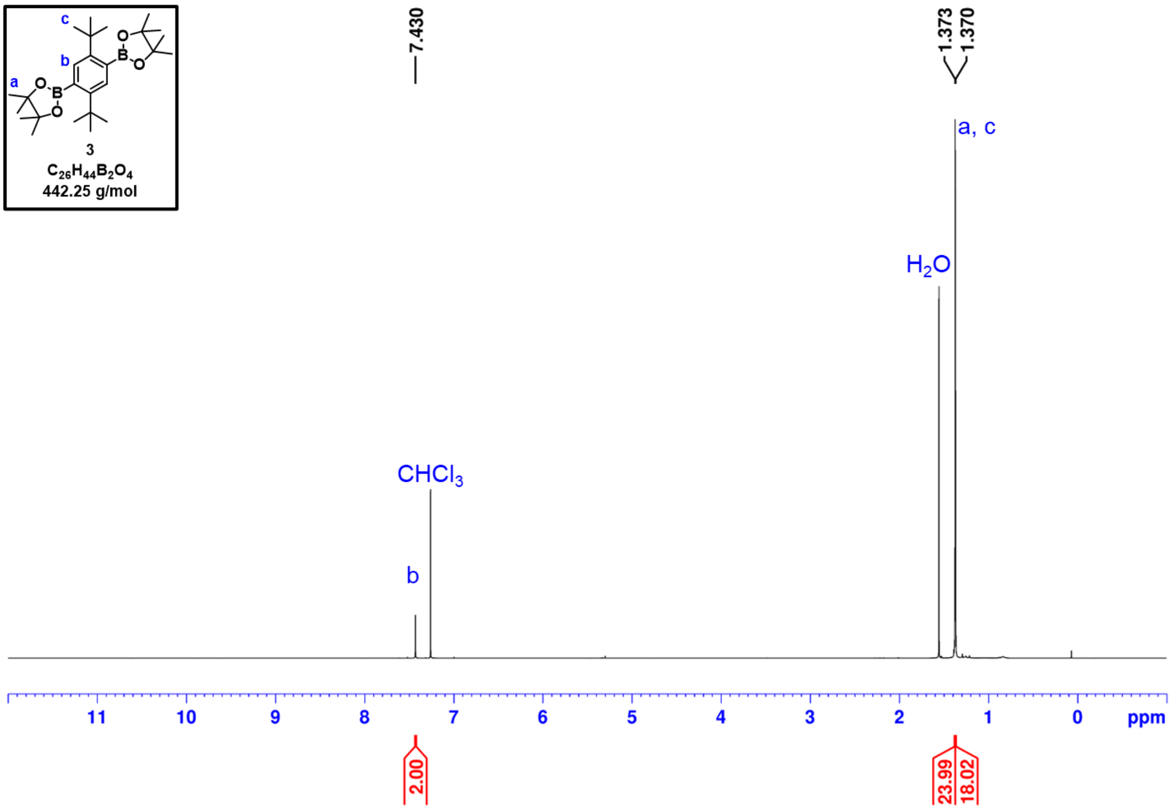

Figure S5. ^{1}H-NMR spectrum (400 MHz, $CDCl_3$, rt) of compound **3**.

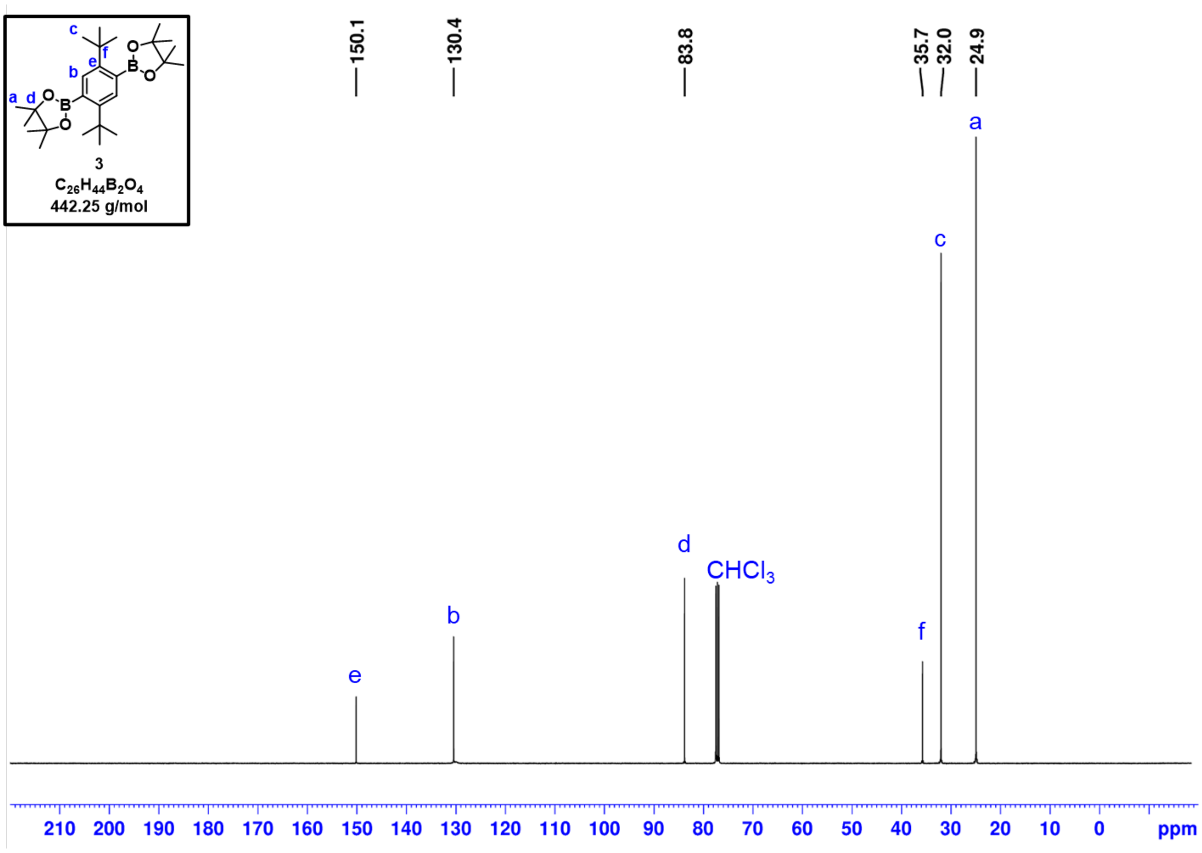

Figure S6. {^{1}H}^{13}C-NMR-spectrum (101 MHz, $CDCl_3$, rt) of compound **3**.

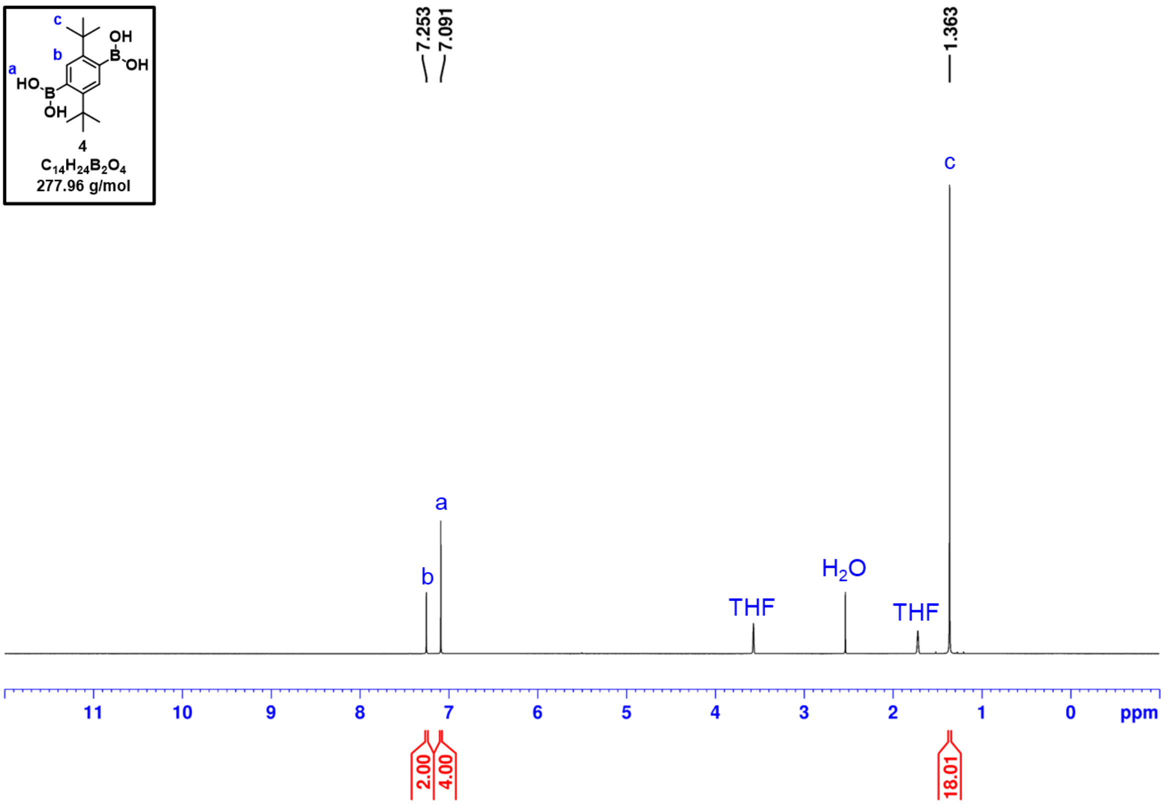

Figure S7. ^{1}H-NMR spectrum (400 MHz, THF-d8, rt) of **4**.

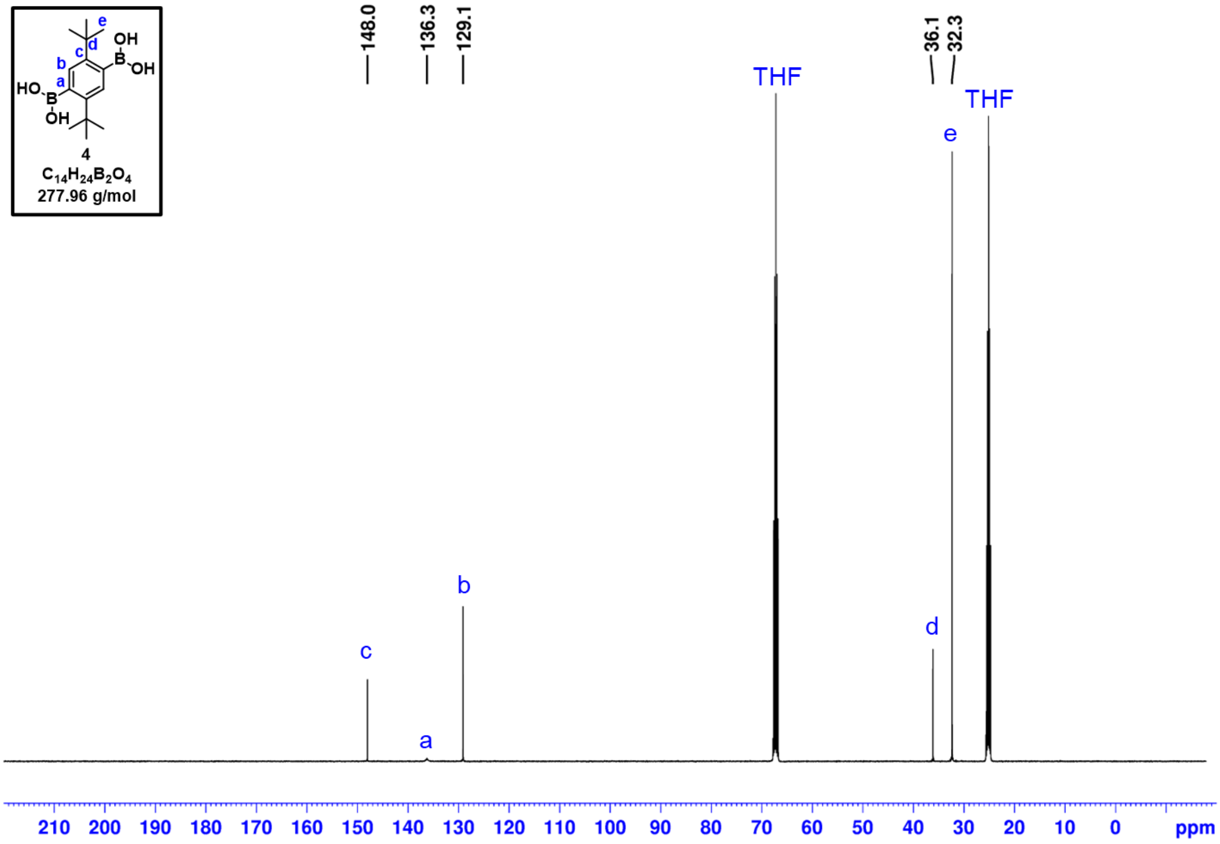

Figure S8. {^{1}H}^{13}C-NMR-spectrum (101 MHz, THF-d8, rt) of **4**.

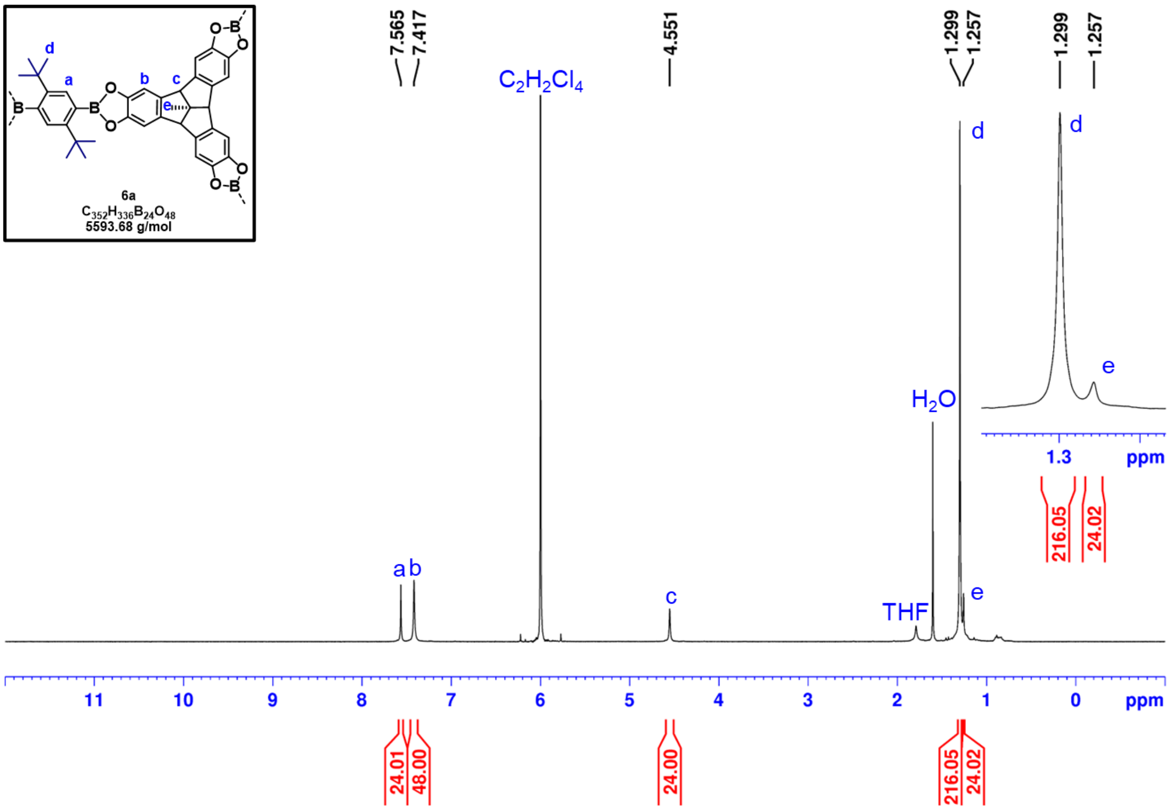

Figure S9. ^{1}H-NMR-spectra (400 MHz, $C_2D_2Cl_4$, rt) of **6a**.

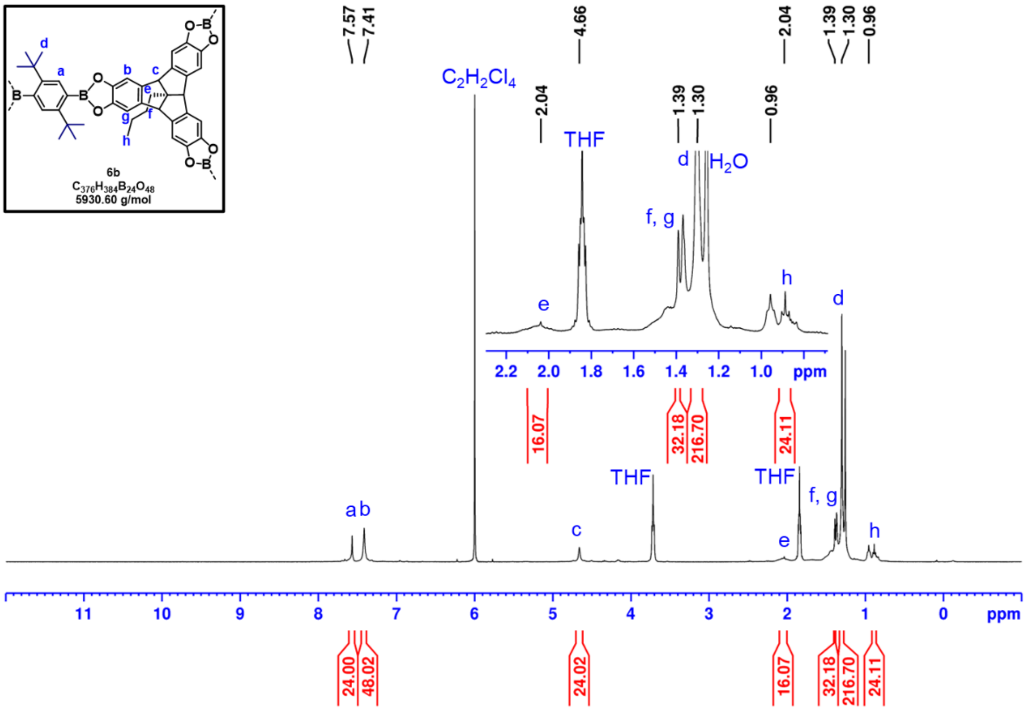

Figure S10. ^{1}H-NMR-spectra (400 MHz, $C_2D_2Cl_4$, rt) of **6b**.

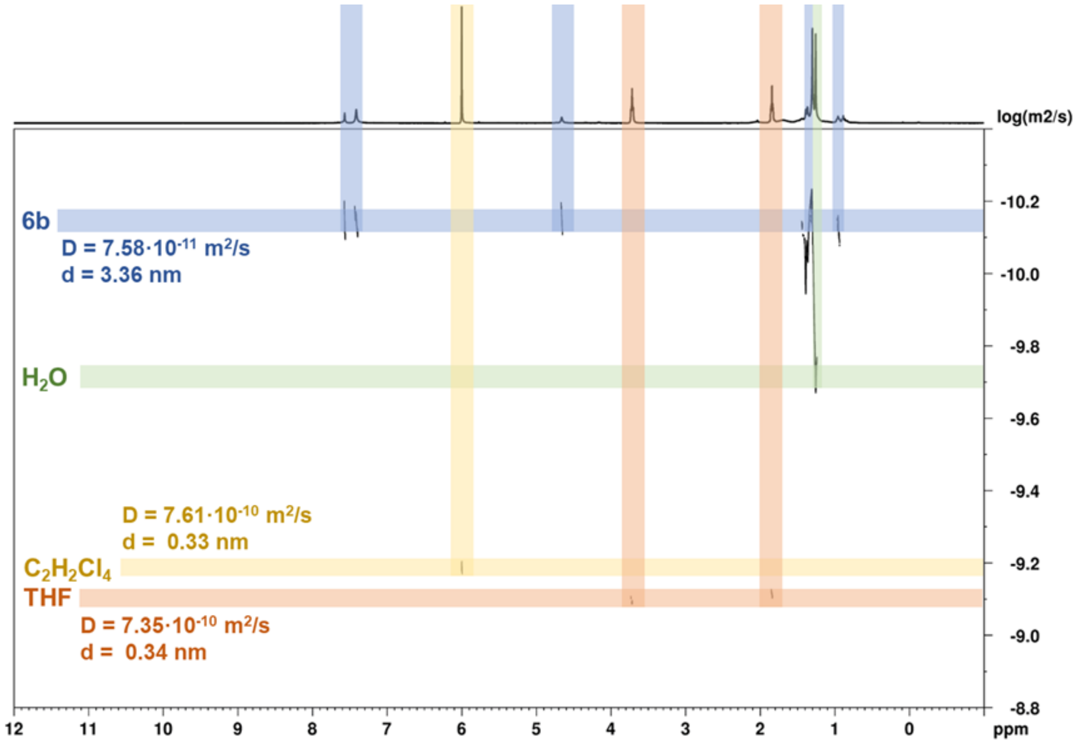

Figure S11. DOSY-NMR-spectra (400 MHz, $C_2D_2Cl_4$, rt, DSTE) of **6b**.

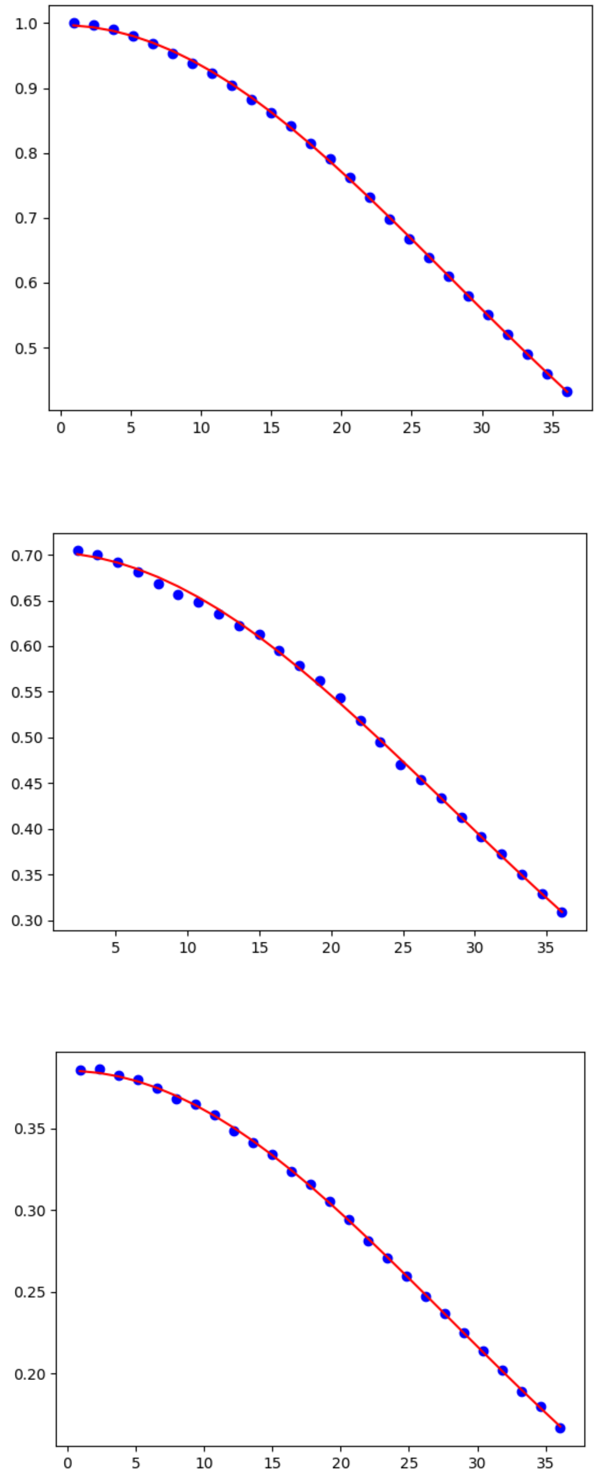

Figure S12. Fitting curves of DOSY-NMR-spectra (400 MHz, $C_2D_2Cl_4$, rt, DSTE) of **6b**.

5 Mass spectrometry

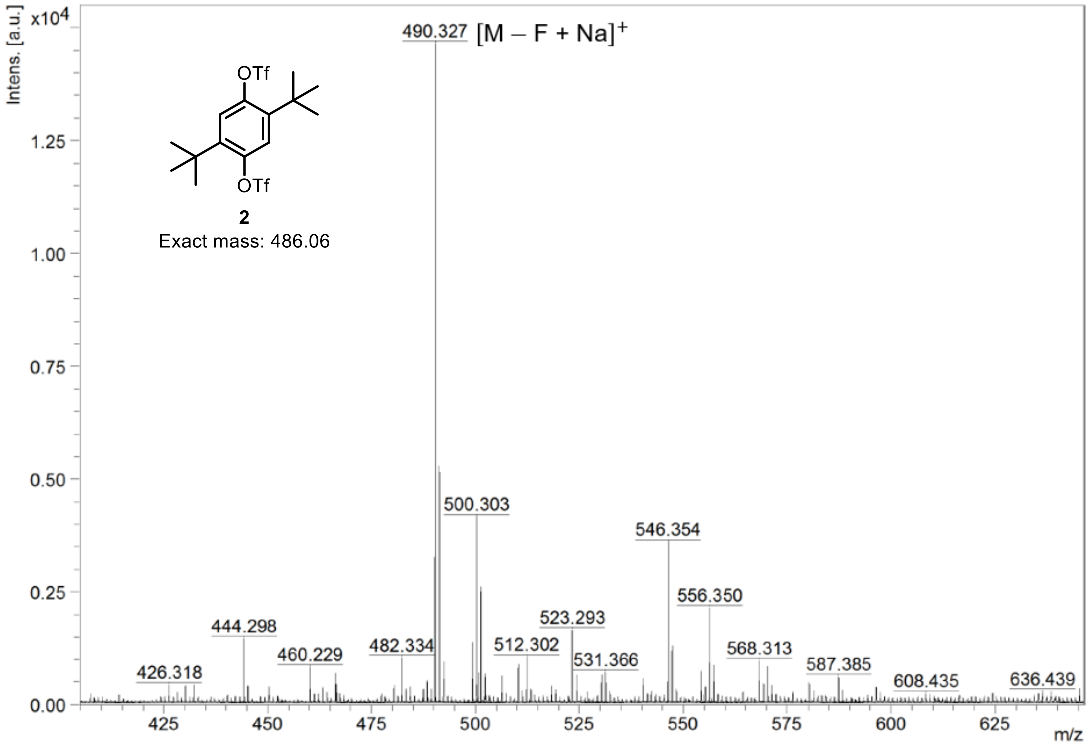

Figure S13. MALDI-TOF spectra ($CDCl_3$, DCTB, mode: positive) of compound **2**.

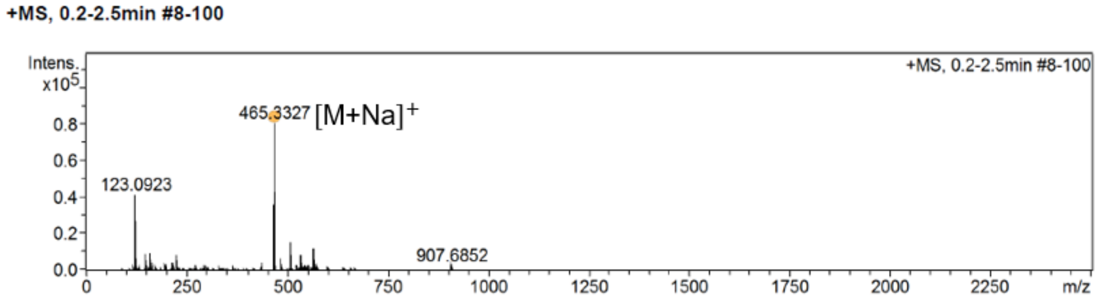

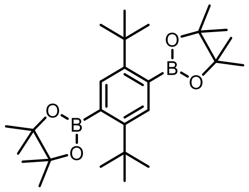

3
Exact mass: 442.30

Figure S14. ESI Mass-spectra (0.7 bar, 200°C, 5.0 L/min, mode: positive) of compound **3**.

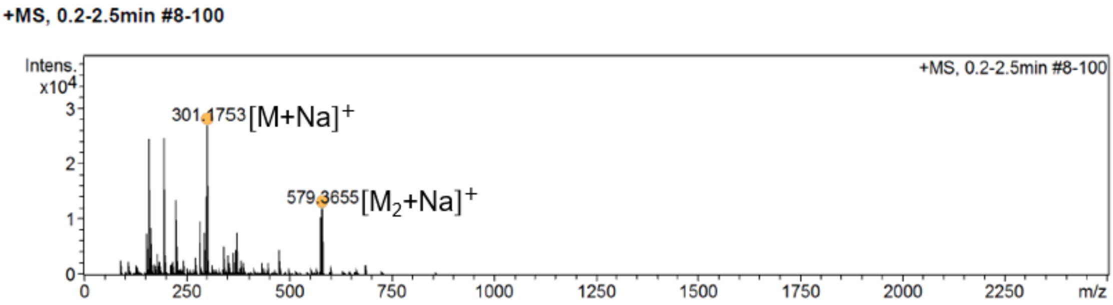

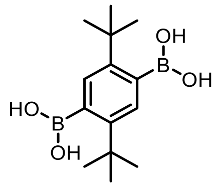

4
Exact mass: 278.20

Figure S15. ESI Mass-spectra (0.7 bar, 200°C, 5.0 L/min, mode: positive) of **4**.

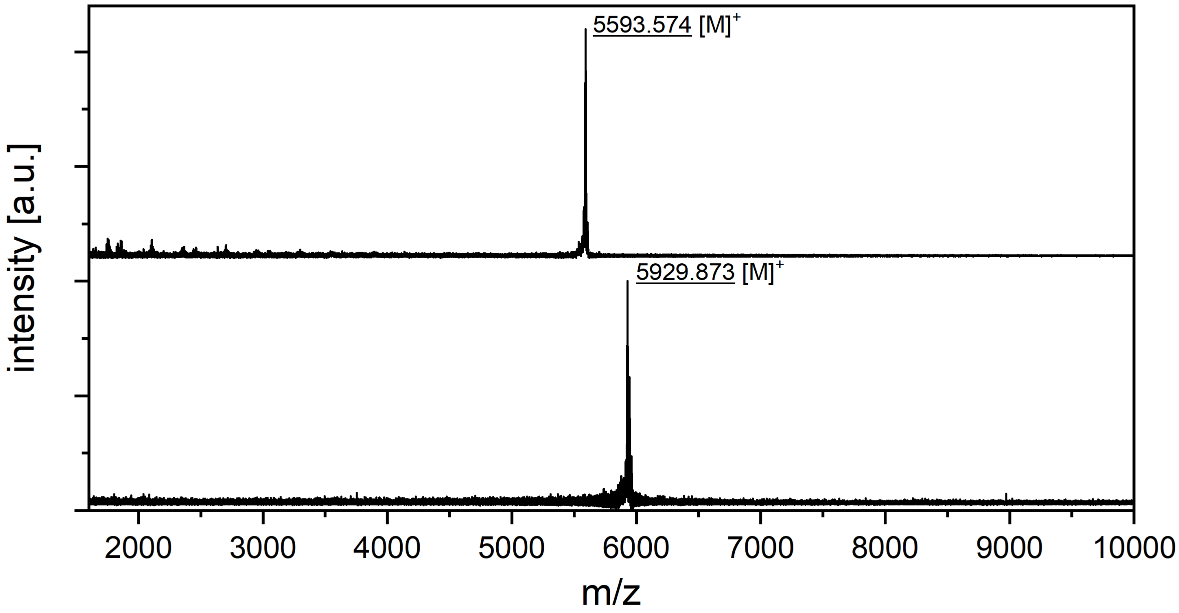

Figure S16. MALDI-TOF spectra ($CDCl_3$/THF, DCTB, mode: positive) of **6a** and **6b**.

6 Reaction Control

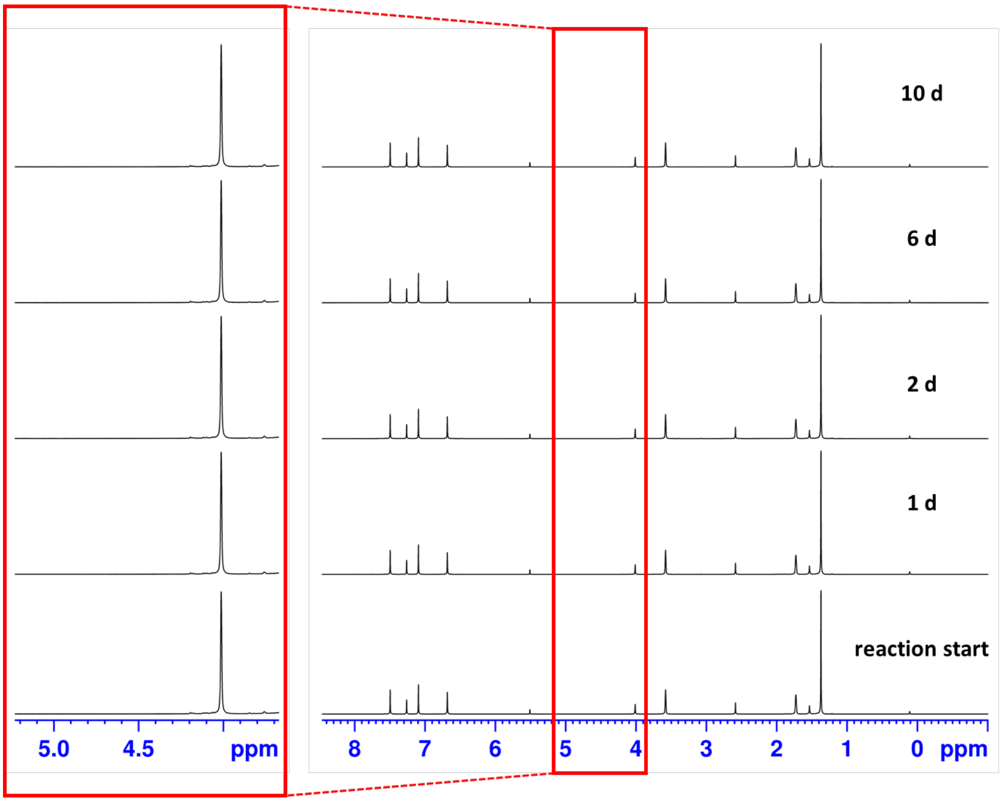

Figure S17. Reaction control via ^{1}H-NMR-spectroscopy (400 MHz, THF-d_8, rt) for the reaction conditions without acid addition with a zoom of the bridgehead region.

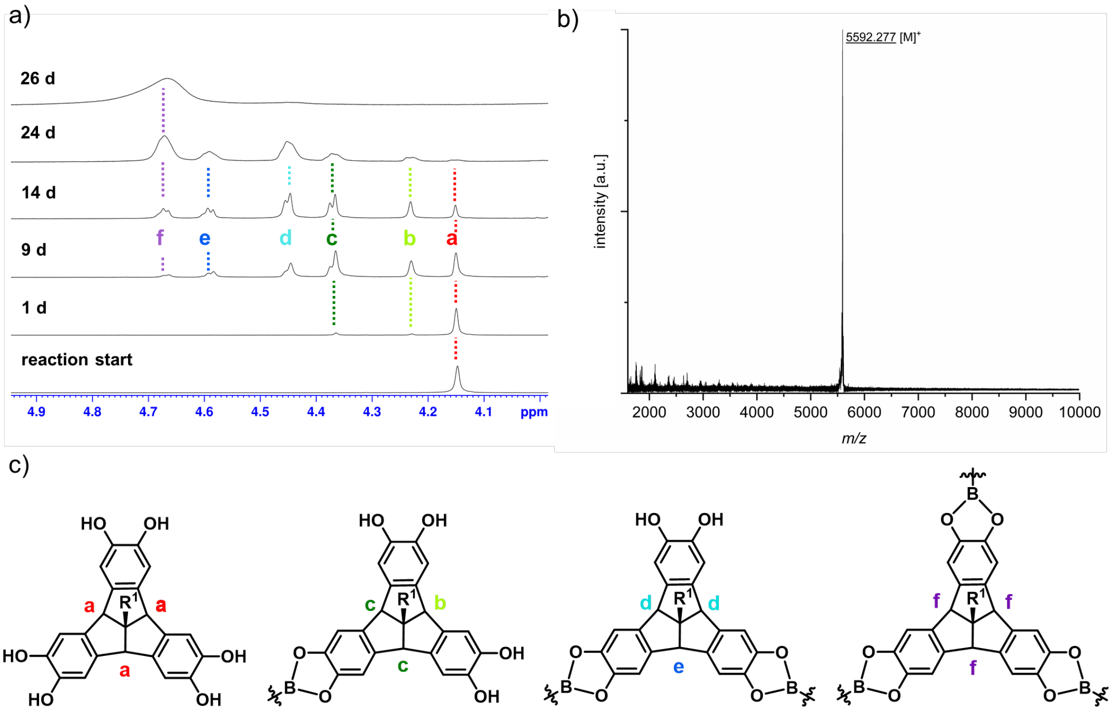

Figure S18. a) Reaction control via ^{1}H-NMR-spectroscopy (400 MHz, THF-d_8, rt) for the reaction conditions with acid addition (zoom of the bridgehead region), b) MALDI spectrum after 26 days and c) assignment of the signals.

7 Powder X-ray Diffraction

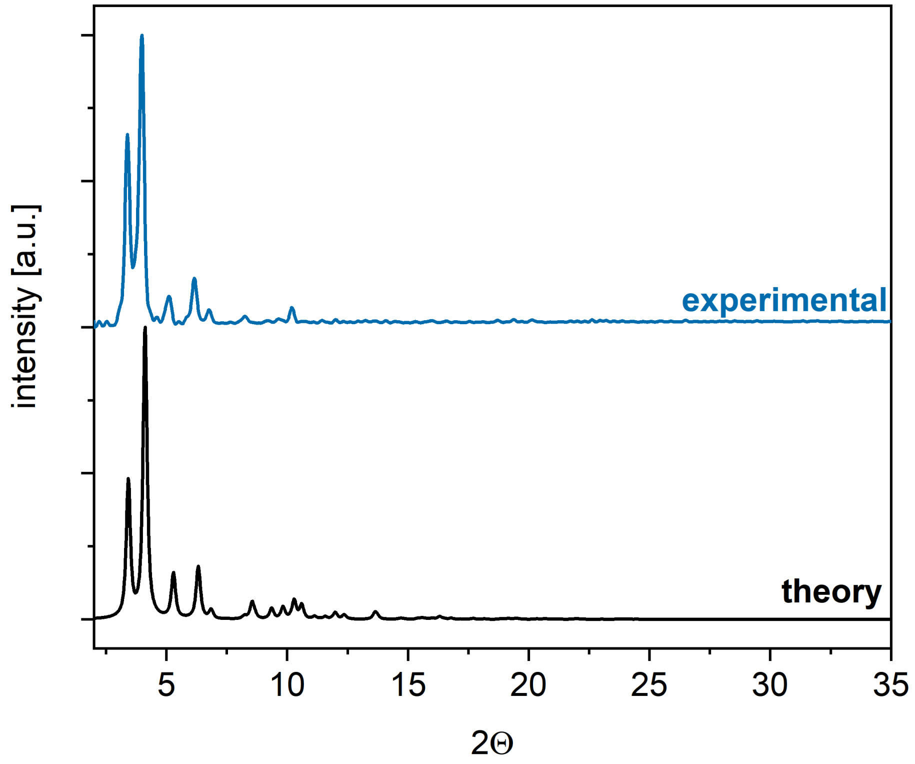

Figure S19. Experimental and theoretical (from SC-XRD) PXRD diffractogram (2Θ = 2°-40°) of **6a**.

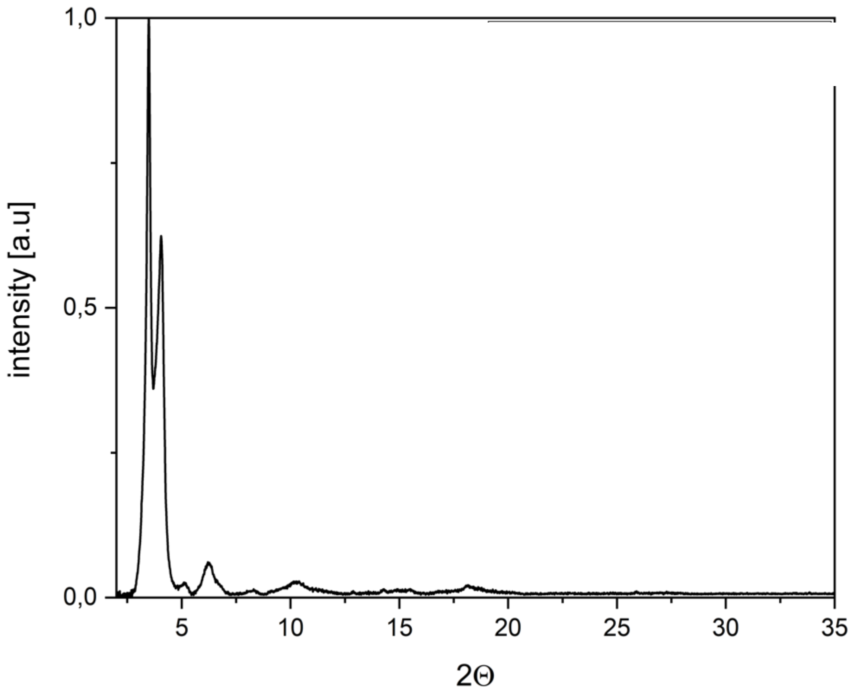

Figure S20. experimental PXRD diffractogram (2Θ = 2°-40°) of **6a** after washing steps of the stability experiments.

8 BET Sorption Experiments

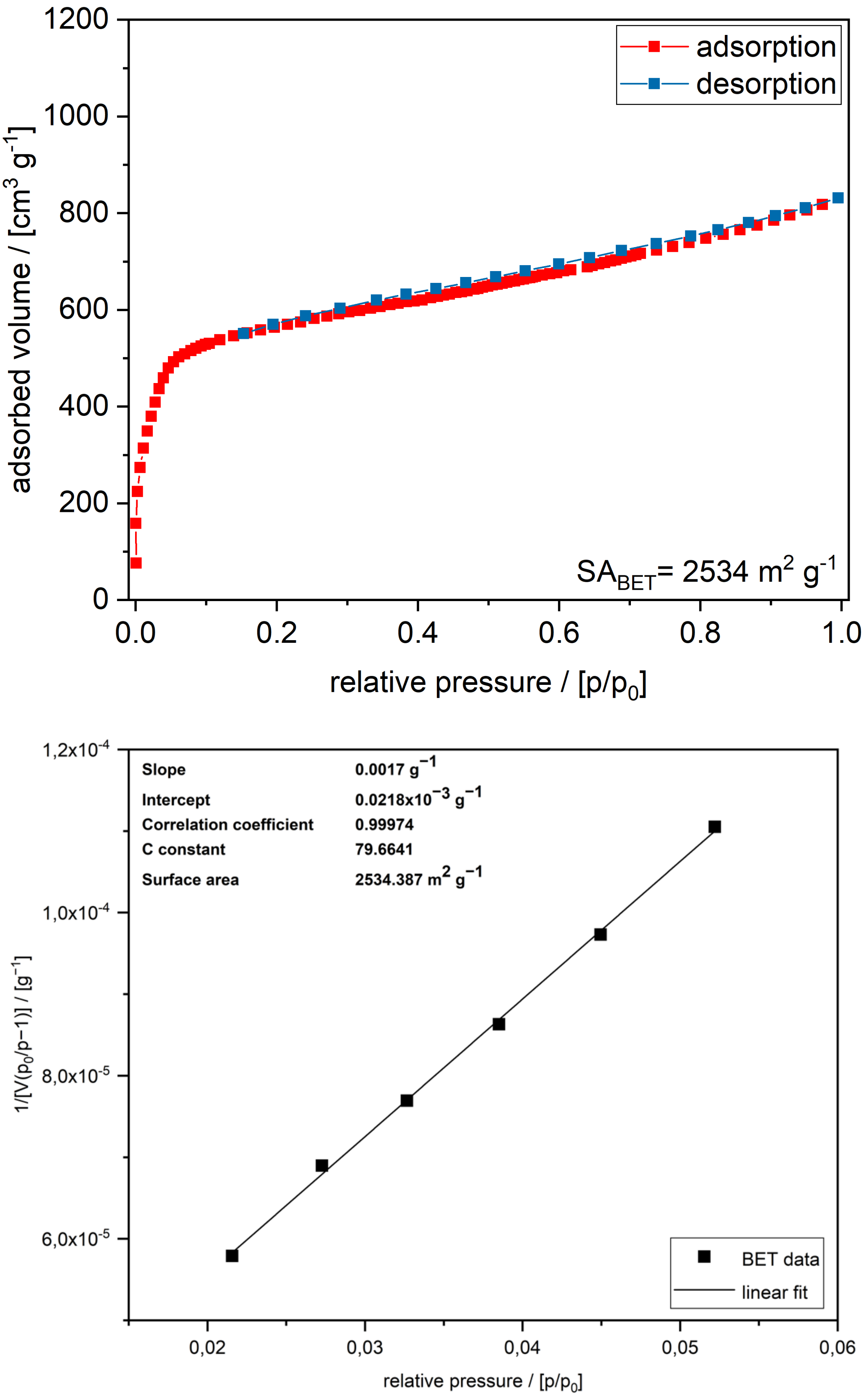

Figure S21. Micropore analysis of **6a** at 77 K with Nitrogen as adsorbed gas.

9 Stability Experiments

The stability experiments for the bulk material were carried out in the following procedure:

The crystals obtained directly from the reaction solution were separated by filtration over Hirsch funnel equipped with a Whatman filter. The crystals were washed thoroughly with $CHCl_3$, THF, MeOH and water. Afterwards the bulk material was stored in pentane for four days with daily exchange of the projecting pentane solution.

For PXRD data acquisition the crystals were stored in first pentane then THF, MeOH and water. In order to remove the solvent before measurement in the case of pentane, THF and MeOH slow and careful evaporation was suffient. For water solvent exchange was necessary to facilitate the removal of residual water in the pores and to get a dry material (THF then pentane).

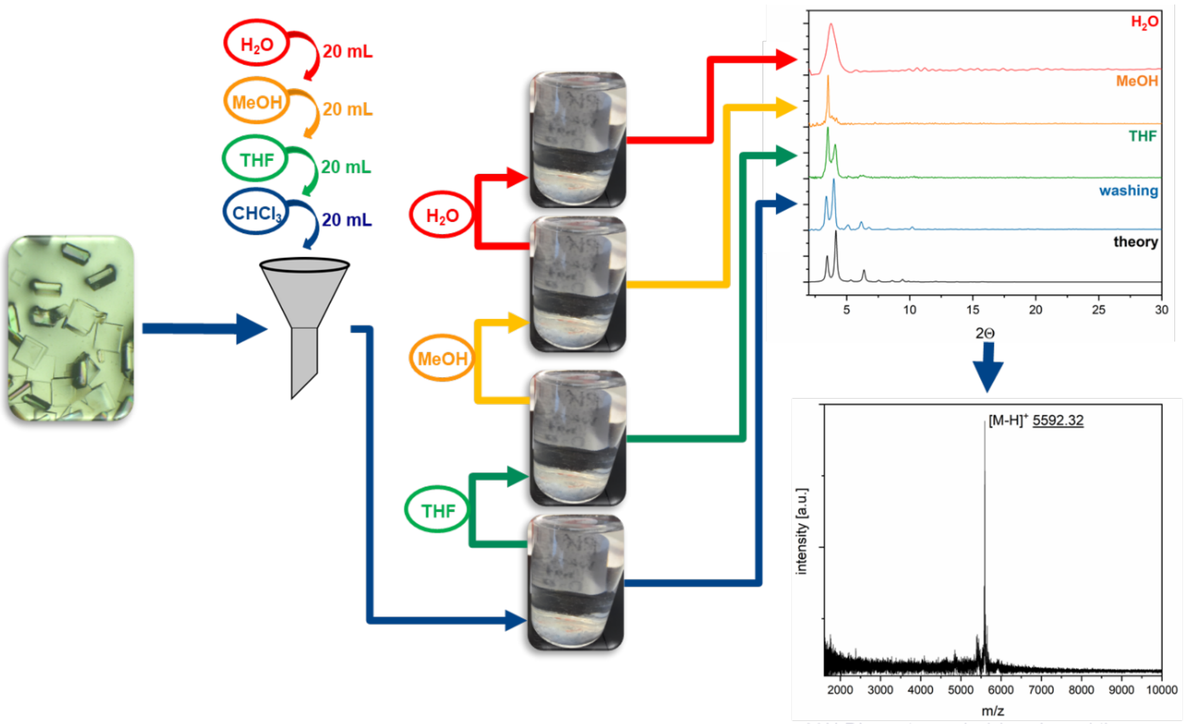

Figure S22. Schematic of the respective procedure for **6a** washing and storing steps and measurements.

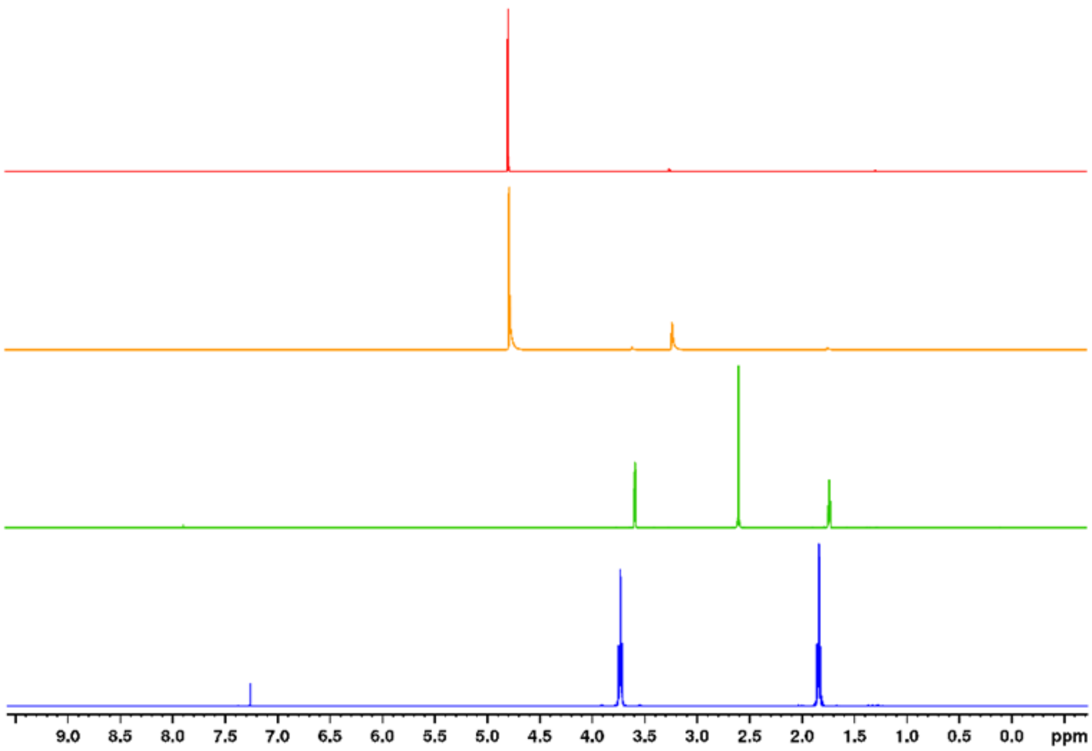

Figure S23. NMR spectra of the washing solutions for **6a** washing steps with $CDCl_3$ (blue), THF-d6 (green), CD_3OD (orange) and D_2O (red).

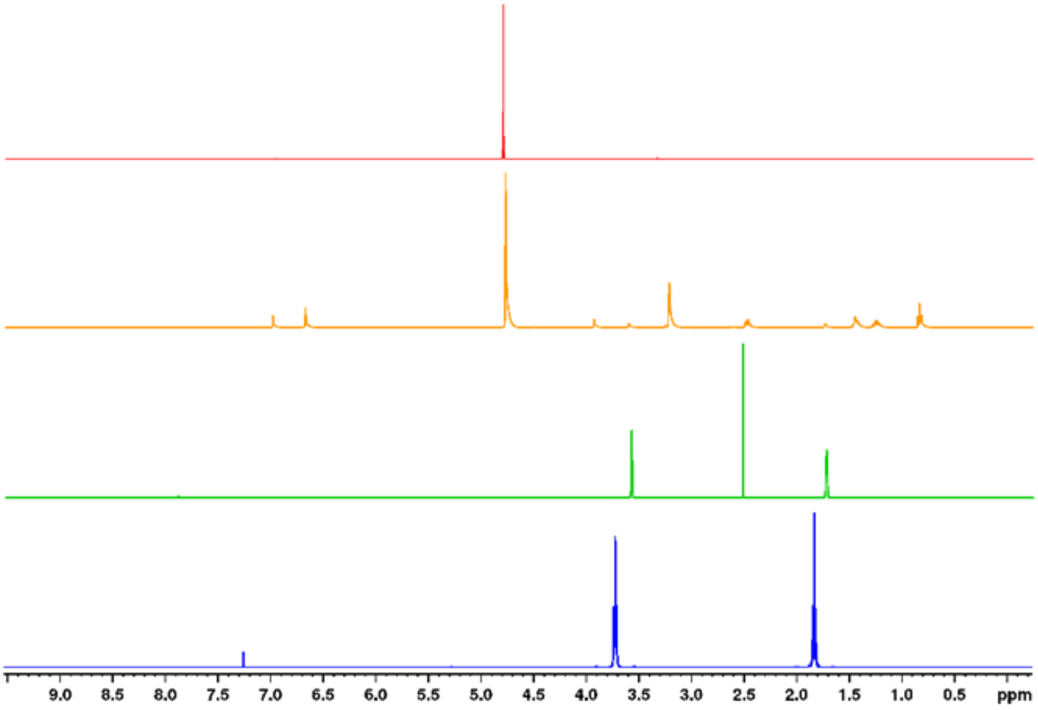

Figure S24. NMR spectra of the washing solutions for **7a** washing steps with $CDCl_3$ (blue), THF-d6 (green), CD_3OD (orange) and D_2O (red).

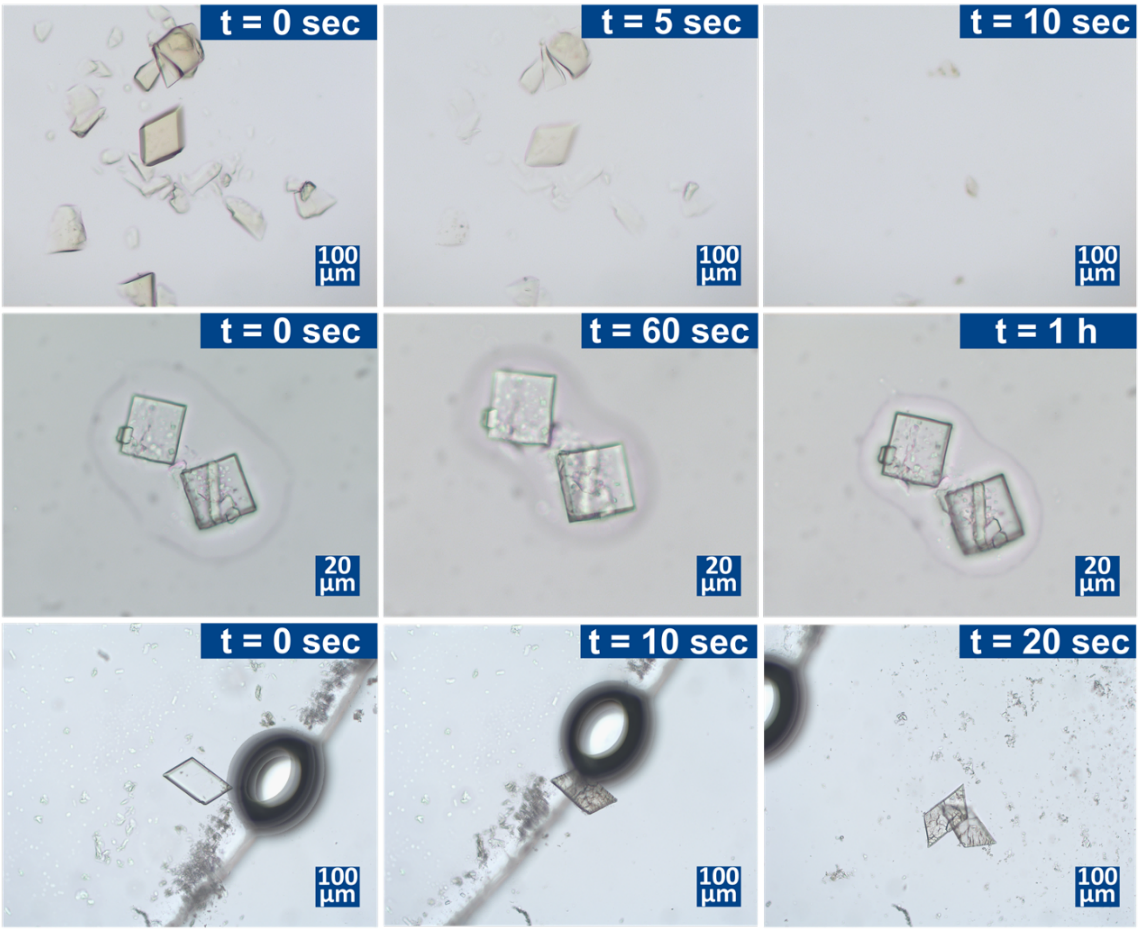

Figure S25. Microscopic images of **7a** (top) and **6a** (middle) after addition of MeOH after different times and **6a** (bottom) after addition of MeOH/AcOH (5:1).

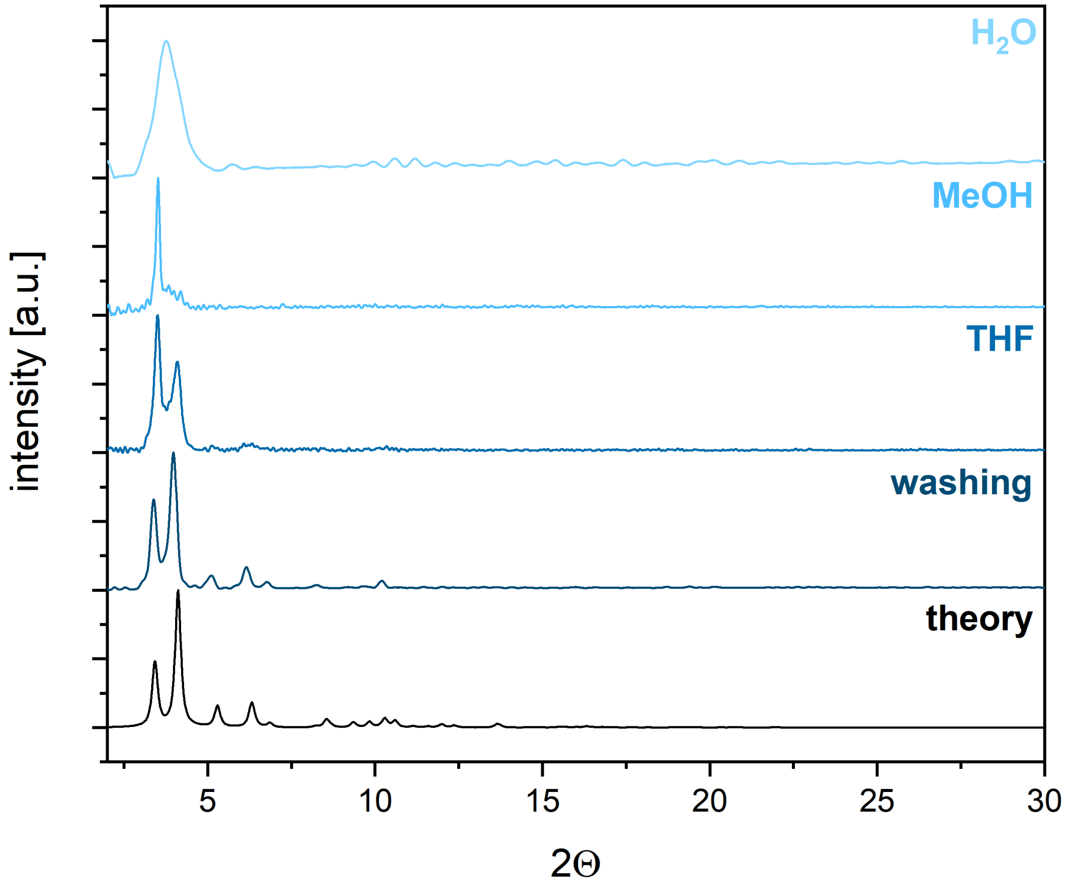

Figure S26. PXRD diffractograms of **6a** after respective storage.

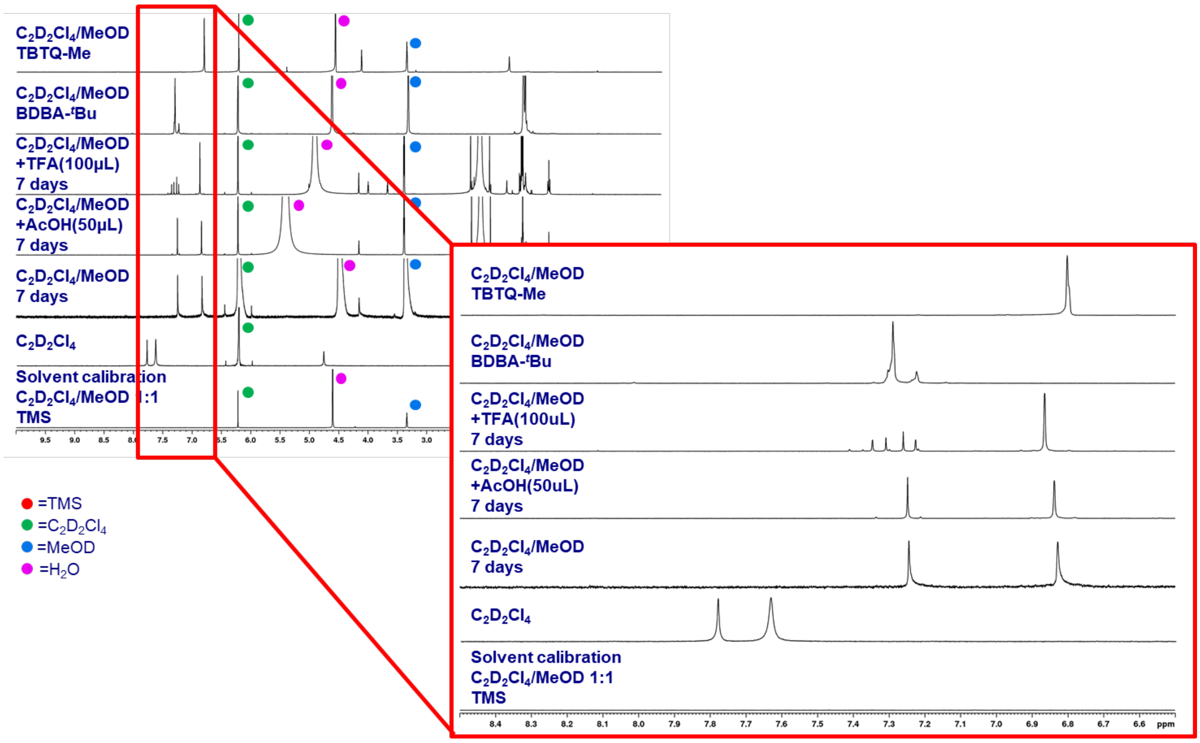

Figure S27. ^{1}H-NMR spectra of stability study of **6a** in $C_2D_2Cl_4$/MeOD (1:1).

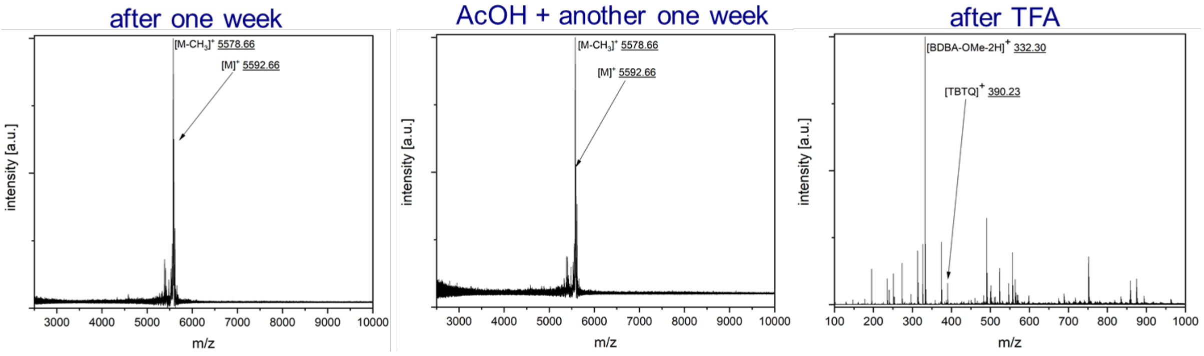

Figure S28. MALDI-TOF spectra ($C_2D_2Cl_4$/MeOD/$CHCL_3$, DCTB, positive) of corresponding ^{1}H-NMR samples.

10 Dye Adsorption and Water Oxidation Catalysis

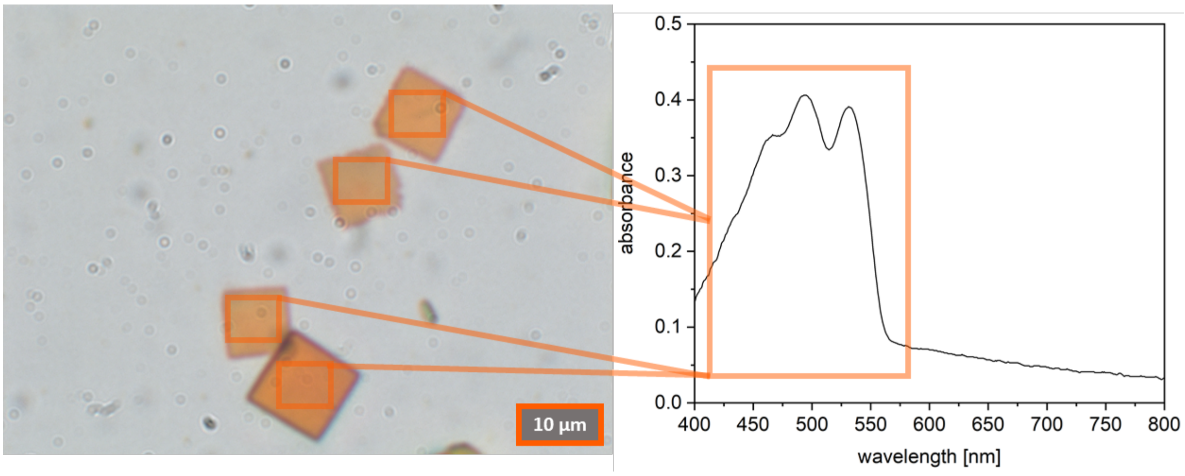

Figure S29. Microscopic images of **6a** with adsorption beta-carotene and absorption spectra of the crystals.

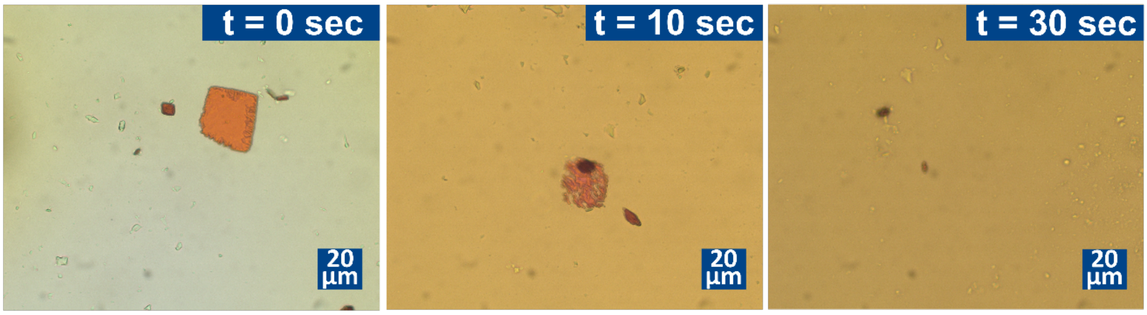

Figure S30. Microscopic images of **6a** with adsorped beta-Carotene after addition of DCM/AcOH at different times.

Calculation of q_e

The adsorption experiments were setup the following:

To a stock solution of beta-carotene (every time 0.001 L) varying concentrations of cage were added. The solutions were stirred over night for best adsorption of the beta-carotene. The cage was filtered of with a syringe filter. Starting from UV-Vis data of the filtered projecting solutions, UV-Vis spectra were recorded. Therefore, the projecting solutions were diluted with a factor of seven in order to fit the absorbance of the solutions to the maximum detection range of the device.

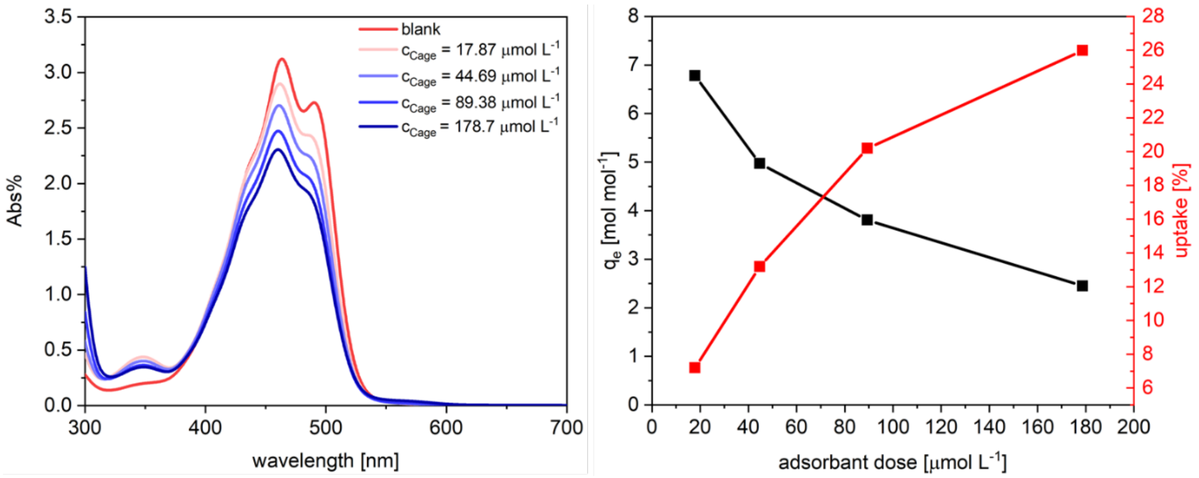

Figure S31. UV-Vis (CH_2Cl_2, 20 °C) study on the adsorption of beta-carotene.

With a freshly prepared calibration curve, the remaining concentration $\mathbf{c_e}$ **(measured)** of *β*-carotene was estimated. To get the correct value of the remainng concentrations $\mathbf{c_e}$ **(real)** the dilution was taken into account.

$$c_e(real) = c_e(measured) \cdot 7_{dilution}$$

In order to calculate $\mathbf{q_e}$ [mol/mol] the following formula was used:

$$q_e \left[\frac{mol}{mol}\right] = \frac{c_o - c_e}{n} V$$

With the inital concentration $\mathbf{c_0}$ **[mol/L]**, the remaining concentration $\mathbf{c_e}$ **[mol/L]**, the adsorbant dose n **[mol]** and the volume **V [L]**.

All calculated values are summarized in Table S2.

Table S2. Calculated values for *β*-carotene adsorption.

sample	OD	c_e(measured)[mol/L]	c_e(real)[mol/L]	c_{ads}[mol/L]	n_{6a}[mol]	q_e [mol/mol]
1(blank)	3.11	$2.41*10^{-4}$	$1.69*10^{-3}$	0	0	0
2	2.89	$2.23*10^{-4}$	$1.56*10^{-3}$	$0.13*10^{-3}$	$1.78*10^{-8}$	7.30
3	2.69	$2.09*10^{-4}$	$1.46*10^{-3}$	$0.23*10^{-3}$	$4.46*10^{-8}$	5.15
4	2.47	$1.92*10^{-4}$	$1.35*10^{-3}$	$0.34*10^{-3}$	$8.93*10^{-8}$	3.80
5	2.30	$1.78*10^{-4}$	$1.25*10^{-3}$	$0.44*10^{-3}$	$1.78*10^{-7}$	2.47

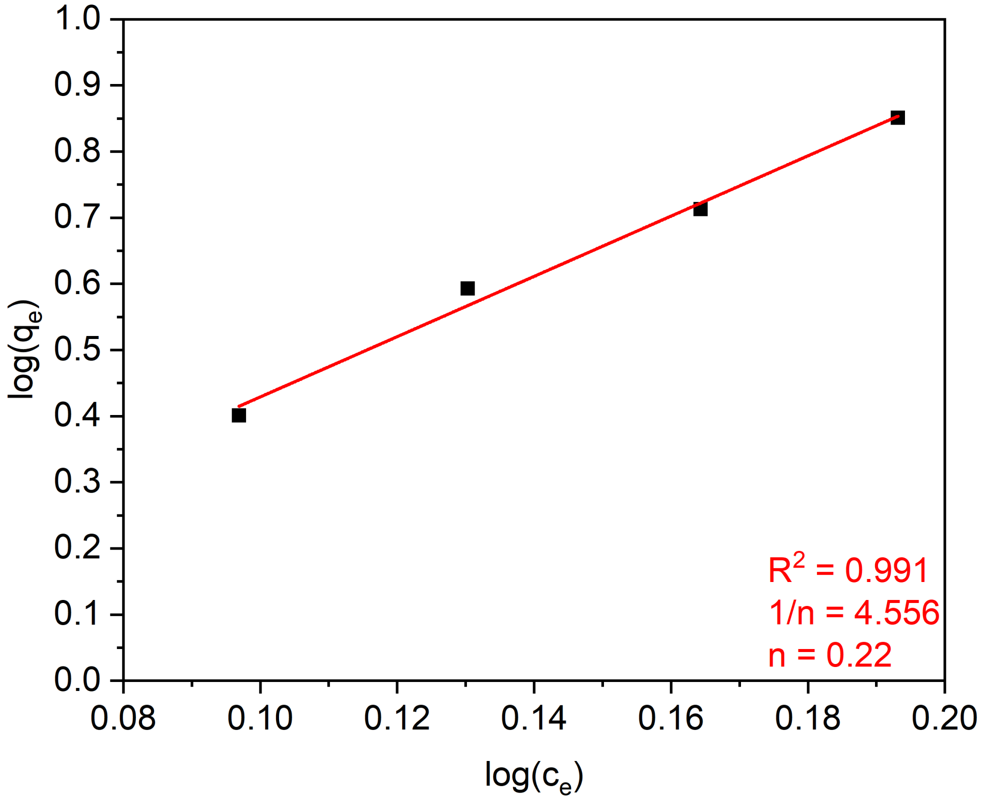

Figure S32. Linear fit according to the Freundlich model for the adsorption of *β*-carotene.

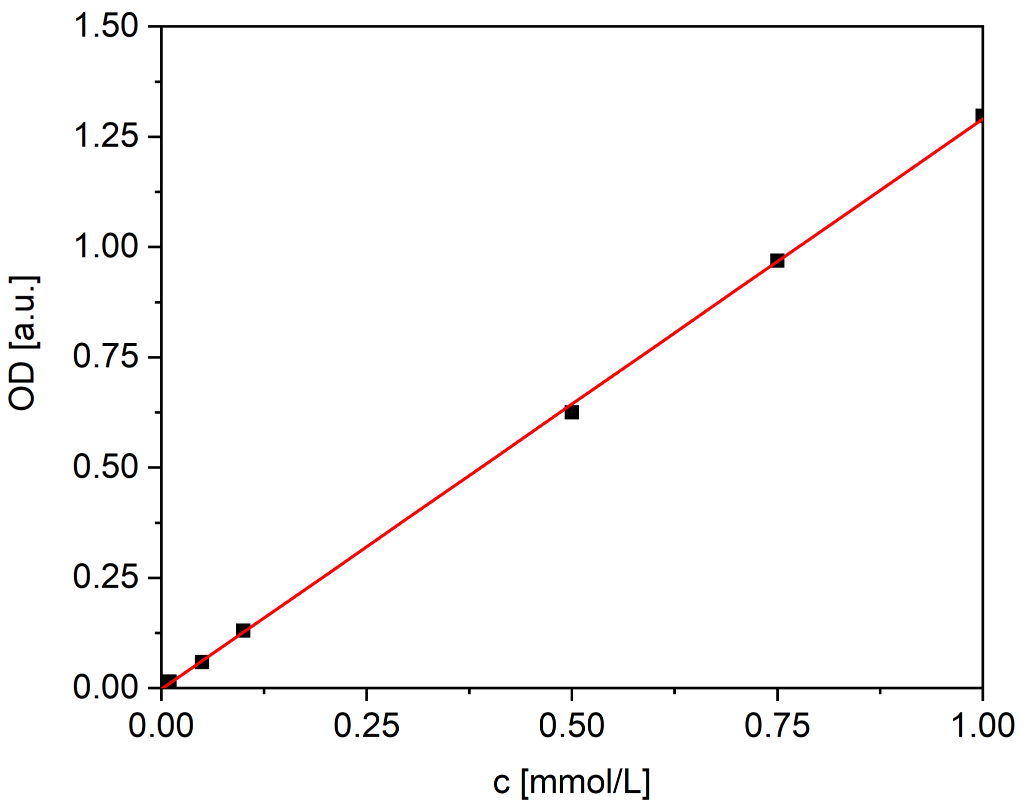

Figure S33. Calibration curve from UV-Vis spectra for beta-carotene at different concentrations for the adsorption experiments.

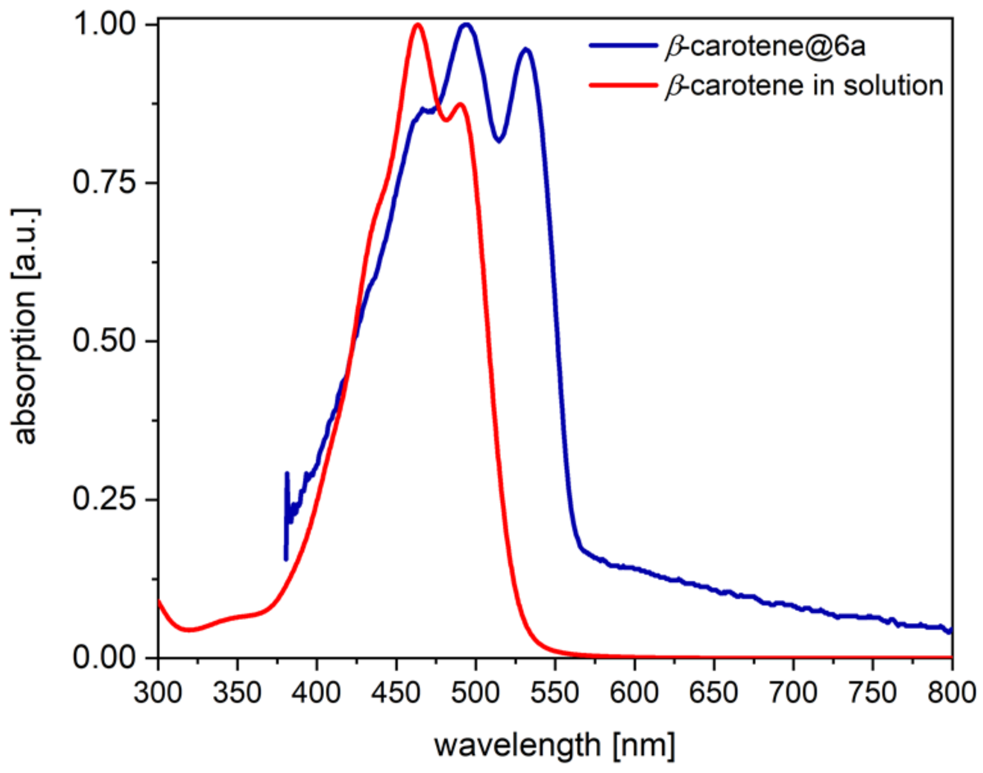

Figure S34. UV-Vis spectra (MeCN or solid, 20 °C) for beta-carotene in solid and liquid phase.

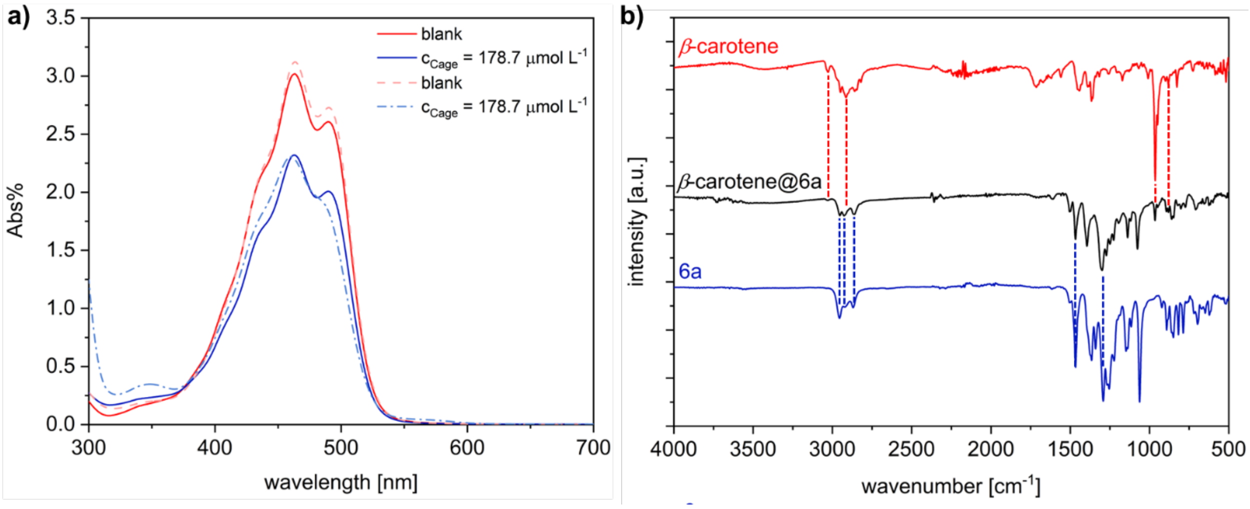

Figure S35. a) UV-Vis spectra of the stock solution (red) and the supernatant solution after adsorption of *β*-carotene with cage **6a** for two different measurements (solid and dashed lines) and b) FT-IR spectra (500-4000 cm^{-1}, rt, 16 scans) for **6a**, *β*-carotene and *β*-carotene@**6a** (characteristic peaks marked with dashed lines).

In accordance with the *β*-carotene adsorption experiment, the measurement and calculation for the adsorption of Ru(bda)(pic)$_2$ were performed in a similar manner.

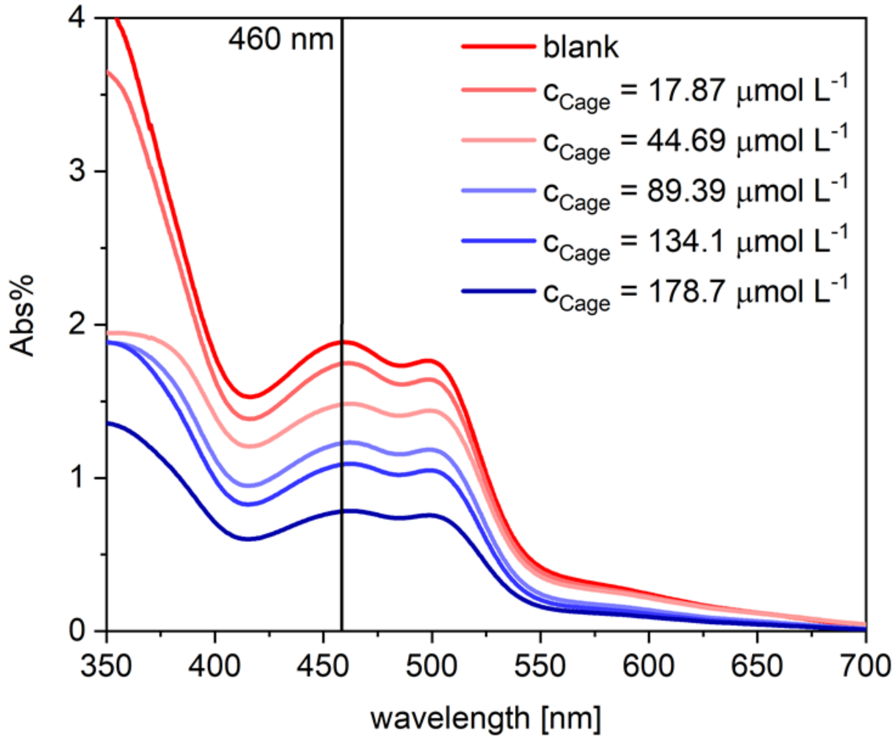

Figure S36. UV-Vis spectra (MeCN, 20 °C) of stock solution of **Ru(bda)(pic)$_2$** (c = 1.00 mmol/L) and remaining solution after adsorption (c = 0.835 mmol/L).

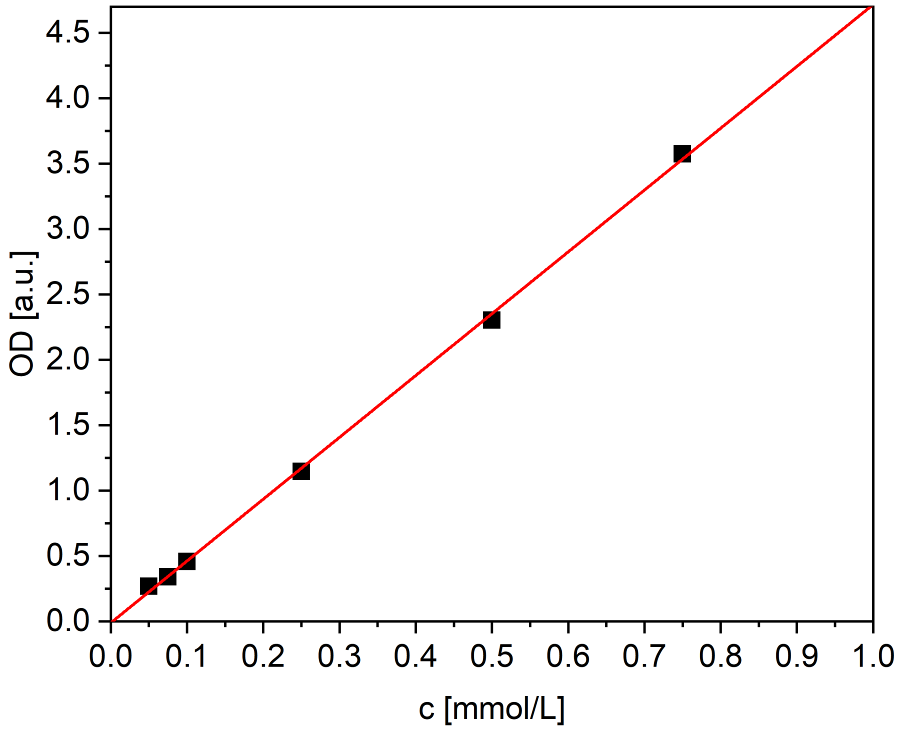

Figure S37. Calibration curve from UV-Vis spectra for **Ru(bda)(pic)$_2$** at different concentrations for the adsorption experiments.

Table S3. Calculated values for Ru(bda)(pic)$_2$ adsorption.

sample	OD	c_e(measured)[mol/L]	c_e(real)[mol/L]	c_{ads}[mol/L]	n_{6a}[mol]	q_e [mol/mol]
1(blank)	1.88	4.00*10^{-4}	1.00*10^{-3}	0	0	0
2	1.75	3.51*10^{-4}	8.78*10^{-4}	1.22*10^{-4}	1.78*10^{-8}	6.85
3	1.48	3.17*10^{-4}	7.93*10^{-4}	2.07*10^{-4}	4.46*10^{-8}	4.64
4	1.23	2.61*10^{-4}	6.53*10^{-4}	3.47*10^{-4}	8.93*10^{-8}	3.88
5	0.78	1.69*10^{-4}	4.23*10^{-4}	5.77*10^{-4}	1.78*10^{-7}	3.23

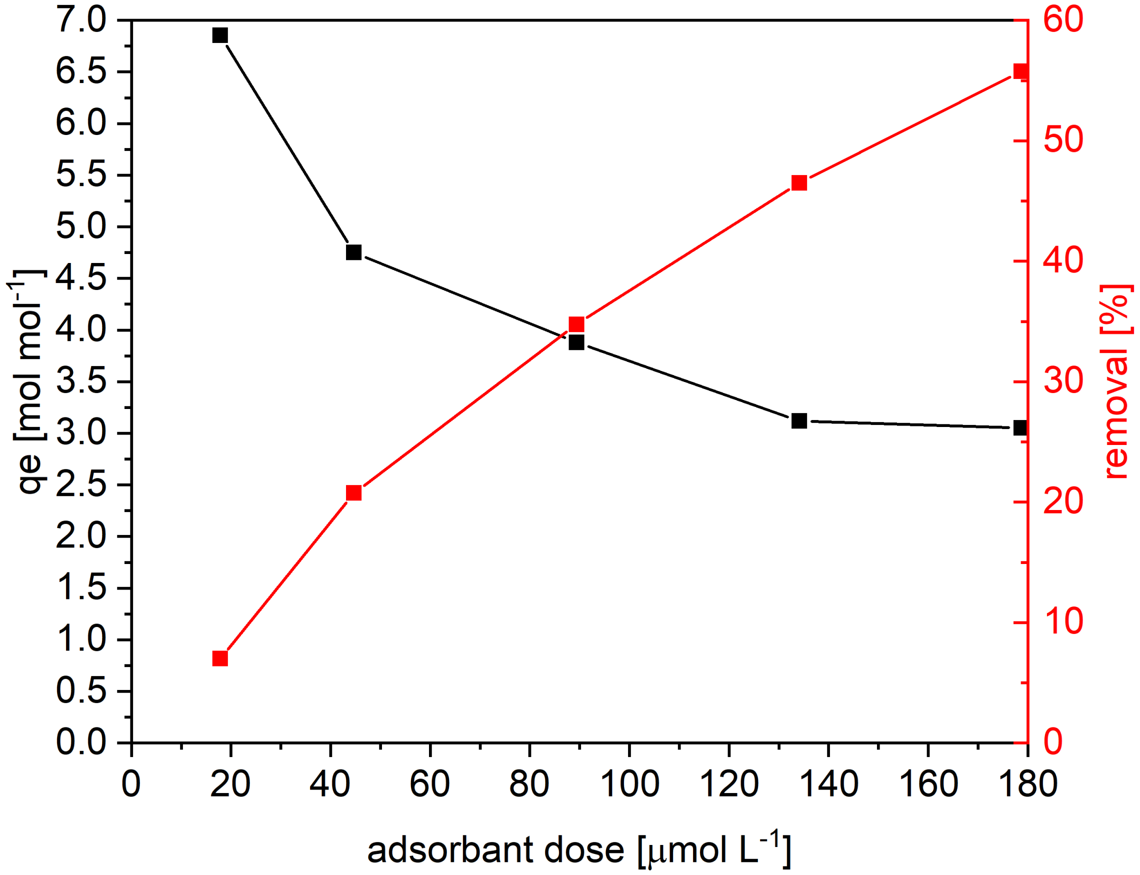

Figure S38. Evaluation of adsorption process for **Ru(bda)(pic)$_2$**.

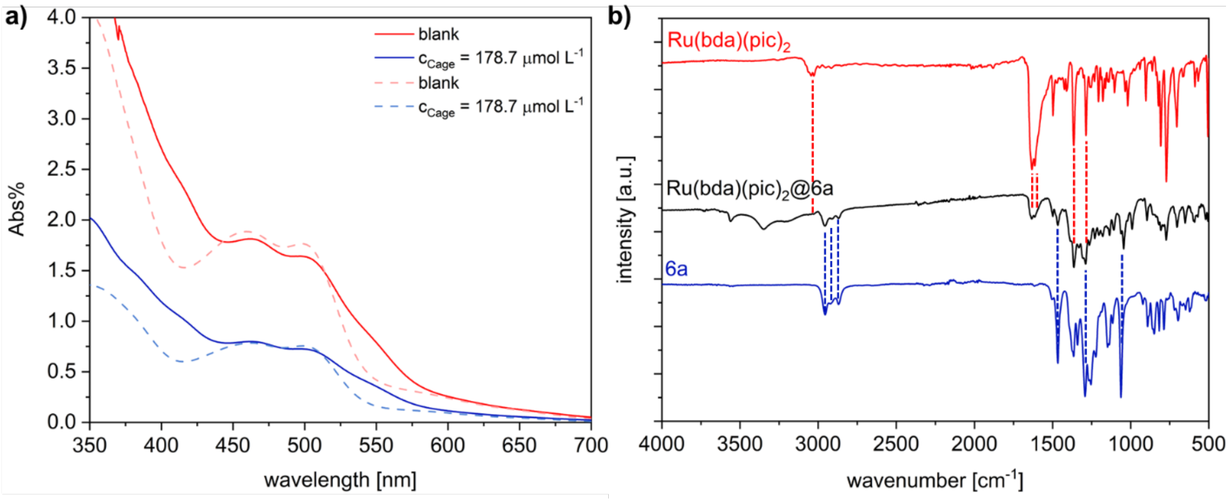

Figure S39. a) UV-Vis spectra of the stock solution (red) and the supernatant solution after adsorption of Ru(bda)(pic)$_2$ with cage **6a** for two different measurements (solid and dashed lines) and b) FT-IR spectra (500-4000 cm^{-1}, rt, 16 scans) for **6a**, Ru(bda)(pic)$_2$ and Ru(bda)(pic)$_2$@**6a** (characteristic peaks marked with dashed lines).

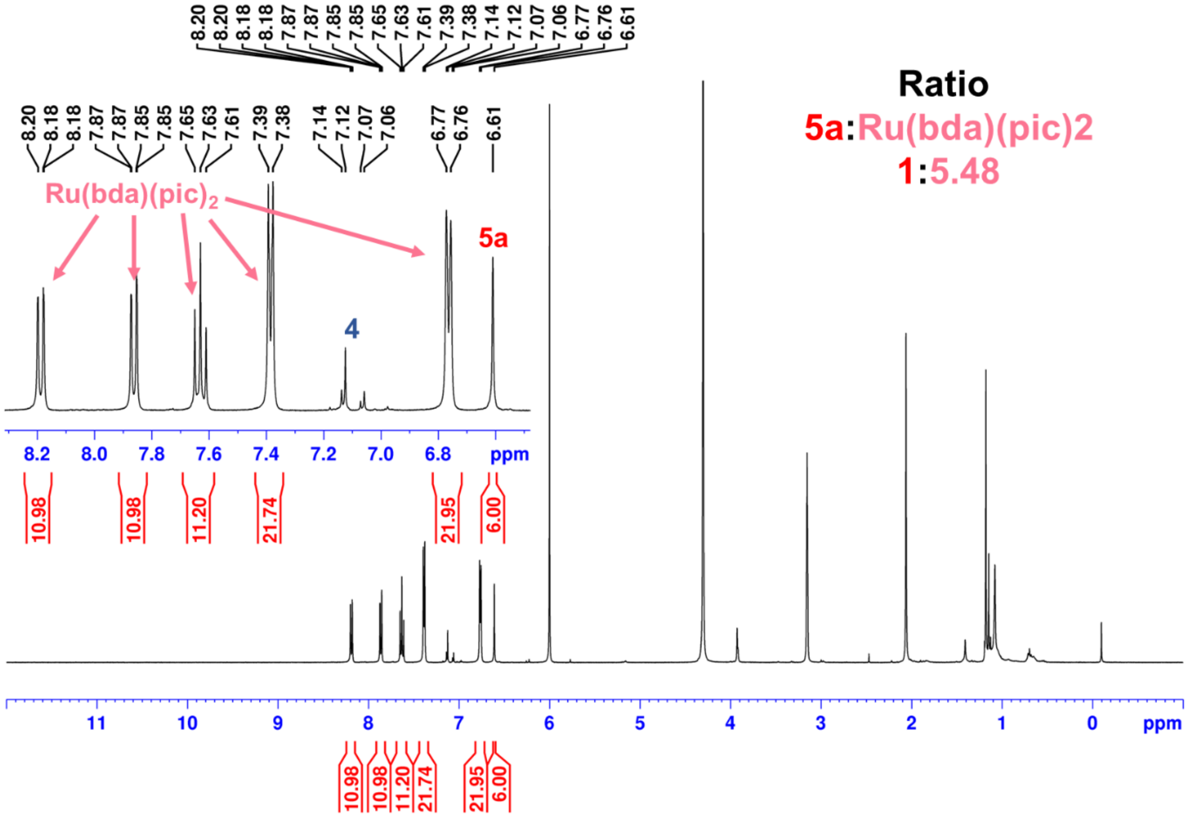

Figure S40. ^{1}H-NMR spectrum (400 MHz, rt, $C_2D_2Cl_4$, MeOD, TFA-d_1, 128 scans) after full decomposition of Ru(bda)(pic)$_2$@**6a** under acidic conditions. Ratio between the two components was estimated based on NMR integration.

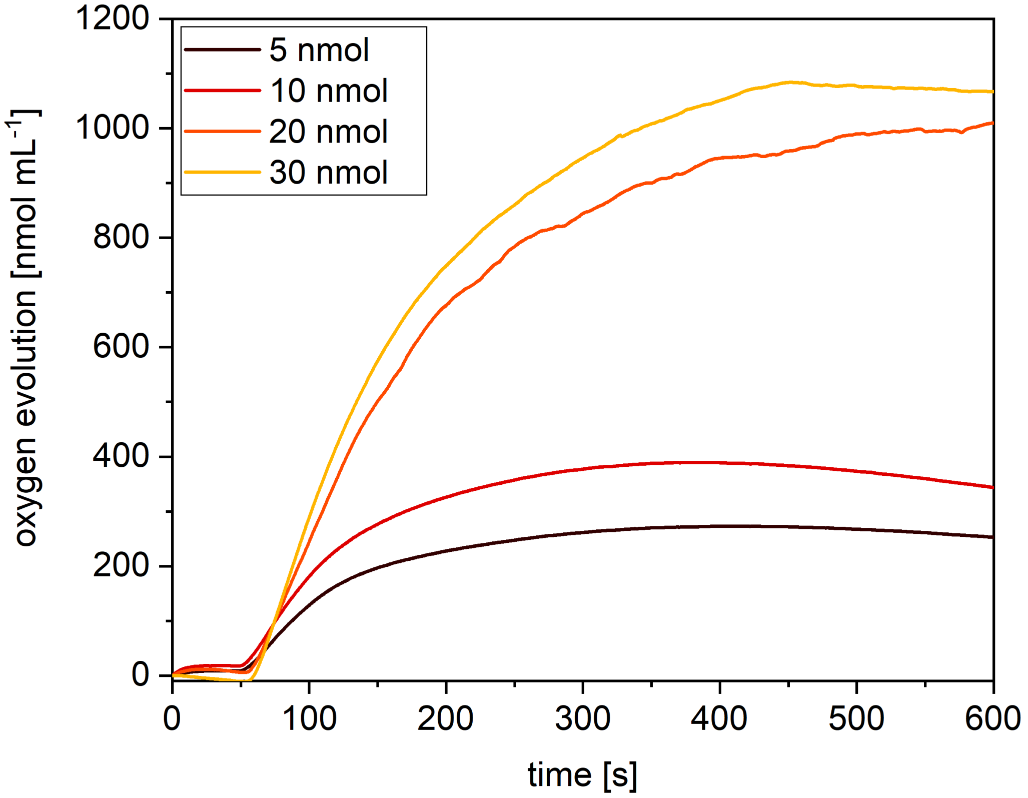

Figure S41. Photocatalytic water oxidation experiment of R**u(bda)(pic)$_2$⊂6a** in a three component system in MeCN/H_2O (40:60).

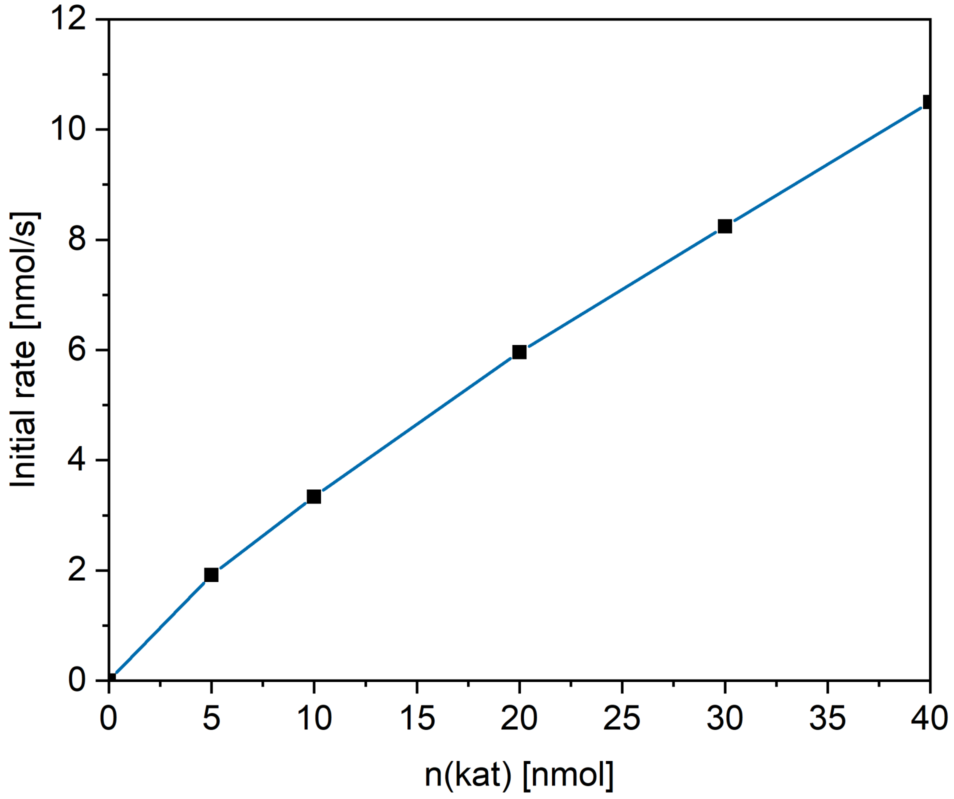

Figure S42. Initial rate of photocatalytic water oxidation vs catalyst loading for R**u(bda)(pic)$_2$⊂6a**.

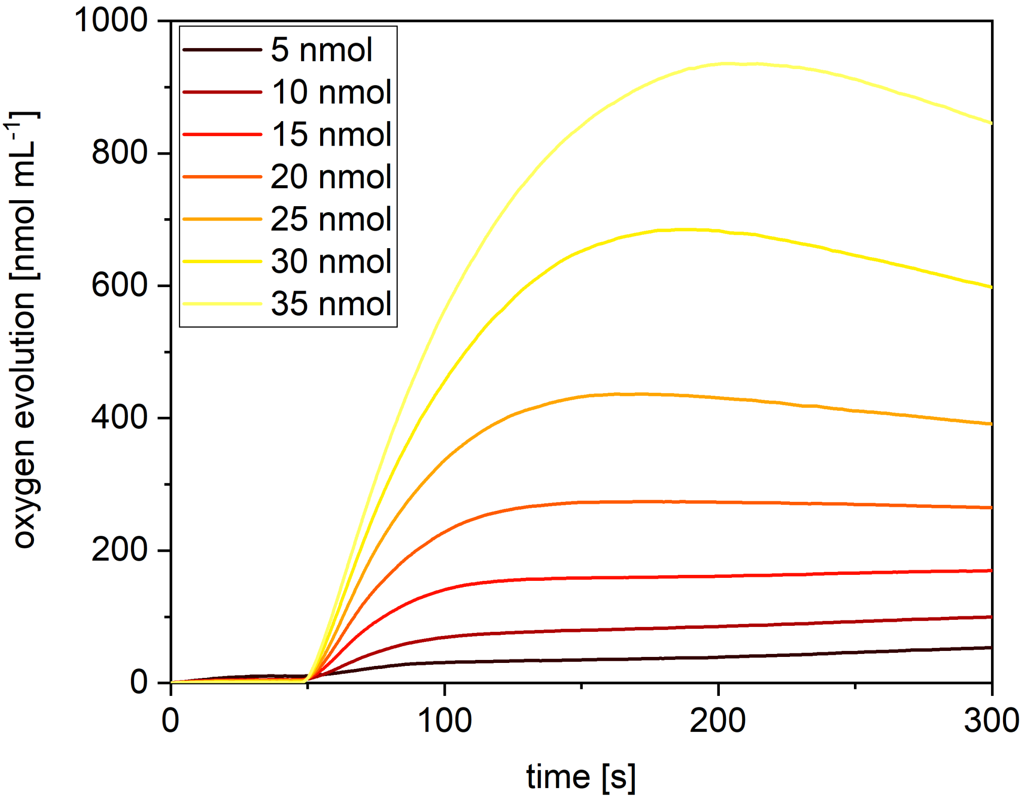

Figure S43. Photocatalytic water oxidation experiment of **Ru(bda)(pic)$_2$** in a three component system in MeCN/H_2O (40:60).

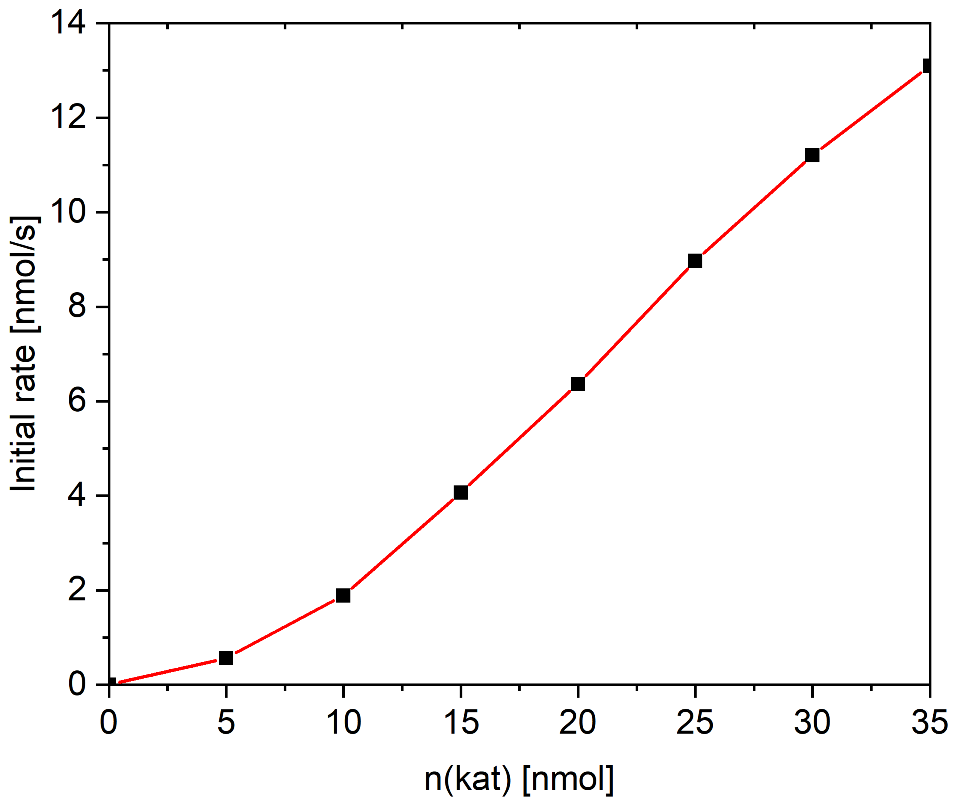

Figure S44. Initial rate of photocatalytic water oxidation vs catalyst loading for **Ru(bda)(pic)$_2$**.

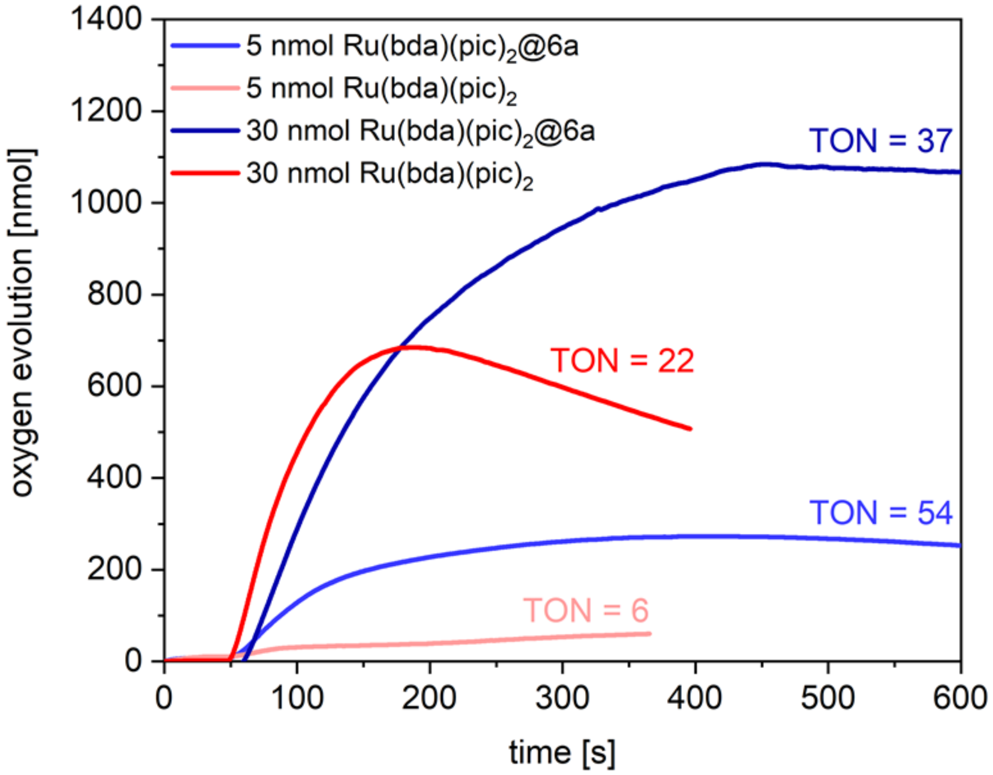

Figure S45. Comparison of the photocatalytic water oxidation performance of **Ru(bda)(pic)$_2$⊂6a** (blue) and **Ru(bda)(pic)$_2$** (red) at 5 nmol and 30 nmol of catalyst loading.

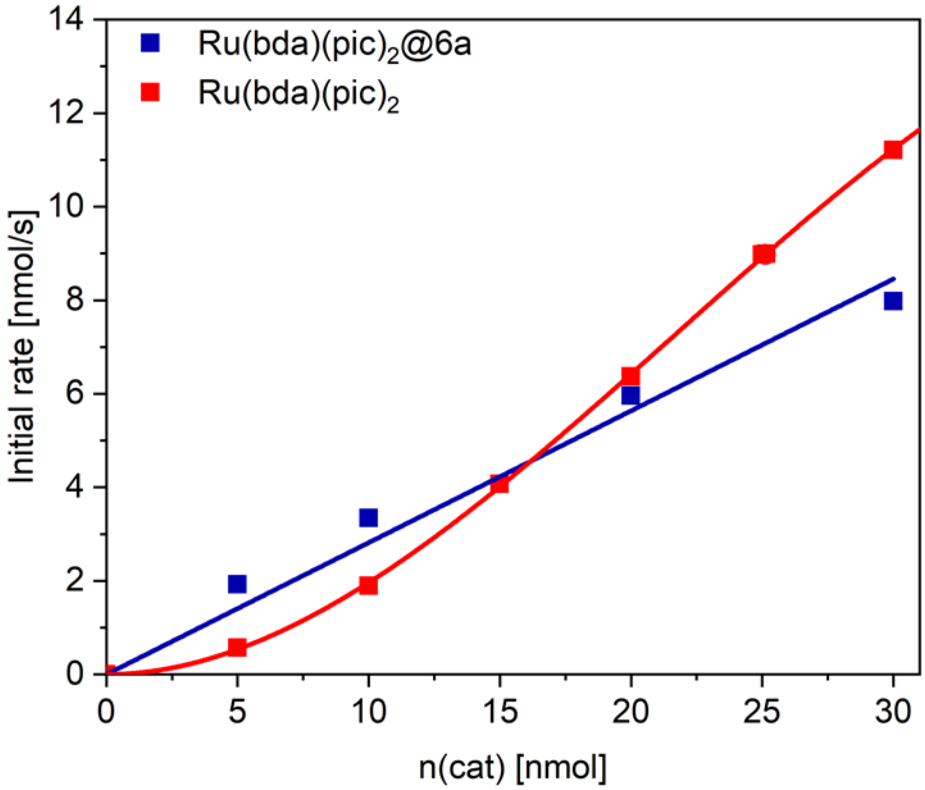

Figure S46. Comparison of initial rate of photocatalytic water oxidation vs catalyst loading for R**u(bda)(pic)$_2$⊂6a** (blue) and R**u(bda)(pic)$_2$**(red).

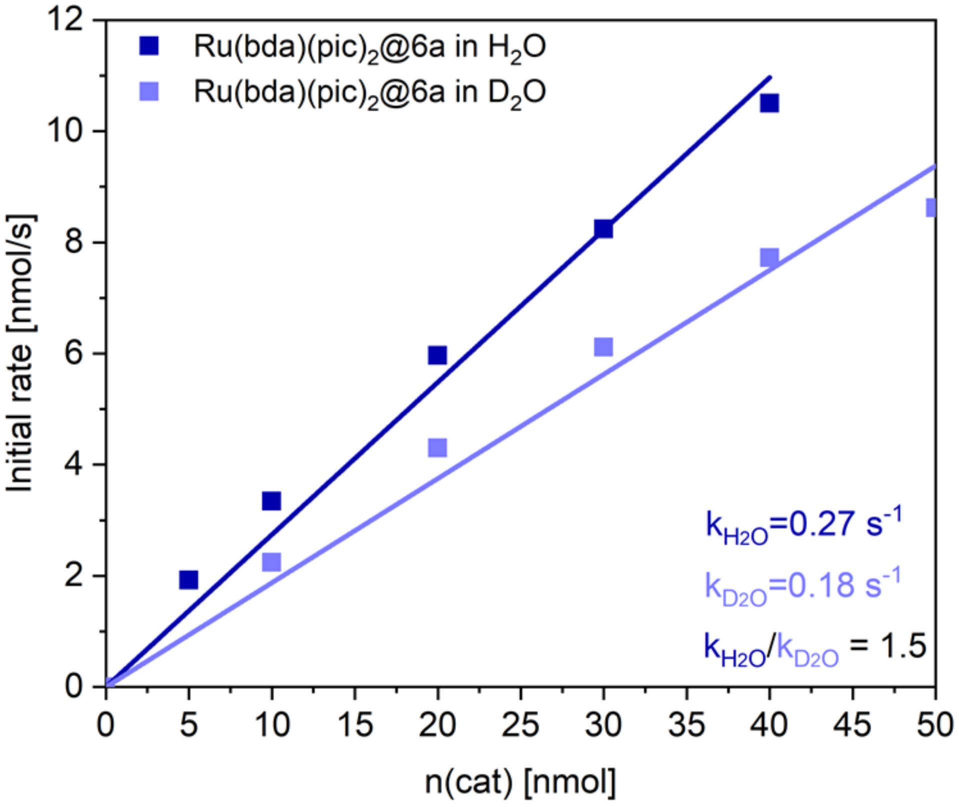

Figure S47. Kinetic isotope effect for **Ru(bda)(pic)₂⊂6a.**

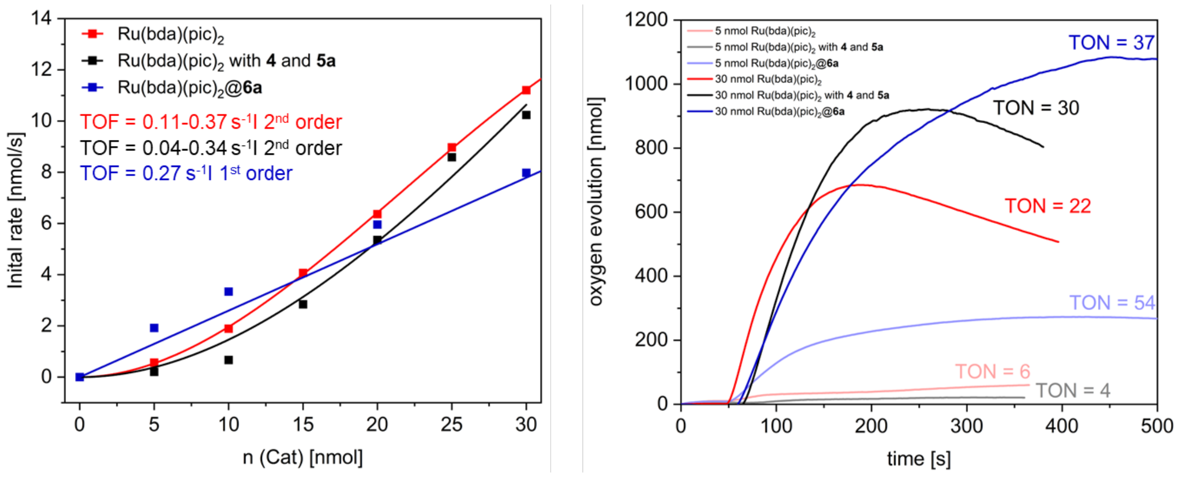

Figure S48. Comparison of the photocatalytic water oxidation with pristine Ru(bda)(pic)₂ (red), Ru(bda)(pic)₂ +**4**+**5a** (black) and Ru(bda)(pic)₂@**6a** (blue).

Table S4. Catalytic performance for state-of-the-art molecular Ru(bda) catalysts for photochemical water oxidation and comparison with Ru(bda)(pic)$_2$@**6a**.

Catalyst	TON	TOF	reference
**Ru(bda)(pic)$_2$**	10	0.35 s^{-1}	*Inorg. Chem.*, **2010**, *49*, 1, 209-215
**Ru(bda)(pic)$_2$**	22	0.11 – 0.37 s^{-1}	*this work*
**Ru(bda)(pic)$_2$@6a**	54	0.27 s^{-1}	*this work*
**Ru(bda)(isoq)$_2$**	140	0.25 s^{-1}	*ACS Catal.*, **2020**, *10*, 1, 580-585
**M1**	10	0.05 – 0.1 s^{-1}	*Nat. Catal.*, **2022**, *5*, 867-877
**M2**	105	0.4 – 3.2 s^{-1}	*Nat. Catal.*, **2022**, *5*, 867-877
**2C**	400	5.5 s^{-1}	*Angew. Chem. Int. Ed.*, **2023**, *62*, e202217745
**3C**	150	14 s^{-1}	*Angew. Chem. Int. Ed.*, **2023**, *62*, e202217745
**4C**	2200	15.5 s^{-1}	*Angew. Chem. Int. Ed.*, **2023**, *62*, e202217745
**MC3**	430	10.9 s^{-1}	*Chem. Sci.*, **2020**, *11*, 7654-7664
***p*-Me-MC3**	120	1.7 s^{-1}	*Chem. Sci.*, **2020**, *11*, 7654-7664

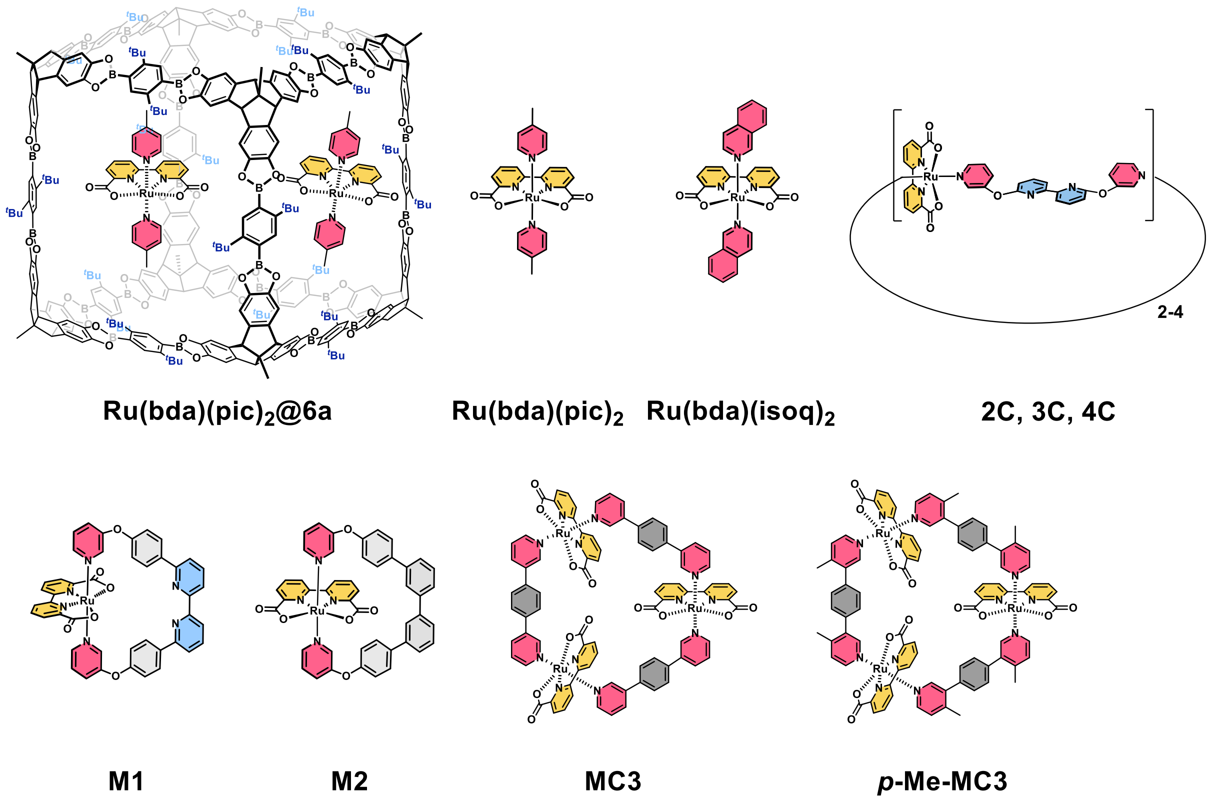

Figure S49. Structures of the catalyst described in Table S4.

11 Single-Crystal X-ray Diffraction

Single crystal X-ray diffraction data for **6a** were collected at the P11 beamline at DESY. The diffraction data were collected by a single 360 ° ϕ scan at 100 K. The diffraction data were indexed, integrated, and scaled using the XDS program package.[6] The structure was solved using SHELXT,[7] expanded with Fourier techniques and refined using the SHELXL.[8] All non-hydrogen atoms in the main residue were refined anisotropically. Stereochemical restrains (DFIX, DANG, and FLAT) were generated by the GRADE program (https://grade.globalphasing.org/cgi-bin/grade2_server.cgi) and applied during refinement. Additional restrains by FLAT, SADI, RIGU, and SIMU were manually added to fix unfavourable geometries and ellipsoids. The structure was solved as twins using HKLF5 file generated by the TwinRotMat routine implemented in the PLATON program package (twin operation (0 1 0 1 0 0 0 0 −1)).[9] Electron density deriving from the large solvent accessible voids were removed by the SQUEEZE[10] routine implemented in PLATON.[9]

Supplementary crystallographic data for cage **6a** can be obtained free of charge from The Cambridge Crystallographic Data Centre via the following link (CCDC number 2267750): http://www.ccdc.cam.ac.uk/data_request/cif.

The checkcif routine implemented in the PLATON software package[9] generated several level A and B alerts, for which explanations are listed below.

_vrf_PLAT023_pk004_a_tw_sq

;

PROBLEM: Resolution (too) Low [sin(theta)/Lambda < 0.6].. 0.28 Ang-1

RESPONSE: Due to large thermal factor of the cage molecule diffraction data were weak at resolution shells lower than 2.12 Å. The resolution shells below 1.77 Å were not included in the integration and scaling processes to maximize data quality.

;

_vrf_PLAT082_pk004_a_tw_sq

;

PROBLEM: High R1 Value .... 0.23 Report

RESPONSE: The high R_1 value is due to large thermal factor of the cage molecule.

;

_vrf_PLAT084_pk004_a_tw_sq

;

PROBLEM: High wR2 Value (i.e. > 0.25) .... 0.60 Report

RESPONSE: The high wR_2 value is due to large thermal factor of the cage molecule.

;

_vrf_PLAT088_pk004_a_tw_sq

;

PROBLEM: Poor Data / Parameter Ratio .... 2.82 Note

RESPONSE: The low data / parameter ratio for this structure is due to the resolution cut off at 1.77 Å.

;

_vrf_PLAT412_pk004_a_tw_sq

;

PROBLEM: Short Intra XH3 .. XHn H6_11 ..H14A_11 . 1.68 Ang.

RESPONSE: Due to low resolution of the data the AFIX 33 instruction was used for all methyl groups. This treatment fixed the rotation of methyl group at geometrically ideal angle and caused short intra- or inter-molecular H–H contacts.

;

_vrf_PLAT241_pk004_a_tw_sq

;

PROBLEM: High 'MainMol' Ueq as Compared to Neighbors of O2_2 Check

RESPONSE: Due to large thermal factor of the cage molecule some atoms have high/low U_{eq} compared to those of the neighbouring molecules.

;

_vrf_PLAT242_pk004_a_tw_sq

;

PROBLEM: Low 'MainMol' Ueq as Compared to Neighbors of C3_2 Check

RESPONSE: Due to large thermal factor of the cage molecule some atoms have high/low *U*eq compared to those of the neighbouring molecules.

;

_vrf_PLAT260_pk004_a_tw_sq

;

PROBLEM: Large Average Ueq of Residue Including O1_2 0.746 Check

RESPONSE: Large average U_{eq} is due to large solvent accessible voids.

;

_vrf_PLAT340_pk004_a_tw_sq

;

PROBLEM: Low Bond Precision on C-C Bonds .... 0.04976 Ang.

RESPONSE: The low bond precision for this structure is due to low resolution limit.

;

_vrf_PLAT413_pk004_a_tw_sq

;

PROBLEM: Short Inter XH3 .. XHn H23C_2 ..H13C_11 . 1.93 Ang.

RESPONSE: Due to low resolution of the data the AFIX 33 instruction was used for all methyl groups. This treatment fixed the rotation of methyl group at geometrically ideal angle and caused short intra- or inter-molecular H–H contacts.

;

Table S5. Crystal data and structure refinement for **6a**.

Identification code	pk004_a_tw_sq	
CCDC number	2267750	
Empirical formula	$C_{352}H_{336}B_{24}O_{48}$	
Formula weight	5593.63	
Temperature	100(2) K	
Wavelength	0.61992 Å	
Crystal system	Tetragonal	
Space group	*I*4/m	
Unit cell dimensions	a = 23.59(2) Å	α = 90°.
	b = 23.59(2) Å	β = 90°.
	c = 51.53(4) Å	γ = 90°.
Volume	28673(58) $Å^3$	
Z	2	
Density (calculated)	0.648 g/cm^3	
Absorption coefficient	0.032 mm^{-1}	
$F(000)$	5904	
Crystal size	0.200 × 0.200 × 0.200 mm^3	
Theta range for data collection	0.689 to 10.058°.	
Index ranges	$-8 \leq h \leq 13$, $-8 \leq k \leq 13$, $-28 \leq l \leq 28$	
Reflections collected	1361	
Independent reflections	1361 [R(int) = 0.0782]	
Completeness to theta = 10.058°	97.9%	
Absorption correction	None	
Refinement method	Full-matrix least-squares on F^2	
Data / restraints / parameters	1361 / 1210 / 482	
Goodness-of-fit on F^2	2.553	
Final R indices [$I > 2\sigma(I)$]	R_1 = 0.2305, wR_2 = 0.5356	
R indices (all data)	R_1 = 0.2927, wR_2 = 0.5967	
Extinction coefficient	n/a	
Largest diff. peak and hole	0.243 and –0.294 e·$Å^{-3}$	

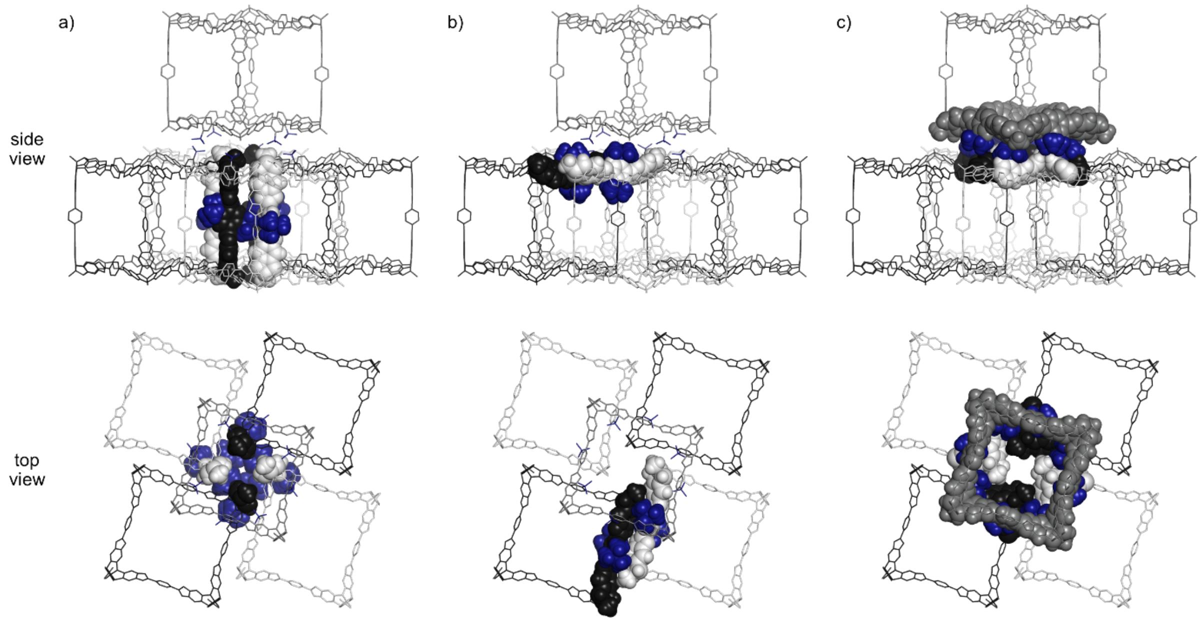

Fig. S50. Detailed views of dispersion interactions between *t*-Bu groups (blue) and phenyl rings (different shades of grey for adjacent cages) for edge-to-edge contacts a), b) within and c) between the cage layers.

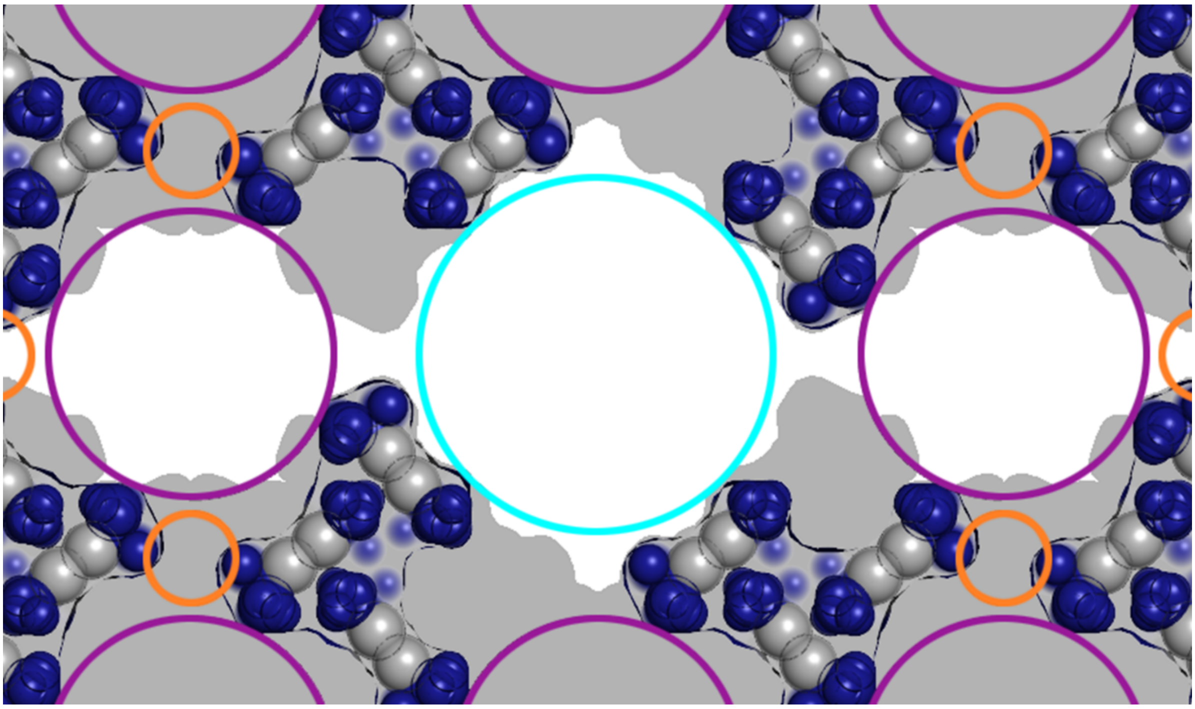

Fig. S51. Thin section cut of a mesoporous defect (cyan) with one cage missing in the crystal packing (cage windows in orange, intrinsic pores in purple; the occupied area after 360° rotation along the channel axis is indicated in grey.

12 Semiempirical calculations

As a first approximation for the mechanism of the exchange of oxygen substituents at the trigonal boron of a boronic acid under neutral conditions, we postulate a stepwise mechanism as shown in Fig. S52. Initial addition of a catechol to the boronic acid results in a tetragonal borate structure which is further stabilized by a hydrogen bond from the free OH of the catechol. Proton transfer from the coordinated OH of the catechol to an OH group at boron generates H_2O as a leaving group, which is subsequently released under formation of a trigonal intermediate with a simply bound catechol. In the next step, coordination of the free OH of the catechol results in a tetragonal borate structure. After proton transfer to the OH group at boron, the second H_2O is released under formation of the trigonal boronate ester. In the reverse direction, nucleophilic attack of H_2O induces the backwards hydrolysis of the boronate ester.

For an initial assessment of the relative stability of the intermediates for this esterification and hydrolysis mechanism, we calculated heats of formation for geometry-optimized structures of all relevant intermediates for two model reactions between catechol and the two isomeric coupling partners 2,4-di-*n*-butylphenyl boronic acid (light blue in Fig.S52) or 2,4-di-*t*-butylphenyl boronic acid (dark blue in Fig. S52). To account for the released water, we also calculated the heat of formation in the gas phase for H_2O.

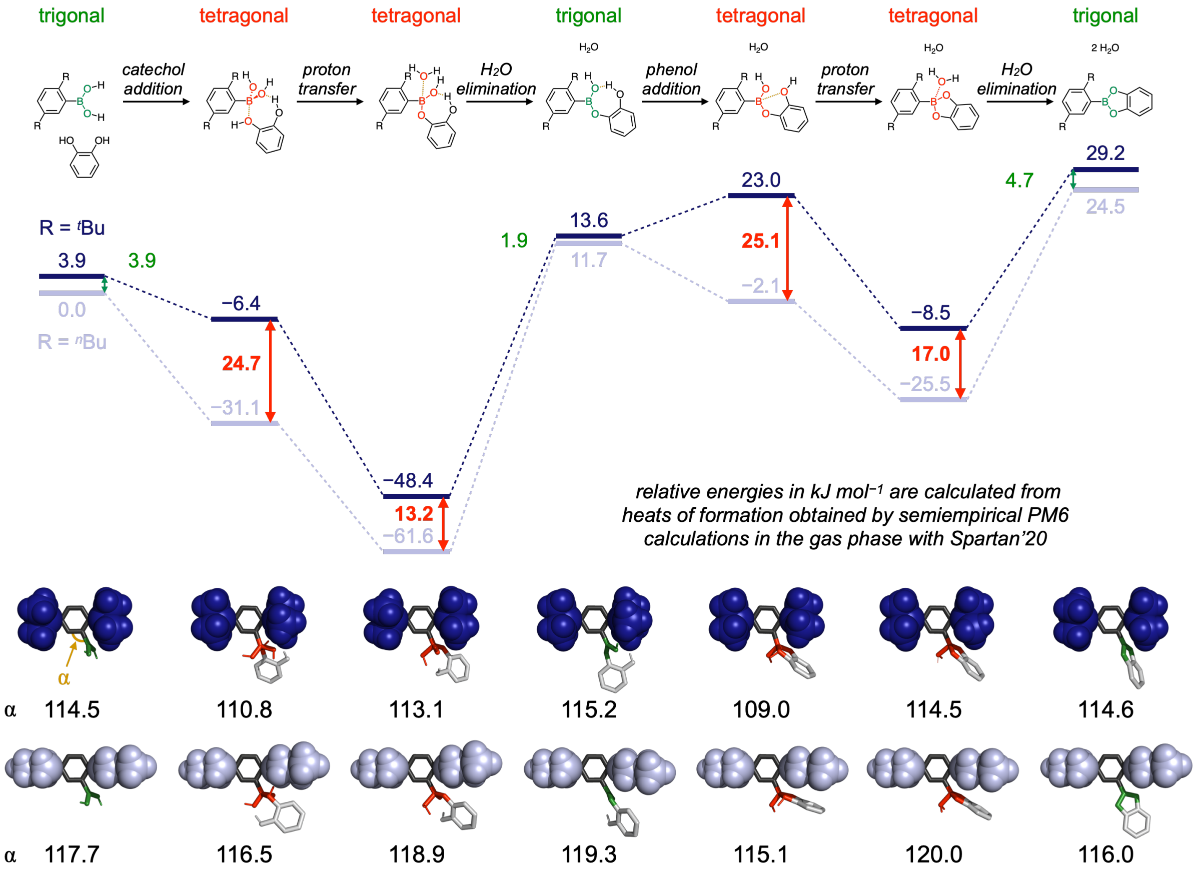

Figure S52. PM6-Calculations in the gas phase for the formation of model boronate ester from two boronic acids (with *tert*-butyl or *n*-butyl chains in *ortho*-position to the boron center) and a catechol unit.

Please note that all calculations have been performed in the gas phase and no solvation or other effects in condensed phase have been considered. Therefore, the energy diagram presented in Fig. S52 does not correlate to a proper reaction pathway, as the stability of tetragonal

intermediates is overestimated in the gas phase due to attractive Lewis acid-base interactions, that cannot be compensated by solvation of the polar groups in the trigonal intermediates. However, we still use these calculations for a first estimation of the relative energies between the two model reactions.

As it is shown in Fig. S52, there are only minor differences in energy between the *n*-butyl and the *t*-butyl compound for the structures with trigonal coordinate boron (1.9–4.7 kJ mol^{-1} in favor of the *n*-butyl derivative). Apparently, the sterically less demanding trigonal configuration is only marginally affected by the *t*-butyl groups. However, this energy differences raise significantly for the tetragonally coordinated intermediates (17–25 kJ mol^{-1} in favor of the *n*-butyl derivative). Here, the *t*-butyl group in *ortho*-position restricts the formation of the sterically more demanding formation of a tetragonally coordinate boron via addition of a fourth substituent. According to these differences in energy for the tetragonal intermediates, we also assume significantly higher transition states for the esterification or hydrolysis of the *t*-butyl derivative. Therefore, we propose that the stabilizing effect of the *t*-butyl groups in DBA **4** is of kinetic nature, as the exchange of oxygen substituents at boron in *ortho*-position is significantly slowed down by a substantial increase in energy for any trigonally coordinated transition states or intermediates due to steric interactions with the *t*-butyl groups.

13 References

1. Burkhardt, A.; Pakendorf, T.; Reime, B.; Meyer, J.; Fischer, P.; Stübe, N.; Panneerselvam, S.; Lorbeer, O.; Stachnik, K.; Warmer, M.; Rödig, P.; Göries, D.; Meents, A., Status of the crystallography beamlines at PETRA III. *Eur. Phys. J. Plus.* **2016,** *131*, 56.
2. Vile, J.; Carta, M.; Bezzu, C. G.; McKeown, N. B., Tribenzotriquinacene-based polymers of intrinsic microporosity. *Polym. Chem.* **2011,** *2*, 2257−2260.
3. Klotzbach, S.; Scherpf, T.; Beuerle, F., Dynamic covalent assembly of tribenzotriquinacenes into molecular cubes. *Chem. Commun.* **2014,** *50*, 12454−12457.
4. Timmer, B. J. J.; Kravchenko, O.; Zhang, B.; Liu, T.; Sun, L., Electronic Influence of the 2,2′-Bipyridine-6,6′-dicarboxylate Ligand in Ru-Based Molecular Water Oxidation Catalysts. *Inorg. Chem.* **2021,** *60*, 1202−1207.
5. Perlmutter, J. I.; Forbes, L. T.; Krysan, D. J.; Ebsworth-Mojica, K.; Colquhoun, J. M.; Wang, J. L.; Dunman, P. M.; Flaherty, D. P., Repurposing the antihistamine terfenadine for antimicrobial activity against Staphylococcus aureus. *J. Med. Chem.* **2014,** *57*, 8540−62.
6. Kabsch, W., Integration, scaling, space-group assignment and post-refinement. *Acta Crystallogr. D* **2010,** *66*, 133−144.
7. Sheldrick, G., SHELXT - Integrated space-group and crystal-structure determination. *Acta Crystallogr. A* **2015,** *71*, 3−8.
8. Sheldrick, G., Crystal structure refinement with SHELXL. *Acta Crystallogr. C.* **2015,** *71*, 3−8.
9. Spek, A., Single-crystal structure validation with the program PLATON. *J. Appl. Crystallogr.* **2003,** *36*, 7−13.
10. Spek, A., PLATON SQUEEZE: a tool for the calculation of the disordered solvent contribution to the calculated structure factors. *Acta Crystallogr. C* **2015,** *71*, 9−18.
